# Highly selective, reversible water activation by P,N-cooperativity in pyridyl-functionalized phosphinines†‡

**DOI:** 10.1039/d3sc05930h

**Published:** 2024-03-14

**Authors:** Richard O. Kopp, Sabrina L. Kleynemeyer, Lucie J. Groth, Moritz J. Ernst, Susanne M. Rupf, Manuela Weber, Laurence J. Kershaw Cook, Nathan T. Coles, Samuel E. Neale, Christian Müller

**Affiliations:** a Institute of Chemistry and Biochemistry, Freie Universität Berlin Fabeckstr. 34/36 14195 Berlin Germany c.mueller@fu-berlin.de; b Department of Chemistry and Materials Innovation Factory, University of Liverpool Crown Street Liverpool L69 7ZD UK; c School of Chemistry, University of Nottingham, University Park Nottingham NG7 2RD UK Nathan.Coles@nottingham.ac.uk; d Department of Chemistry, University of Bath Claverton Down Bath BA2 7AY UK sen36@bath.ac.uk

## Abstract

Tetrapyridyl-functionalized phosphinines were prepared and structurally characterized. The donor-functionalized aromatic phosphorus heterocycles react highly selectively and even reversibly with water. Calculations reveal P,N-cooperativity for this process, with the flanking pyridyl groups serving to kinetically enhance the formal oxidative addition process of H_2_O to the low-coordinate phosphorus atom *via* H-bonding. Subsequent tautomerization forms 1,2-dihydrophosphinine derivatives, which can be quantitatively converted back to the phosphinine by applying vacuum, even at room temperature. This process can be repeated numerous times, without any sign of decomposition of the phosphinine. In the presence of CuI·SMe_2_, dimeric species of the type ([Cu_2_I_2_(phosphinine)]_2_) are formed, in which each phosphorus atom shows the less common μ_2_-bridging 2e^−^-lone-pair-donation to two Cu(i)-centres. Our results demonstrate that fully unsaturated phosphorus heterocycles, containing reactive P=C double bonds, are interesting candidates for the activation of E–H bonds, while the aromaticity of such compounds plays an appreciable role in the reversibility of the reaction, supported by NICS calculations.

## Abstract 2

Aromatic phosphorus heterocycles react at room temperature highly selectively and reversibly with water by P,N-cooperativity. The aromaticity of such compounds plays an appreciable role in the reversibility of the reaction, supported by NICS calculations.

## Graphical abstract

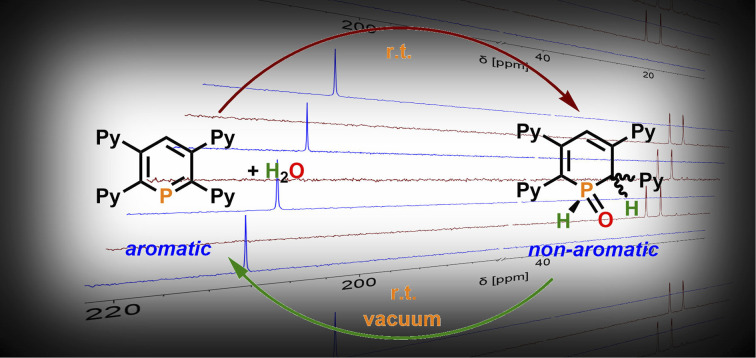

## Introduction

**Fig. 1:**
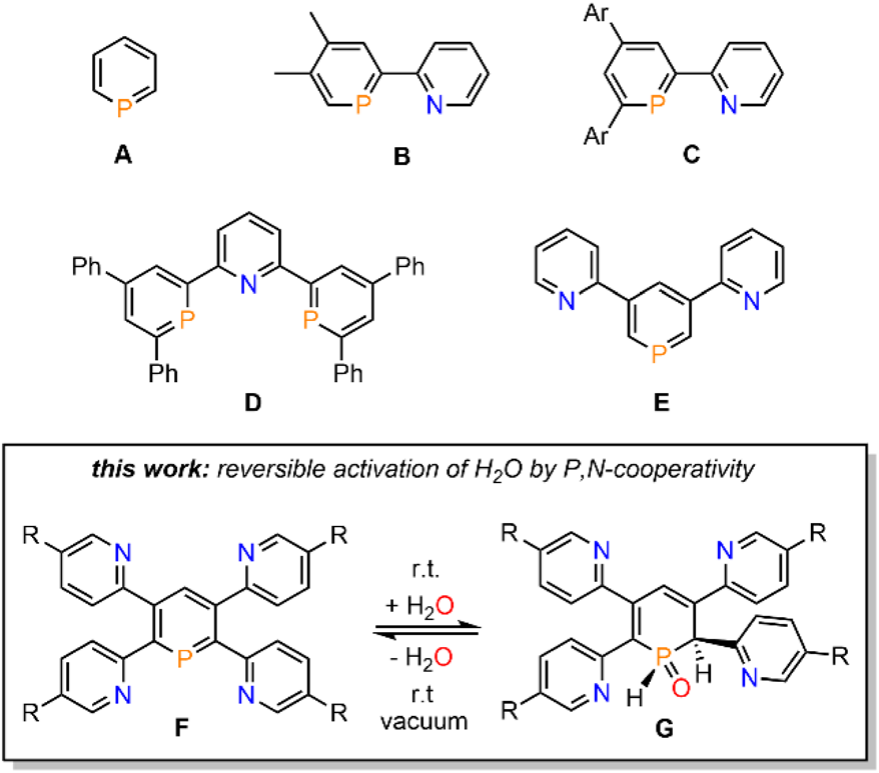
Pyridyl-functionalized phosphinines and brief summary of this work.

Phosphinines (A, Fig. 1), the fully unsaturated six-membered phosphorus heterocycles, are currently undergoing a renaissance as they provide new directions in the fields of coordination chemistry, homogeneous catalysis, activation of small molecules and photoluminescent molecular materials.^1^

Functionalized phosphinines are of particular interest, as the incorporation of additional substituents within the aromatic framework allows for a modification of the stereo-electronic properties of the resulting phosphorus heterocycles. These alterations enable interesting coordination motifs, optical properties, and pronounced reactivities towards small molecules, such as CO_2_, alkynes, alkenes, esters, alcohols or amines.^1*d*,*e*,*f*,*l*^ The development of pyridyl-functionalized phosphinine derivatives, that contain both hard (N-atom) and soft (P-atom) donor atoms (P,N-hybrid ligands), allows generation of novel coordination compounds, which would not be accessible with monodentate phosphinines due to their overall poor net-donor properties.^2^ However, such compounds are rare, as their synthesis can be rather tedious and usually requires multiple steps. The only examples of pyridyl-substituted phosphinines reported to date are summarized in Fig. 1. NIPHOS, a phosphorus derivative of 2,2′-bipyridine (B), was reported by Mathey and co-workers in 1982.^3^ Based on the modular pyrylium-salt route, developed by Märkl in 1966, our group has accessed the pyridyl-phosphinine C and extensively explored its coordination chemistry and reactivity, including metal–ligand-cooperativity to activate small molecules.^1*a*,*l*,4^

In a similar way, a pyridyl-bridged diphosphinine (D) could be prepared, which shows unusual coordination behaviour towards Cu(i) compared to the structurally related terpyridine.^5^ Avarvari, Le Floch and Mathey, who pioneered the 1,3,2-diazaphosphinine route, reported on the polyfunctionalized phosphinine E, containing two remote pyridyl-groups.^6^ During the course of our investigations on the synthesis, coordination chemistry and reactivity of functionalized λ^3^-phosphinines, we found that tetrapyridyl-substituted phosphinines (F) readily and quantitatively react with H_2_O to the corresponding 1,2-dihydrophosphinine oxide derivatives (G). Notably, this reaction turned out to be fully reversible, even at room temperature, and prompted us to explore the H_2_O-addition to the P=C double bond and follow-up chemistry in more detail.

## Results and discussion

**Scheme 1:**
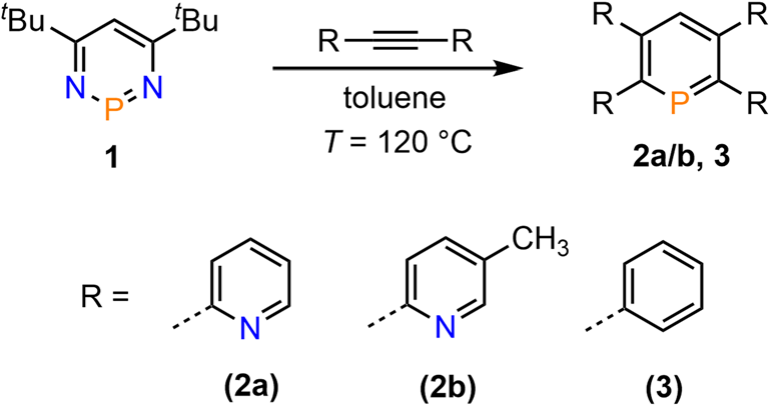
Double [4 + 2]-cycloaddition-cycloreversion reaction for the synthesis of phosphinines 2a/b and 3.

We recently started to investigate the reactivity of λ^3^-phosphinines in small molecule activations. Through this, we uncovered that the nature of the substituent(s) within the aromatic phosphorus heterocycle plays a crucial role in its reactivity towards alcohols and primary amines.^1*d*,2*b*^ Based on our intriguing results in the past on the diverse chemistry of the mono-pyridyl-functionalized phosphinines C and D (Fig. 1), we strived to develop tetrapyridyl-substituted phosphinines 2a/b. For comparative reasons, the structurally related and literature known 2,3,5,6-tetraphenyl-substituted phosphinine 3 was also prepared (Scheme 1).^6^

**Fig. 2:**
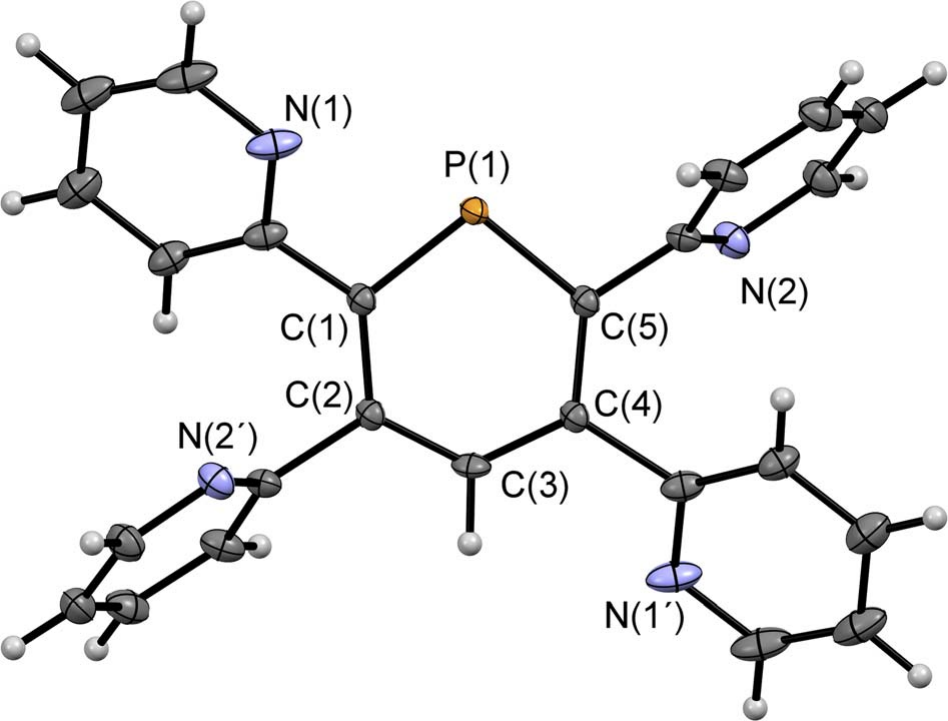
Molecular structure of 2a in the crystal. Thermal ellipsoids given at 50% probability. Atoms marked with ‘are symmetry generated using the following equation: 1−*X*, 1−*Y*, 1−*Z*.

Compounds 2a/b and 3 were synthesized starting from diazaphosphinine 1.^6^ While the commercially available diphenylacetylene was used for the synthesis of 3, the pyridyl-substituted acetylenes 1,2-bis(pyridin-2-yl)ethyne and 1,2-bis(5-methylpyridin-2-yl)ethyne, needed for the preparation of 2a/b, were obtained *via* Stille-coupling from 1,2-bis(tributyltin)-ethyne and the corresponding bromo-pyridines.^7^ Phosphinines 2a/b and 3 were obtained as off-white solids in moderate to good yields and show the typical downfield resonances in their ^31^P{^1^H} NMR spectra (2a: *δ* (ppm) = 213.9; 2b: *δ* (ppm) = 211.3; 3: *δ* (ppm) = 206.2). Single crystals of 2a, suitable for X-ray diffraction, were obtained by layering a solution of 2a in CH_2_Cl_2_ with acetonitrile. Crystals of 2b could also be obtained from slow evaporation of a concentrated toluene solution (Fig. S4.1‡). The results of the X-ray structural analysis of 2a are depicted in Fig. 2. The crystallographic characterization of 2a shows the expected connectivity of the atoms, with the phosphinine core exhibiting inversion disorder across a special position, typical for species which show high symmetry.^8^

While working up the reaction depicted in Scheme 1, a slight colour change was noticed, when a solution of 2a/b in CDCl_3_ was kept open to air. Interestingly, it turned out that the phosphorus heterocycles are very sensitive towards water. This is surprising, as phosphinines typically do not react with H_2_O. The diazaphosphinine 1 is a highly reactive, very air- and moisture sensitive compound and forms a 1,2-dihydro-1,3,2-diazaphosphinine oxide in the presence of traces of H_2_O.^6*a*^ Compound 1 also reacts with HCl under formation of a chlorodiazaphosphacycle.^9^ However, so far only a single example was reported by Le Floch and co-workers, in which an SPS-based pincer-type “classical” phosphinine was found to react with H_2_O.^10^ Initial, yet inconclusive calculations revealed, that the sulfur-functionality of the additional Ph_2_P=S-groups in 2- and 6-position of the phosphorus heterocycle plays an important role in the addition of H_2_O across the P=C double bond (*vide infra*). Indeed, we observed that neither the pyridyl-functionalized phosphinine C (Fig. 1), nor the reference compound 3 (Scheme 1) react with H_2_O, even at elevated temperatures.

**Fig. 3:**
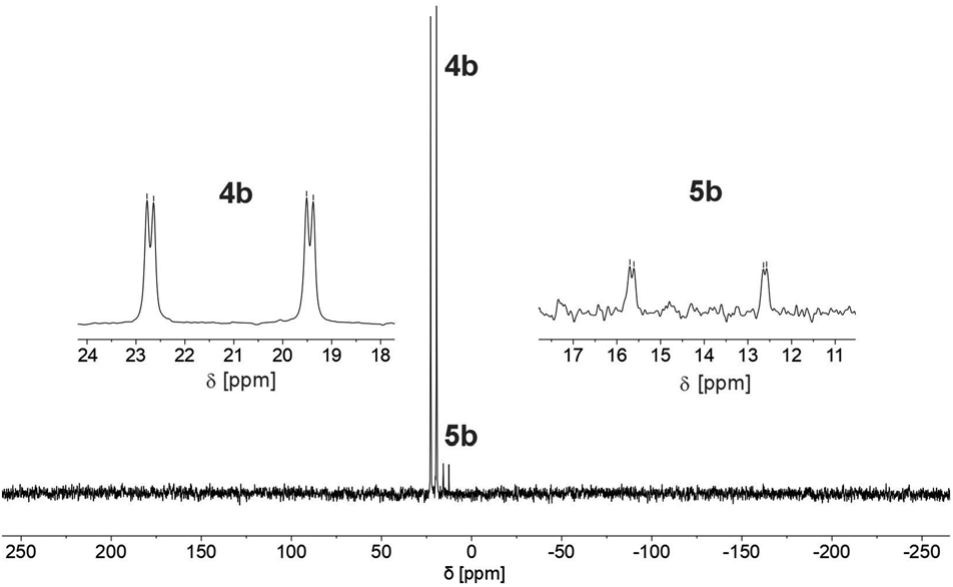
^31^P NMR spectrum for the reaction of 2b with H_2_O in CD_2_Cl_2_.

We therefore decided to investigate this rather intriguing reaction of an aromatic phosphorus heterocycle with H_2_O in more detail. After addition of 10 equiv. of H_2_O to a solution of 2b in CD_2_Cl_2_, the quantitative conversion of the starting material to a new species (4b) was observed after approximately 18 hours. The main product shows a singlet in the ^31^P{^1^H} NMR spectrum at *δ* (ppm) = 21.1 (Fig. 3).

**Scheme 2:**
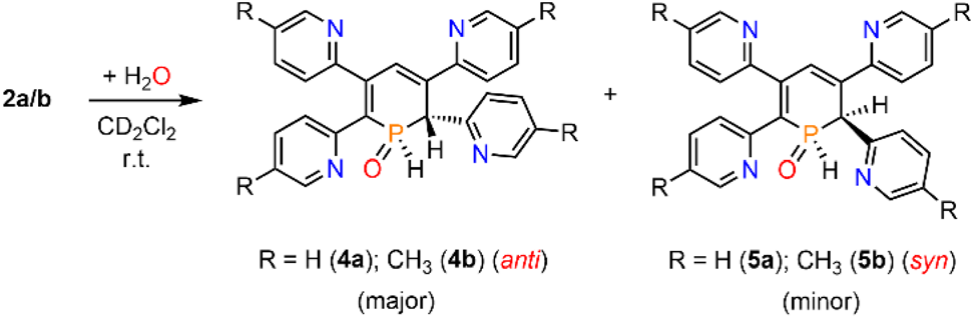
Reaction of 2a/b with H_2_O under formation of diastereomers 4a/b and5a/b.

A second, minor resonance (5b) is formed, which displays a signal in the ^31^P{^1^H} NMR spectrum at *δ* (ppm) = 14.1. In the proton coupled ^31^P NMR spectrum of the reaction mixture, both signals consist of a doublet of doublet, with coupling constants of ^1^*J*_P–H_ = 530 Hz and ^2^*J*_P–H_ = 22.0 Hz for the major product, and ^1^*J*_P–H_ = 498 Hz and ^2^*J*_P–H_ = 16.5 Hz for the minor one (Fig. 3). The final ratio between both species of 97 : 3 remains constant, even after prolonged monitoring the sample for up to two months. This observation, along with ^1^H and ^13^C{^1^H} NMR spectroscopic data (*vide infra*), is consistent with the formation of a diastereomeric pair of 1,2-dihydrophosphinine oxide derivates (4b/5b), depicted in Scheme 2. While a chemical shift difference of Δ*δ* (ppm) = 7 in the ^31^P NMR spectra is rather large when compared to non-cyclised phosphine oxide diastereomers it is comparable to what is observed for diasteromeric phosphole heterocycles, for which chemical shift differences of Δ*δ* (ppm) = ∼10 have been observed in the corresponding ^31^P NMR spectra.^11,12^

For a better understanding of the structural characteristics of the obtained products, additional spectroscopic and crystallographic studies were performed. The assignment of the recorded signals in the ^1^H and ^1^H{^31^P} NMR spectra, in combination with ^13^C{^1^H} NMR spectroscopy and the respective 2D NMR spectroscopic data, led finally to the successful identification of both diastereomers.

**Fig. 4:**
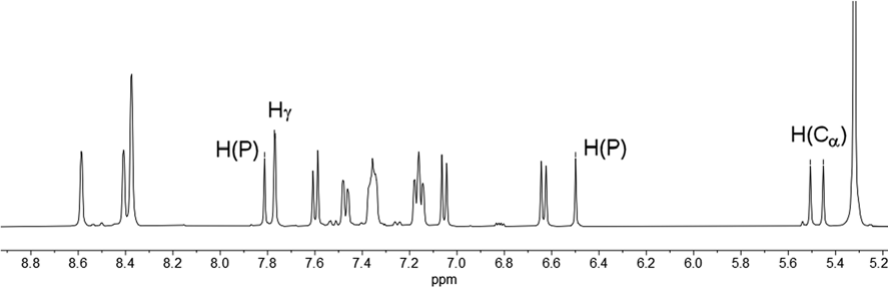
^1^H NMR spectrum of 4b/5b in CD_2_Cl_2_.

In fact, the ^1^H NMR spectrum of the reaction mixture reveals the P–H proton as a doublet resonance with a chemical shift of *δ* (ppm) = 7.16 and a coupling constant of ^1^*J*_H–P_ = 528 Hz (Fig. 4, H(P)/H(P)). This coupling constant is consistent with the coupling constant detected for the major diastereomer signal in the ^31^P NMR spectrum.

The hydrogen atom which shows a ^2^*J*_P–H_ coupling in the ^31^P NMR spectrum can be assigned to the doublet with a chemical shift of *δ* (ppm) = 5.48 and a coupling constant of ^2^*J*_H–P_ = 21.9 Hz in the ^1^H NMR spectrum. This hydrogen atom couples *via*^1^*J*_C–H_ coupling to a tertiary carbon atom at *δ* (ppm) = 46.7 with a coupling constant of ^1^*J*_C–P_ = 64 Hz in the corresponding HMQC spectrum and can therefore be assigned to a C_α_ atom (H(C_α_)). The hydrogen atom in the 4-position of the heterocycle (H_γ_) shows a chemical shift of *δ* (ppm) = 7.77 and a coupling constant of ^4^*J*_H–P_ = 1.8 Hz in the ^1^H NMR spectrum. The chemical inequivalence of the four pyridyl moieties is also observed with chemical shifts of *δ* (ppm) = 2.36, 2.29, 2.23 and 2.22 for the methyl groups in the ^1^H NMR spectrum. These assignments are also in accordance with the recorded phosphorus decoupled ^1^H{^31^P} NMR spectra. Based on these findings, the major species can be assigned to the 1,2-dihydrophosphinine oxide 4b. It is worth noting that the corresponding H(C_α_) cannot be clearly located in the ^1^H and ^13^C{^1^H} spectra for 5b. However, three small methyl signals can be detected, suggesting asymmetry of the methyl groups as expected for 5b (Fig. S2.19‡).

Similar observations also arise for the formation of 4a/5a. The H(C_α_) of 4a is found at *δ* (ppm) = 5.62 in the ^1^H NMR spectrum with a coupling constant of ^2^*J*_H–P_ = 22 Hz. The corresponding carbon signal was found at *δ* (ppm) = 47.4 in the ^13^C{^1^H} spectrum with a coupling of ^1^*J*_C–P_ = 64 Hz. For this species a second set of signals for the minor diastereomer can also be observed. This compound shows a minor doublet-of-doublets at *δ* (ppm) = 5.42 with couplings of ^2^*J*_H–P_ = 17 Hz and ^3^*J*_H–H_ = 3 Hz in the ^1^H NMR spectrum (Fig. S2.13‡). The ^13^C{^1^H} signal appears also as a doublet at *δ* (ppm) = 44.9 with a coupling constant of ^1^*J*_C–P_ = 65 Hz (Fig. S2.14‡).

**Fig. 5:**
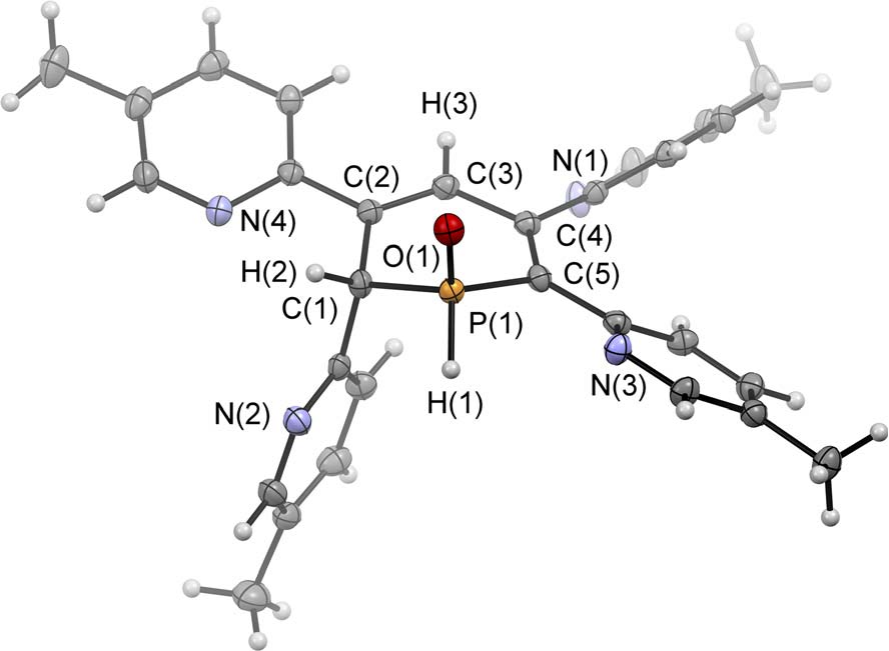
Molecular structure of 4b in the crystal. Displacement ellipsoids are shown at the 50% probability level. Selected bond lengths (Å) and angles (°): P(1)–O(1): 1.482(2); P(1)–C(1): 1.817(2); P(1)–C(5): 1.803(2); C(1)–C(2): 1.510(3); C(2)–C(3): 1.343(3); C(3)–C(4): 1.467(3); C(4)–C(5): 1.364(3); O(1)–P(1)–C(1): 112.9(9); O(1)–P(1)–C(5): 115.0(9); C(1)–P(1)–C(5): 103.6(5); P(1)–C(1)–C(2): 110.78, C(1)–C(2)–C(3): 121.5(2); C(2)–C(3)–C(4): 127.2(2).

Single crystals of the major diastereomer 4b, suitable for X-ray diffraction analysis, were finally obtained from layering a dried sample of 4b in dichloromethane with acetonitrile and subsequent slow evaporation of the solvent. The molecular structure of 4b in the crystal is depicted in Fig. 5, along with selected bond lengths and angles.

The solid-state structure is indeed in accordance with the proposed structure of a 1,2-dihydrophosphinine oxide and related to the reported data of Le Floch *et al.* on their 1,2-dihydrophosphinine oxide.^10^ The major diastereomer shows an *anti*-orientation of the two hydrogen atoms arising from the water addition across the P=C double bond (H–C_α_/H–P) The phosphorus atom adopts the expected tetrahedral geometry with a C(1)–P(1)–C(5) angle of 103.6(5)°. The alternation between the C–C single and double bonds in the heterocyclic ring is evident from the smaller and larger bond lengths between C(1)–C(2)/C(3)–C(4) and C(2)–C(3)/C(4)–C(5), respectively. The P(1)–O(1) bond length of 1.482(2) Å is in accordance with other reported phosphine oxides found in the literature (Ph_3_P=O: 1.46(1) Å).^13^ The minor diastereomer 5b is consequently a species, in which the two hydrogen atoms arising from the water addition are oriented in a *syn*-fashion with respect to one another.

The products of the water addition are stable in the solid state and can be stored under inert conditions for approximately two weeks at room temperature. Much to our surprise, however, we found that upon warming a sample of 4b/5b to *T* = 95 °C, the aromatic phosphinine 2b reforms. At this temperature a ratio of 1 : 9 was detected spectroscopically (2b:4b/5b). A similar observation was made for 4a/5a and phosphinine 2a.

**Fig. 6:**
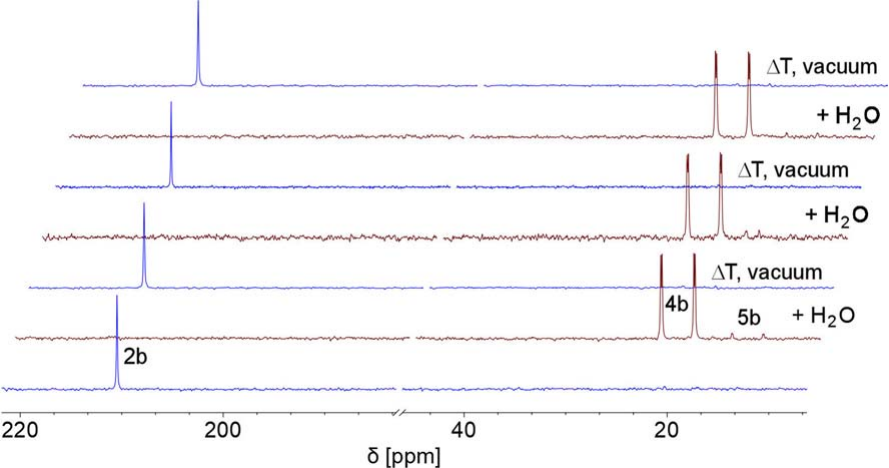
Reversible reaction of 2b with H_2_O to 4b and traces of 5b, monitored by ^31^P NMR spectroscopy in CD_2_Cl_2_ (3 cycles).

**Scheme 3:**
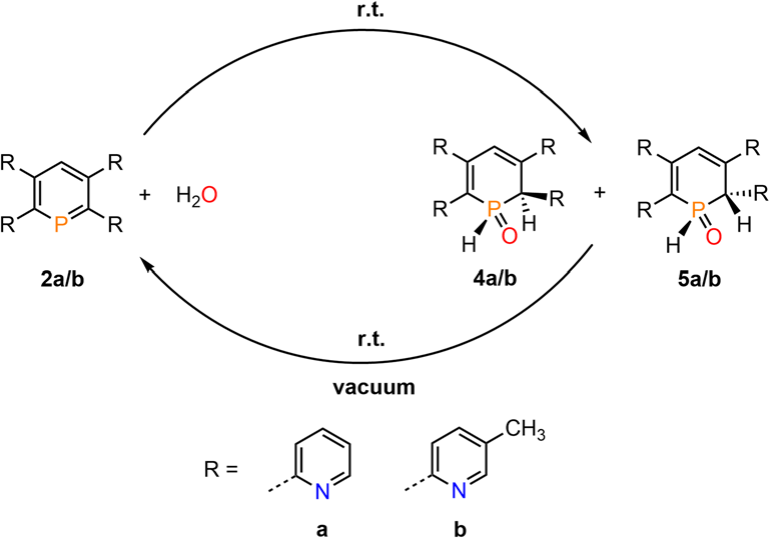
Reversible reaction of 2a/b with H_2_O.

This indicates that the water addition to the P=C double bond is reversible and a slightly exergonic process. Most strikingly, it was possible to render the H_2_O addition to phosphinine 2b fully reversible. When removing the solvent of a freshly prepared solution of 4b/5b under vacuum, a gradual loss of H_2_O and formation of 2b is observed. With additional gentle heating to *T* = 40 °C, the recorded ^31^P and ^1^H NMR spectra of the residue show the exclusive presence of phosphinine 2b (Fig. 6 and Scheme 3).

Measuring the ^31^P NMR spectrum against an internal standard revealed a quantitative spectroscopic yield. The procedure can be repeated numerous times (Fig. 6 and Scheme 3), without the formation of any detectable side or decomposition products. A similar situation was observed for the re-formation of 2a from 4a/5a. However, after several cycles, the formation of traces of impurities could be detected by means of ^31^P NMR spectroscopy for this particular case.

To review the sensitivity of the water addition, the reaction between 2a and varying equivalents of H_2_O was monitored *via* UV/vis spectroscopic measurements (ESI,‡ Chapter 3). Despite the extremely low concentrations of these samples, the formation of 4a/5a could still be observed in form of a new absorption signal at *λ* = 355 nm when using more than five equivalents of water (Fig. S3.1–3‡).

Apart from these spectroscopic insights, we further found a correlation between the time needed for full conversion of 2b with H_2_O and the nature of the solvent. While the total reaction time both in dichloromethane and ethanol amounted to 18 h (*vide supra*), it could be significantly reduced when changing the solvent for DME, DMF, DMSO, (4 h each) pyridine (5 h) and methanol (8 h). Interestingly, the use of chloroform resulted in a complete conversion after only 30 min. A correlation between the relative solvent polarities and the reaction times was, on the other hand, not found.^14^ A change in the final product ratio between the diastereomers 4b/5b and (97 : 3) was not observed either. Analogous observations were made for the reaction of 2a with H_2_O.

We could demonstrate here for the first time, that a phosphinine can fully reversibly react with H_2_O, even at room temperature. Neither a reversible H_2_O-addition to the diazaphosphinine 1, nor to the Ph_2_P=S-substituted phosphinine have been reported (*vide supra*). We believe, that our findings are particularly interesting, as reports on the reversible activation of small molecules by main group compounds are still extremely rare. Typically, geometrically constrained phosphorus(iii) compounds, such as those reported by Radosevich, Goicoechea and others, have proven to be excellent candidates for the activation of E–H bonds. However, in most cases, these transformations are usually irreversible.^15^ Nevertheless, a reversibility of such processes is a key to the development of catalytic reactions based on elements other than transition metals.

**Scheme 4:**
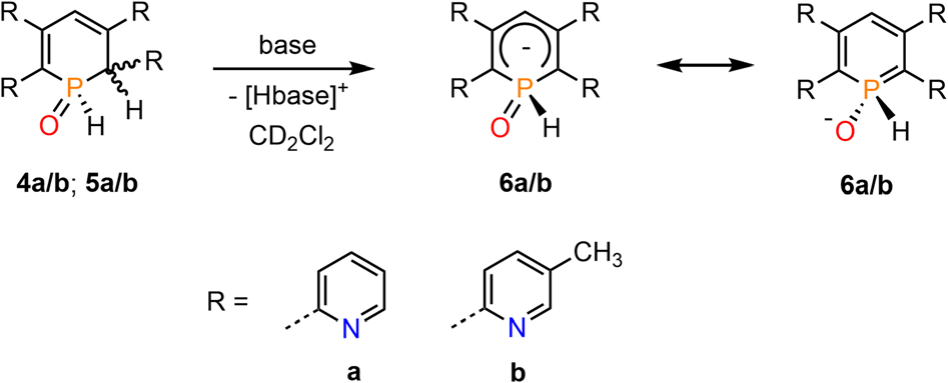
Deprotonation of 5a/b (6a/b) with a base under formation of 7a/b.

We have recently shown that water-addition products of cationic Rh(iii) and Ir(iii) complexes, containing the 2,4-diphenyl-6-pyridylphosphinine (C, Fig. 1, Ar=Ph) as ligand, can undergo deprotonation reactions upon addition of a base.^16^ We were therefore interested whether 4a/b (5a/b) can be converted into the related λ^5^-phosphinin-1-olates 6a/b as well (Scheme 4).

We first applied relatively weak bases such as pyridine and imidazole in excess. As this led to no conversion of the starting material 4b/5b, the bases were changed for the considerably stronger isopropylamine and triethylamine. The attempt with *i*PrNH_2_ resulted in 80% conversion to 6b after 1 h but the product started to decompose shortly after. Reaction of 4b/5b with stoichiometric amounts of NEt_3_ at room temperature gave only 20% conversion of the starting material after 3 days, while gentle heating to *T* = 40 °C led to decomposition of the sample. When using an excess of 10 equiv. of NEt_3_, the conversion could be increased to 40% after 2 h but did not further proceed over the next few days. The base was therefore changed again, now using the slightly more basic piperidine. In a first attempt an excess of 10 equiv. of base immediately led to decomposition of the starting material. When only 2.0 equiv. of piperidine were used, an instantaneous conversion of the starting material to phosphininolate 6b was observed in the proton-coupled ^31^P NMR spectrum, also indicated by an immediate colour change from yellow to dark red. The product depicted a doublet for the P–H bond with a chemical shift of *δ* = 7.74 ppm and a P–H coupling constant of ^1^*J*_P–H_ = 527 Hz. However, the NMR spectra showed the formation of at least three side-products, whose amounts increased over time relative to 6b. After 5 h, 6b was completely decomposed.

**Fig. 7:**
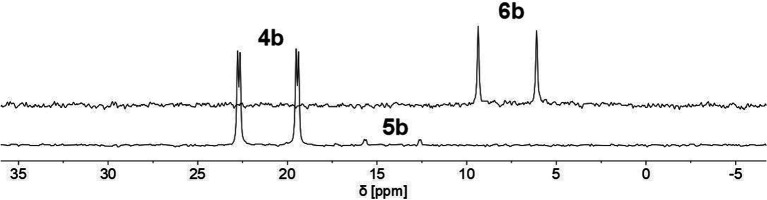
^31^P NMR spectra (CD_2_Cl_2_) of 1,2-dihydrophosphinine oxide 4b (bottom) and its deprotonation product, phosphinine-1-olate 6b, after reaction with LDA (top).

Finally, the use of lithium diisopropyl amide (LDA) resulted in the complete conversion of 4b/5b and sole formation of phosphininolate 6b, as evidenced by ^31^P NMR spectroscopy. The newly observed doublet with a chemical shift of *δ* (ppm) = 7.7 exhibits only a ^1^*J*_P–H_ coupling constant of 528 Hz, confirming the successful elimination of the proton at the α-carbon atom. More importantly, only one product signal was obtained in the recorded NMR spectrum (Fig. 7).

A strong bright orange fluorescence of the sample, which could be stored at *T* = −21 °C for several days, was also noticed. It should be noted here that λ^5^-phosphinines often show strong fluorescence, while their electronic structure has been addressed computationally.^17^ An analogous reaction of 4b/5b with 1.1 equiv. of *n*-BuLi allowed for isolation of the product as a bright red powder in 90% yield. The product signals could be completely assigned by means of 2D NMR spectroscopy (^1^H/^13^C HMQC/HMBC). The spectroscopic analysis confirmed the proposed structure of 6b. Additionally, the formation of the symmetrical λ^5^-phosphinine 6b compared to the starting material leads to the overall reduced number of signals in the recorded ^1^H NMR spectrum of 6b, indicating a larger number of chemically equivalent nuclei. More importantly, when comparing the aromatic region of the ^1^H NMR spectrum of 6b to the one of 4b/5b, the lack of a signal for the hydrogen atom in the 2-position is indeed noticeable. Simultaneously, the signal belonging to the phosphorus-bound hydrogen atom is significantly down-field shifted in 6b, while the C(4)-bound hydrogen atom H_γ_ is strongly shifted upfield compared to the one in 4b/5b. Similar observations were made in the deprotonation of 4a/5a.

**Scheme 5:**
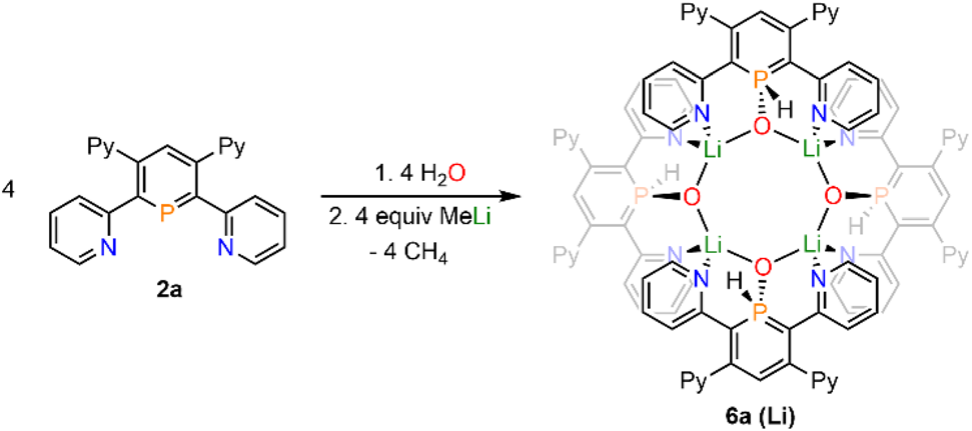
Reaction of 2a with H_2_O and MeLi and formation of tetramer 6a(Li).

**Fig. 8:**
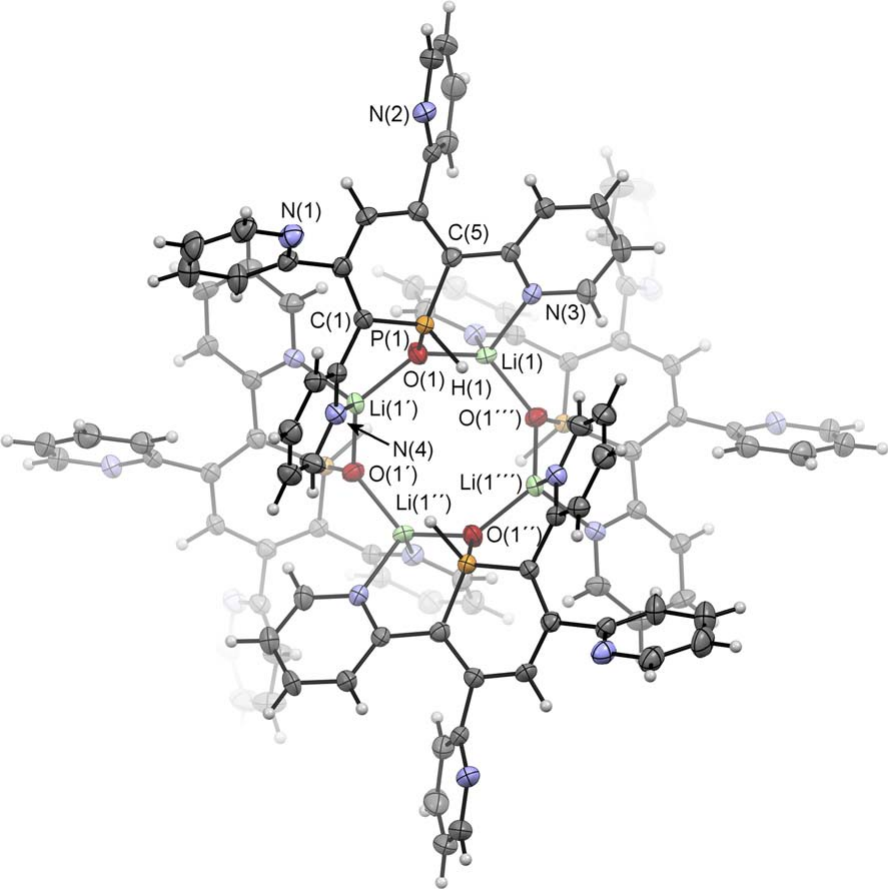
Molecular structure of 6a(Li) in the crystal. Thermal ellipsoids given at 50% probability. Atoms marked with ′are symmetry generated using the following equation: 1−*Y*, +*X*, 2−*Z*. Atoms marked with ′′ are symmetry generated using the following equation: 1−*X*, 1−*Y*, +*Z*. Atoms marked with ′′′ are symmetry generated using the following equation: +Y, 1-X, 2-Z. Selected bond lengths (Å) and angles (°): P(1)–O(1): 1.514(4); P(1)–C(1): 1.764(5); P(1)–C(5): 1.752(5); O(1)–Li(1): 1.916(8); O(1)–Li(1′): 1.897(9); N(3)–Li(1): 2.040(10); N(4)–Li(1′): 2.130(9). C(1)–P(1)–C(5): 105.0(2).

Upon adding first H_2_O and subsequently MeLi/Et_2_O to a solution of 2a in CD_2_Cl_2_ we could finally obtain needle-shaped orange, slightly fluorescent crystals of 6a(Li) after a few hours from the reaction mixture. The crystallographic representation of this compound shows that 6a(Li) exists as a tetramer in the solid state, which accounts for its very low solubility in common solvents (Scheme 5 and Fig. 8).

**Fig. 9:**
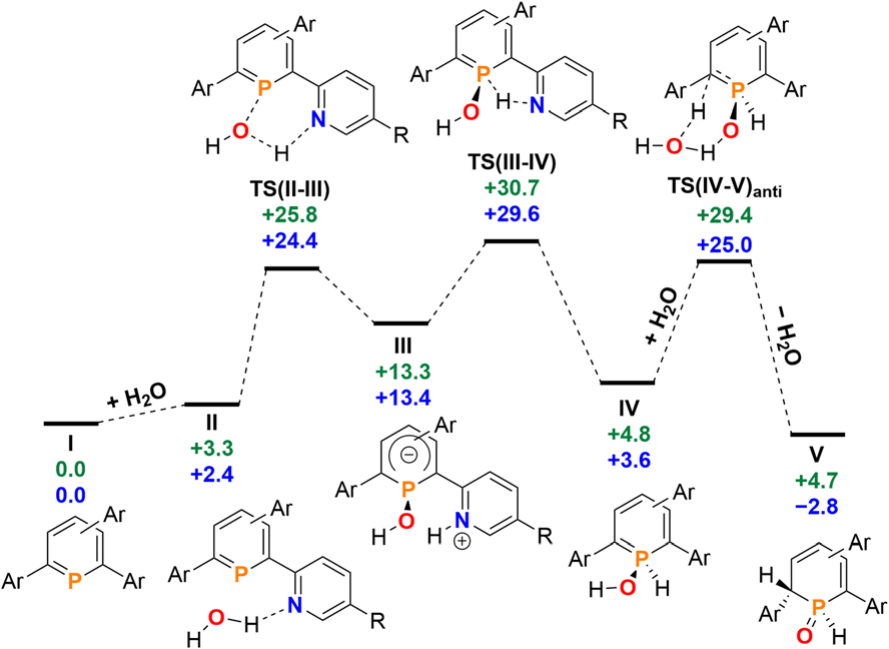
Free energy profile (calculated with DFT at the M052X-D3(CPCM=CH_2_Cl_2_)/def2-QZVPP//TPSS/def2-TZVPP level of theory, energies in kcal mol^−1^) of H_2_O addition at 2b (blue) and at 2,4-phenyl-6-pyridylphosphinine (C, green). Note: each structure is labelled with a subscript “2b” (*i.e.*I_2b_) and “C” (I_C_) in the ESI‡ to distinguish between the identity of the phosphinine.

Having investigated the reaction of 2a/b with H_2_O in detail, we anticipated that other small molecules, such as alcohols, could be activated in a similar manner. Phosphinine 2b was dissolved in methanol, serving as the reactant and the solvent at the same time, and the reaction was monitored by means of ^31^P NMR spectroscopy at room temperature over several days. Much to our surprise, no reaction between 2b and methanol was observed. The same result was obtained under similar conditions for the attempted reaction of 2b with ethanol, as well as with the more acidic phenol in DMSO. Phosphinine 2b thus turned out to exclusively activate H_2_O. As mentioned above, this is in clear contrast to most phosphinine metal complexes and phosphinine sulfides/selenides, which generally react with a multitude of protic reagents. To probe the differences in H_2_O addition reactivity between the tetrapyridyl-functionalized phosphinine (2b), 2,4-diphenyl-6-pyridylphosphinine (C), and the reference compound 2,3,5,6-tetraphenylphosphinine (3), a computational mechanistic study was undertaken using density functional theory (at the M052X-D3(CPCM=CH_2_Cl_2_)/def2-QZVPP//TPSS/def2-TZVPP level of theory). The resulting characterized free energy profile (in kcal mol^−1^) for the addition and subsequent formal oxidative addition of the O–H bond to the phosphorus atom of 2b and 2,4-diphenyl-6-pyridylphosphinine (C) is presented in Fig. 9.

**Fig. 10:**
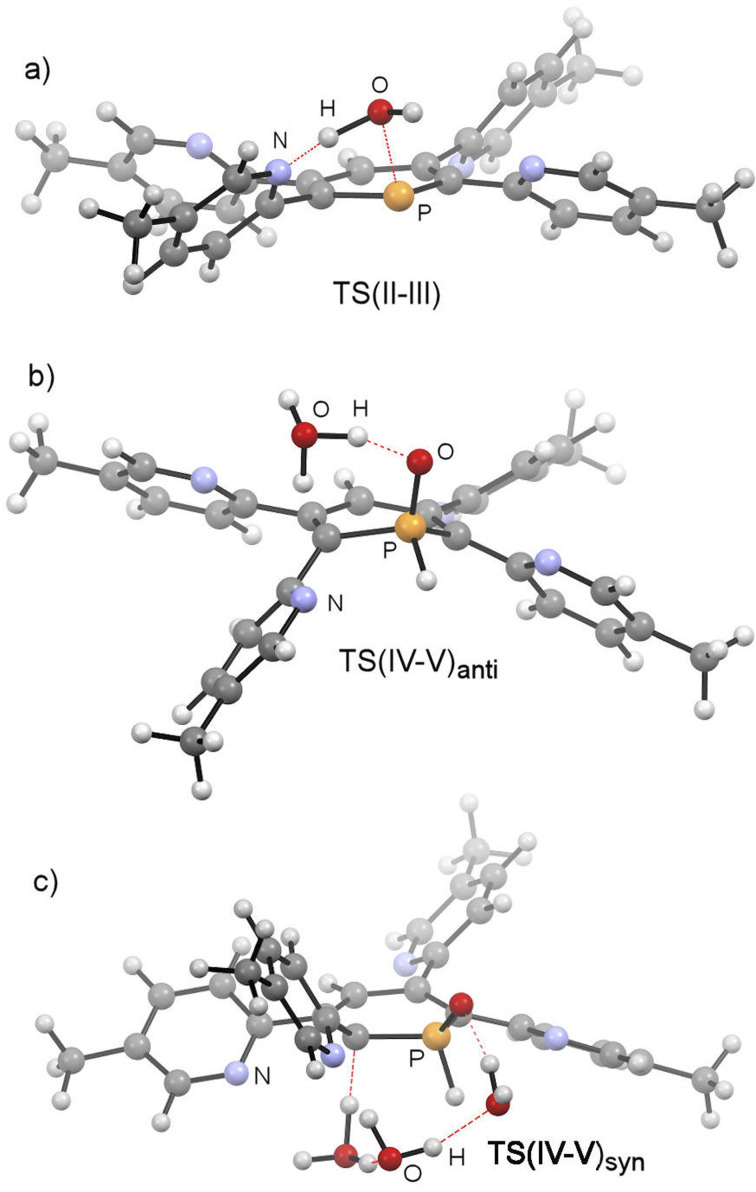
Structures of TS(II–III), TS(IV–V)_anti_ and TS(IV–V)_syn_ at 2b.

The mechanistic study focussed first on the initial interaction of H_2_O with the low-coordinated phosphorus centre *via* one of the oxygen lone-pairs. We anticipated that a subsequent formal oxidative addition of the O–H bond to the phosphorus atom occurs at the tetramethyl-tetrapyridyl-phosphinine I (2b). The corresponding phosphinin-1-ol then undergoes fast tautomerization, leading to the experimentally observed 1,2-dihydrophosphinine oxide derivatives.^9^ A direct oxidative addition at the phosphorus centre *via* a three-membered transition state was first considered and modelled, but subsequently discounted given the excessively high energy of the corresponding transition state, TS(OA) (+62.8 kcal mol^−1^, Fig. S2d‡). This led to the consideration that the flanking pyridyl groups serve to kinetically enhance the formal oxidative addition process *via* H-bonding, given that pyridine, and its related analogues, are competent H-bond acceptors. We indeed found that the approaching H_2_O to I can form the H-bonded adduct II (+2.4 kcal mol^−1^, Fig. 9). Subsequently, deprotonation of H_2_O along with the transfer of the hydroxyl group to the phosphorus atom and formation of a zwitterionic species occurs *via*TS(II–III) (+25.8 kcal mol^−1^, Fig. 10a). This is supported by the fact, that nucleophilic attack at the low-coordinate phosphorus atom in phosphinines (energetically low-lying LUMO) is well documented in the literature.^1*d*,18^ Thus, this characterised mode of H_2_O addition by P,N cooperativity *via* complementary participation of the formal electrophilic (Lewis acidic) phosphorus centre, and the nucleophilic (Lewis basic) pyridyl-nitrogen centre, appears somewhat resemblant of reported modes of H_2_O activation by intramolecular Frustrated Lewis Pairs (FLPs).^19^

Participation of further equivalents of H_2_O *via* H-bonded networks of two (TS(II–III)_2_: +26.7 kcal mol^−1^), or three equivalents of H_2_O (TS(II–III)_3_: +34.1 kcal mol^−1^) to further accelerate this step were also considered and modelled (Fig. S1‡).

This was, however, found to be kinetically disfavoured with respect to TS(II–III). From III, H^+^ transfer from the protonated pyridyl group to the lone-pair at the phosphorus atom takes place *via*TS(III–IV) (+29.6 kcal mol^−1^) to form IV (+3.6 kcal mol^−1^), which is a λ^5^-phosphinin-1-ol and the formal oxidative addition product of one O–H bond of H_2_O to the phosphorus atom. This step is feasible, as the phosphorus atom in III becomes much more nucleophilic after addition of OH^−^, and the remaining phosphorus lone-pair can now easily take up a proton to form IV. Moreover, the dominant displacement of the proton in the large imaginary mode (1267i cm^−1^) upon frequency analysis of TS(III–IV), suggests a degree of proton tunnelling that can also serve to accelerate this process and account for the observed reactivity already at room temperature. From IV, a final and fast de-aromatizing H-transfer process occurs *via*TS(IV–V)_anti_ (+25.0 kcal mol^−1^, Fig. 10) to form the secondary 1,2-dihydro-phosphine oxide (SPO) species V (−2.8 kcal mol^−1^), which is the experimentally observed 1,2-dihydrophosphinine oxide 4b as the major species However, unlike the initial formal oxidative addition process, this step was identified to be favoured in the presence of an additional H_2_O, thus forming a network to facilitate proton shuttling through an ordered six-membered transition state. An alternatively considered four-membered transition-state was kinetically disfavoured (TS(4M), +37.4 kcal mol^−1^, Fig. S1.2e‡). Moreover, TS(IV–V)_anti_ (Fig. 10b) rationalizes the observed *anti*-selectivity (H–C_α_/H–P) of the addition, where the second water molecule is situated perfectly to allow hydrogen bonding to the P=O moiety and subsequent proton transfer. To confirm the favourability of *anti*-selectivity, a competing process of *syn*-selective product formation *via* proton-shuttling mechanism with a network of three water molecules was considered and computed (Fig. 10c). The corresponding transition state (TS(IV–V)syn, +36.9 kcal mol^−1^) and product (V_syn_, +0.1 kcal mol^−1^) were found to be 11.9 kcal mol^−1^ and +3.1 kcal mol^−1^ higher in energy than TS(IV–V)_anti_, and so further supports the experimentally observed *anti*-selectivity for the preferred formation of the secondary phosphine oxide species 4b (see ESI‡ for further details), while the diastereomer 5b is formed as the minor species.

To survey the influence of phosphinine aromaticity on the reversibility of water addition at 2b, NICS(0) and NICS(+1/−1) calculations were performed for I_2b_, IV_2b_, and V_2b_ (B3LYP/def2-TZVPP level, see the ESI‡ for full details). For I_2b_, appreciable negative NICS(+1/−1) values are found (−7.3/−7.3), identifying the starting phosphinine species as aromatic (*cf.* C_6_H_6_, NICS(1) = −10.0), which supports other computational studies of aromatic properties of phosphinines.^20^ Calculations also indicate that upon H_2_O addition to form λ^5^-phosphinin-1-ol IV_2b_ some partial loss of aromaticity occurs (NICS(+1/−1) = −2.4/−3.9), which is in line with computational studies on λ^5^-phosphinines.^17^ As expected, no discernible aromaticity is quantified for the ultimate 1,2-dihydrophosphinine oxide product 5b/V_2b_ (NICS(+1/−1) = +0.5/−0.3), indicating the reversibility of P(III)/P(v) redox driven water activation is at least partially influenced by an aromaticity loss/recovery sequence.

Re-computation of these reaction steps with 2,4-diphenyl-6-pyridylphosphinine (C) indicates that the initial formal oxidative addition *via*TS(II–III) is accessible at +25.8 kcal mol^−1^. While only +2 kcal mol^−1^ in energy higher than calculated for 2b, the rate-limiting H^+^ transfer from the nitrogen atom to the phosphorus donor *via*TS(III–IV) lies at +30.7 kcal mol^−1^. Moreover, the formation of the final 1,2-dihydrophosphinine oxide V (+4.7 kcal mol^−1^) indicates that the overall reaction is now endergonic, as opposed to the exergonic one related to the reaction I → VI in case of H_2_O. Based on these calculations we currently propose that the experimentally observed lack of reactivity of H_2_O with 2,4-phenyl-6-pyridyl-phosphinine can be explained by two factors. Firstly, the slightly higher rate-limiting barrier for this phosphinine results in a kinetic inhibition, even if a pyridyl group is still present to accelerate the initial formal oxidative addition process. Secondly, the overall endergonicity associated with the reaction of I to VI renders the reaction thermodynamically unfavourable. For the reference compound 2,3,5,6-tetraphenylphosphinine (3), an analogous deprotonation-oxidative addition process can obviously not be postulated as no *ortho*-pyridyl groups are present. The only oxidative addition process successfully computed with this species was *via* a 3-coordinate saddle point, analogous to TS(OA) (Fig. S1.2c‡). However, the corresponding transition state (+65.1 kcal mol^−1^), was found to be kinetically disfavoured. This rationalizes why the formation of 1,2-dihydrophosphinine oxides is not observed.

Given that only the *ortho* pyridyl groups appear required to facilitate the water activation reaction *via* H-bonding, re-computation of the reaction steps with a hypothetical 2,6-dipyridylphosphinine (labelled I_2c_) was undertaken to discern the role of the rear *meta*-pyridyl groups in 2a/2b (see the ESI‡ for full details).^21^ The kinetics of H_2_O addition at I_2c_ are almost quantitatively consistent with the tetrapyridyl-substituted phosphinine 2b (where rate-limiting TS(III–IV)_2c_ rests at +29.5 kcal mol^−1^ above reactants I_2c_ and H_2_O, *cf*. TS(III–IV)_2b_ = +29.6 kcal mol^−1^). However, the relative free energy of V_2c_ (+2.9 kcal mol^−1^) indicates that its formation is thermodynamically disfavoured under equilibrating conditions, and thus identifies the importance of the rear 3,5-pyridyl groups to stabilize the formation of 5a/5b over 2a/2b. We anticipate that in the presence of both *meta*-pyridyl groups, the {H}-transfer to form 5a/5b alleviates some steric clashing between *ortho* and *meta* substituents which gives the process further exergonicity that is otherwise not present without them.

It should be mentioned here that our mechanistic studies on the activation of H_2_O by phosphinines are completely different compared to the commonly accepted mechanisms for O–H cleavage reactions by other main group-based compounds, such as carbenes or silylenes.^22^ In these systems, the strong nucleophilic character of the main-group element can cause the deprotonation of H_2_O first, followed by reaction with the remaining OH^−^. Based on our DFT calculations, the mode of H_2_O activation by 2a/b is therefore a concerted deprotonation of H_2_O by the basic pyridyl arm, along with P–OH formation.

**Fig. 11:**
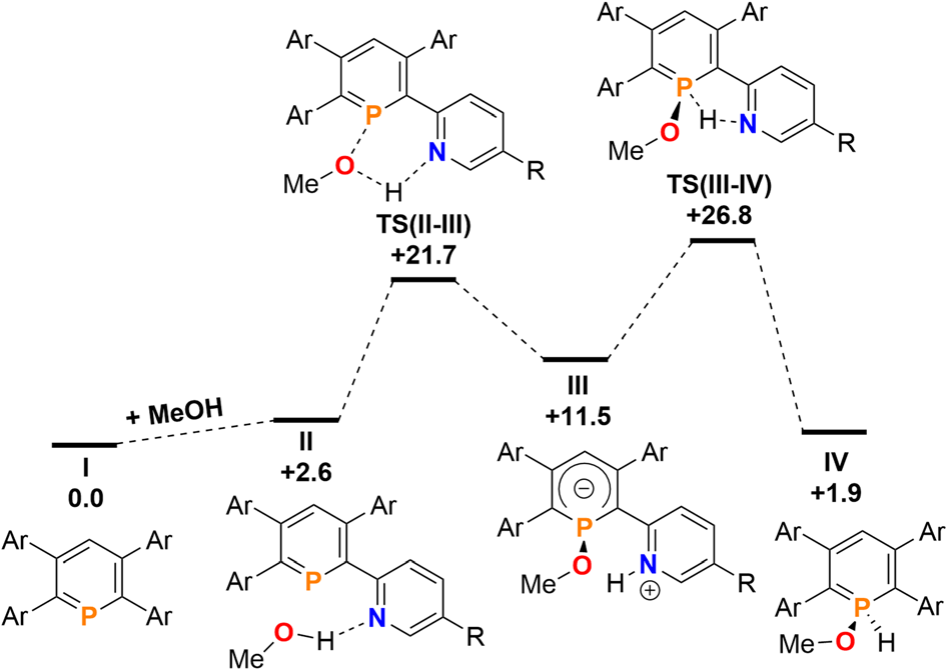
Free energy profile (calculated with DFT at the M052X-D3(CPCM=CH_2_Cl_2_)/def2-QZVPP//TPSS/def2-TZVPP level of theory, energies in kcal mol^−1^) of MeOH addition at 2b.

Finally, the addition and splitting of MeOH was computed at 2b to probe why this reactivity is not observed (Fig. 11). We found, that an initial O–H cleavage process *via*TS(II–III) (+21.7 kcal mol^−1^) takes place to form III (+11.5 kcal mol^−1^), with subsequent rate-limiting H^+^ transfer *via*TS(III–IV) (+26.8 kcal mol^−1^) to yield the formal oxidative addition product IV (+1.9 kcal mol^−1^). Consequently, the calculations indicate that the overall reaction is endergonic and reversible, despite the fact that this process is highly kinetically accessible with a low overall energetic span. Under equilibrating conditions, only a minor amount of IV should be observed experimentally as a consequence.

Having synthesized the novel tetrapyridyl-functionalized phosphinines and having explored in detail their reactivity towards H_2_O, we were also interested in the coordination chemistry of 2a/b and the reactivity of the corresponding complexes. These heterocycles possess multiple coordination sites displaying both hard (N) and soft (P) donor atoms, according to Pearson's HSAB concept. In fact, 2a/b can also be regarded as a phosphorus derivative of terpyridine (inverse of D, Fig. 2), with two additional remote pyridyl-groups in the *meta*-position. Because coinage metal complexes of phosphinines are well known, we focussed on investigated the coordination chemistry of 2a/b towards Cu(i).^23^

**Scheme 6:**
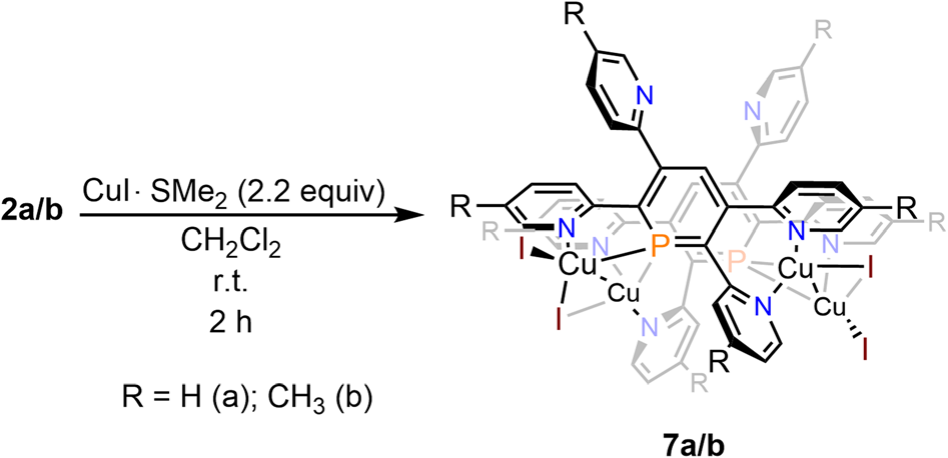
Reaction of 2a/b with CuI·SMe_2_ in CH_2_Cl_2_.

Reaction of 2a with CuI·SMe_2_ in dichloromethane leads to a red solution, which shows a sharp, single resonance at *δ* (ppm) = 154.3 in the ^31^P{^1^H} NMR spectrum (Scheme 6).

The upfield-shift of approximately Δ*δ* (ppm) = 60 in the ^31^P{^1^H} NMR spectrum compared to the starting material 2a indicates the presence of a phosphinine-Cu(i) complex (7a), with coordination *via* the phosphorus atom to the metal centre. The ^1^H NMR spectrum shows a complex, yet very distinct splitting pattern of the signals in the aromatic region (*vide infra*).

**Fig. 12:**
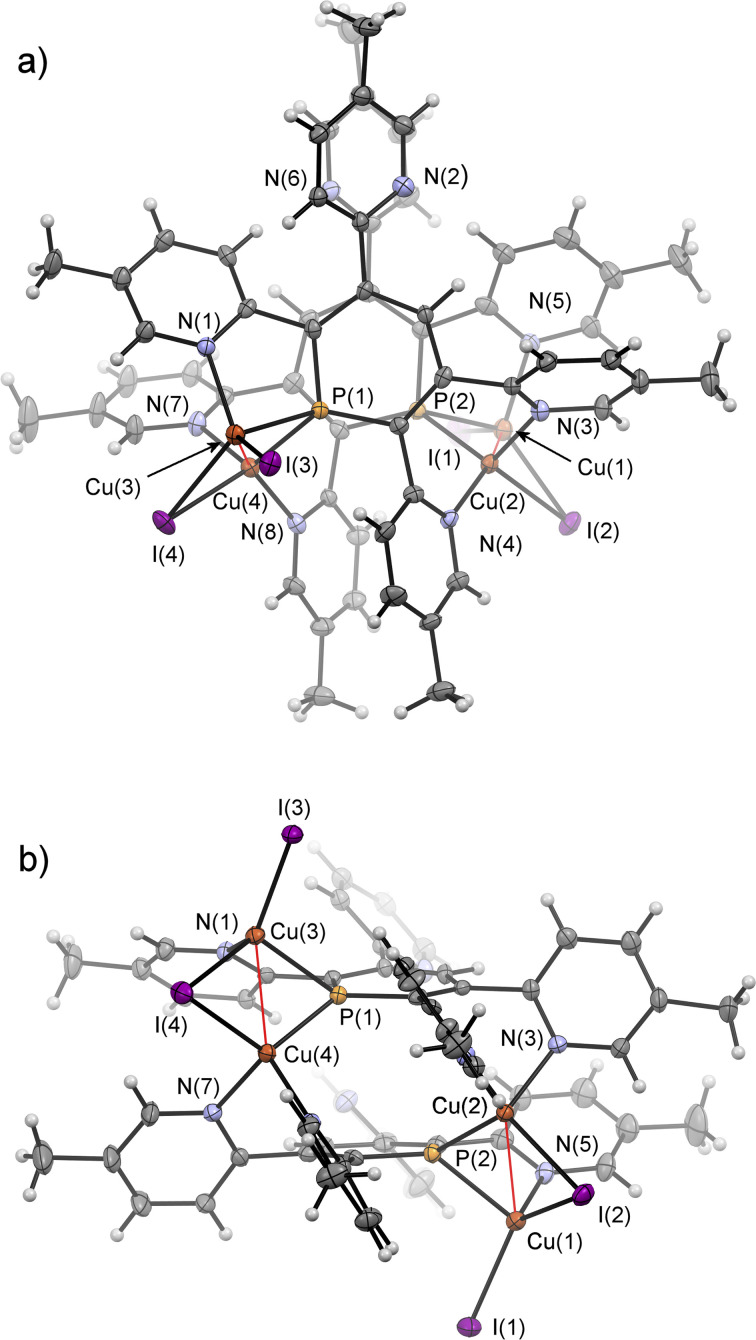
Top (a) and side-view (b) of the molecular structure of 7b in the crystal. Displacement ellipsoids are shown at the 50% probability level. Selected bond lengths (Å) and angles (°): P(1)–C(1): 1.748(4); P(1)–C(5): 1.735(5); P(1)–Cu(3): 2.3123(12); P(1)–Cu(4): 2.2354(12); Cu(3)–Cu(4): 2.5608(8); Cu(3)–I(3): 2.5029(6); Cu(3)–I(4): 2.6268(6); Cu(4)–I(4): 2.683(6); N(1)–Cu(3): 2.097(4); N(3)–Cu(2): 2.072(4); N(4)–Cu(2): 2.022(4); N(7)–Cu(4): 2.077(4); N(8)–Cu(4): 2.033(4). C(1)–P(1)–C(5): 105.9(2); Cu(3)–P(1)–Cu(4): 68.52(4).

Crystals of 7b, suitable for single crystal X-ray diffraction, were obtained by slow diffusion of pentane into a dichloromethane solution of 7b. The molecular structure of 7b in the crystal, along with selected bond lengths and angles, is depicted in Fig. 12. Remarkably, the crystallographic characterization reveals the presence of a dimeric sandwich-type structure for 7b with the general composition [Cu_2_I_2_(2b)]_2_. In 7b each phosphorus atom shows the less common μ_2_-bridging coordination mode to two Cu(i)-centres, which are additionally linked by an I^−^ ligand.^1*b*,*g*,2*a*,23*a*,26^ One of the Cu(i) centres of each Cu_2_-core carries a terminal I^−^ ligand. The edistorted tetrahedral coordination environment of each Cu(i) centre is completed by the phosphinine pyridyl moieties, one pyridyl group for the Cu bearing the terminal I^−^ and two coordinated pyridyl groups for the remaining Cu centres. One *meta*-pyridyl group of each phosphorus heterocycle remains uncoordinated.

**Fig. 13:**
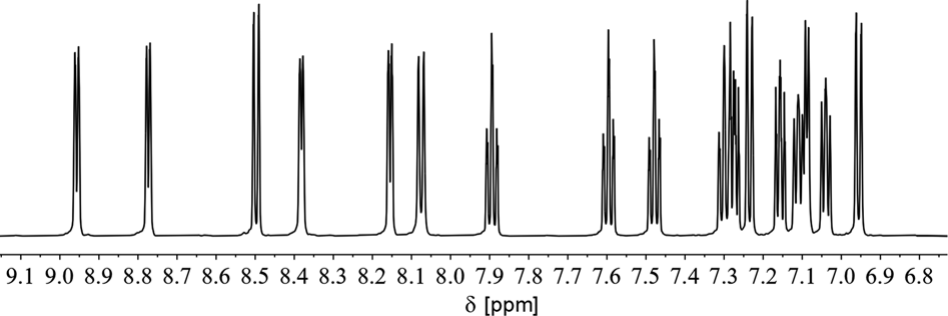
^1^H NMR spectrum of the aromatic region of complex 7a in CD_2_Cl_2_.

Fig. 13 shows the ^1^H NMR spectrum of the aromatic region of 7a in CD_2_Cl_2_. It is possible to assign 16 resonances to the protons of both coordinated and uncoordinated pyridyl-groups as well as the remaining doublet at *δ* (ppm) = 7.09 to the proton in *para*-position of the phosphorus heterocycle (^5^*J*_H–P_ = 5.4 Hz).

This indicates, that the dimeric structure is present both in solution and in the solid state. Similar NMR spectra were obtained from the reaction of 2b with CuI·SMe_2_.

Interestingly, coordination compounds 7a/b are remarkably stable towards air and moisture. This contrasts most phosphinine-based metal complexes as well as phosphinine-sulfides/selenides, which normally react readily with traces of protic reagents at the P=C double bond.^24,25^ To the best of our knowledge, reactivity studies on phosphinine-metal complexes, in which the phosphorus atom shows the less common μ_2_-coordination mode have not yet been performed. We anticipate, that the reactivity of such complexes towards protic solvents might be completely different compared to complexes, in which the phosphinine shows the more common η^1^-as well as the π-coordination mode.^1*b*,*g*,2*a*,23*a*,26^ Studies in this direction will be part of future investigations.

## Conclusions

We have developed novel tetrapyridyl-substituted λ^3^-phosphinines by performing a double cycloaddition–cycloreversion reaction of diazaphosphinine with 1,2-bis(pyridyn-2-yl)ethyne and 1,2-bis(5-methylpyridyn-2-yl)ethyne, respectively. In contrast to other substituted phosphinines, the title compounds are highly sensitive towards H_2_O and selectively form the corresponding 1,2-dihydrophosphinine oxides, with an *anti*-arrangement of the two newly implemented hydrogen atoms as the major compound. In the presence of lithium diisopropyl amide, *n*-BuLi or MeLi, the water addition products can be quantitatively deprotonated, generating the corresponding strongly fluorescent λ^5^-phosphininolates, which show a tetrameric structure in the solid state. Most strikingly, however, the addition of H_2_O to the novel phosphinines is fully reversible, as loss of H_2_O and complete re-generation of the phosphorus heterocycles is observed by gentle heating and placing the samples under vacuum. This process can be repeated numerous times without the formation of any detectable side or decomposition products. Moreover, phosphinines 2a/b serve as a water-sensor, as only H_2_O is activated. Computational mechanistic studies using density functional theory suggest that the selective and reversible water addition to the P=C double bond of the phosphinine most likely occurs *via* P,N-cooperativity, as the initial formal oxidative addition of the O–H bond to the phosphorus atom at the tetrapyridyl-phosphinine is supported by the flanking pyridyl groups serving to kinetically enhance this process *via* H-bonding. Thus, this complementary participation of the formal electrophilic (Lewis acidic) phosphorus centre and the nucleophilic (Lewis basic) pyridyl-nitrogen centre resembles reported modes of H_2_O activation by intramolecular Frustrated Lewis Pairs (FLPs). Subsequently, an H-transfer process occurs to form the 1,2-dihydrophosphinine oxide in an overall slightly exergonic reaction. This step was identified to be favoured in the presence of an additional H_2_O molecule, thus forming a network to facilitate proton shuttling through an ordered six-membered transition state. This also rationalizes the observed *anti*-selectivity of the addition. The calculations are also in line with the fact, that neither the reference compound 2,3,5,6-tetraphenylphosphinine reacts with water, nor do alcohols react with the tetrapyridyl-functionalized phosphinines. Our here-presented results show yet another example of the fascinating reactivity of functionalized phosphinines towards small molecules, showcasing the intriguing properties of these aromatic, low-coordinate phosphorus compounds for the activation of E–H bonds. As a matter of fact, the re-aromatization of the heterocycle is a key aspect for maintaining a fully reversible reaction, also from a conceptual point of view. Despite the presence of multiple coordination sites, we could further show that a dimeric sandwich-type structure with the general composition [Cu_2_I_2_(phosphinine)]_2_ is formed selectively upon reaction of the starting material with CuI·SMe_2_.

## Data availability

All data can be found in the ESI.†

## Author contributions

C. M., N. T. C. and S. E. N.: conceptualization and supervision of the project, writing, reviewing and editing of the manuscript. S. M. R., M. J. E. and N. T. C. collected XRD data and solved and refined the crystal structures. N. T. C. and L. J. K. C. were involved in the validation of the XRD data. R. O. K., S. L. K., L. J. G. and N. T. C. contributed to the experimental work, formal analysis and data curation. S. E. N. performed the computational work.

## Conflicts of interest

There are no conflicts to declare.

## Supplementary Material

SC-015-D3SC05930H-s001

SC-015-D3SC05930H-s002
