# Supplementary material for: Highly selective, reversible water activation by P,N-cooperativity in pyridyl-functionalized phosphinines
Source: Chem Sci. 2024 Mar 14;15(15):5496–506. doi: 10.1039/d3sc05930h (PMC11023035; doi:10.1039/d3sc05930h)
Supplement: SC-015-D3SC05930H-s001 [file SC-015-D3SC05930H-s001.pdf]

Electronic Supplementary Material (ESI) for Chemical Science.
This journal is © The Royal Society of Chemistry 2024

Supporting Information

Highly selective, reversible water activation by P,N-cooperativity in pyridyl-functionalized phosphinines†

Richard O. Kopp,‡[a] Sabrina L. Kleynemeyer,‡[a] Lucie J. Groth,[a] Moritz J. Ernst,[a] Susanne M. Rupf,[a] Manuela Weber,[a] Laurence J. Kershaw Cook,[b] Nathan T. Coles,*[a,c] Samuel E. Neale,*[d] and Christian Müller*[a]

[a.] *M.Sc. R.O. Kopp, M.Sc. S. L. Kleynemeyer, M.Sc. L. J. Groth, M.Sc. M. J. Ernst, M. Weber, Dr. S. Rupf, Dr. N. T. Coles, Prof. Dr. C. Müller, Institute of Chemistry and Biochemistry, Freie Universität Berlin, Fabeckstr. 34/36, 14195 Berlin, Germany. E-mail: c.mueller@fu-berlin.de.*

[b.] *Dr. N. T. Coles, School of Chemistry, University of Nottingham, University Park, Nottingham NG7 2RD, United Kingdom. E-mail: Nathan.Coles@nottingham.ac.uk.*

[c.] *Dr. S. E. Neale, Department of Chemistry, University of Bath, Claverton Down, Bath BA2 7AY, United Kingdom. E-mail: sen36@bath.ac.uk.*

†. Dedicated to Prof. Dr. Hansjörg Grützmacher on the occasion of his 65th birthday.

‡ The authors have contributed equally.

Contents

Supporting Information 1

1. DFT Calculations 3

1.1 Computational Details 3

1.2 Free Energy Profiles and Structures 4

1.3 Compound Cartesian Coordinates (Å) and Energies (au) 7

2. Experimental Procedures 46

2.1 General Remarks 46

2.2 Synthetic Procedures 47

2.3 Oxidative Addition of Water 57

2.4 Deprotonation Studies 68

2.5 Coordination Chemistry of Cu(I) 74

3. UV/Vis Analysis 80

3.1 UV/Vis Details 80

4. Crystallographic Data 83

4.1 Crystallographic Details 83

5. References 89

1. DFT Calculations

1.1 Computational Details

DFT calculations were performed with Gaussian 16 (Revision C.01) at the TPSS/def2-TZVPP level[1,2] with the ultrafine integral grid option invoked. Frequency calculations carried out at the same level of theory yielded free energy corrections and confirmed local minima, which were confirmed with no imaginary frequencies. Saddle points were also confirmed via the identification of a single negative eigenvalue upon frequency analysis which constituted the relevant bond forming or breaking process. Transition states were perturbed along the forward and reverse directions of the imaginary frequency, and the resulting structures subsequently optimized to confirm and yield the respective reactants and products for a given transition state. Single point energy corrections were calculated at the M052X-D3(DCM)/def2-QZVPP level,[2–4] which was identified as a reliable functional in a benchmarking evaluation of main group kinetics, thermodynamics and non-covalent interactions by Grimme and coworkers.[5] Solvation corrections were carried out using the IEFPCM approximation[6] with CH_2Cl_2 as the solvent (where $\varepsilon = 8.93$). Empirical corrections for dispersion interactions were obtained with Grimme's standalone D3 program,[7] with the zero-damping function. NICS(0) and NICS(+1,–1) calculations were performed in ORCA 5.0.2 at the B3LYP/def2-TZVPP and PBE/def2-TZVPP levels of theory, where "NICS(+1)" corresponds to the ghost atom positioned at the *syn*-face with respect to the –OH and =O groups in $\mathbf{IV_{2b}}$ and $\mathbf{V_{2b}}$ respectively, while "NICS(–1)" corresponds to the *anti*-face.

1.2 Free Energy Profiles and Structures

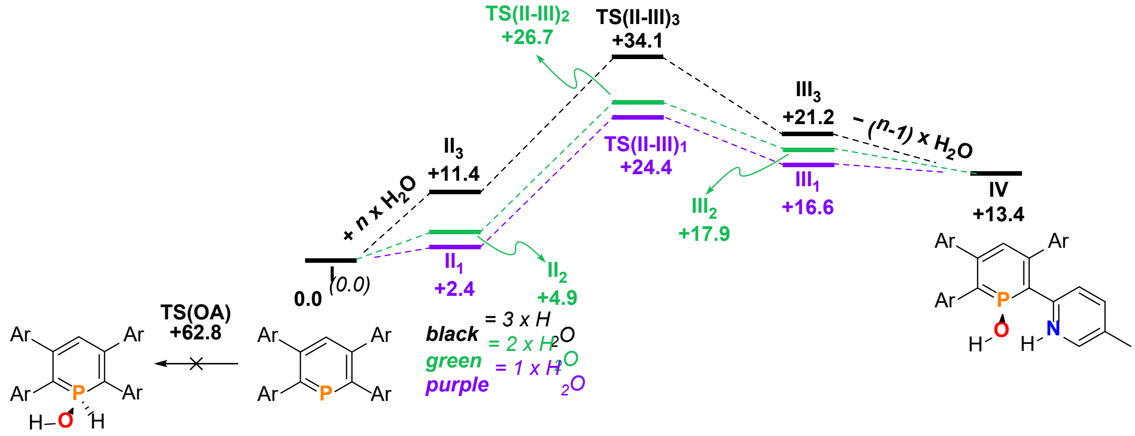

Figure S1.1. Free energy profiles of Addition of H_2O with no H_2O network (purple), with a second equivalent of H_2O (green) and a network of three H_2O molecules (black), along with energetics of kinetically disfavoured direct three-membered oxidative addition (left-hand side). Free energies quoted are in kcal/mol.

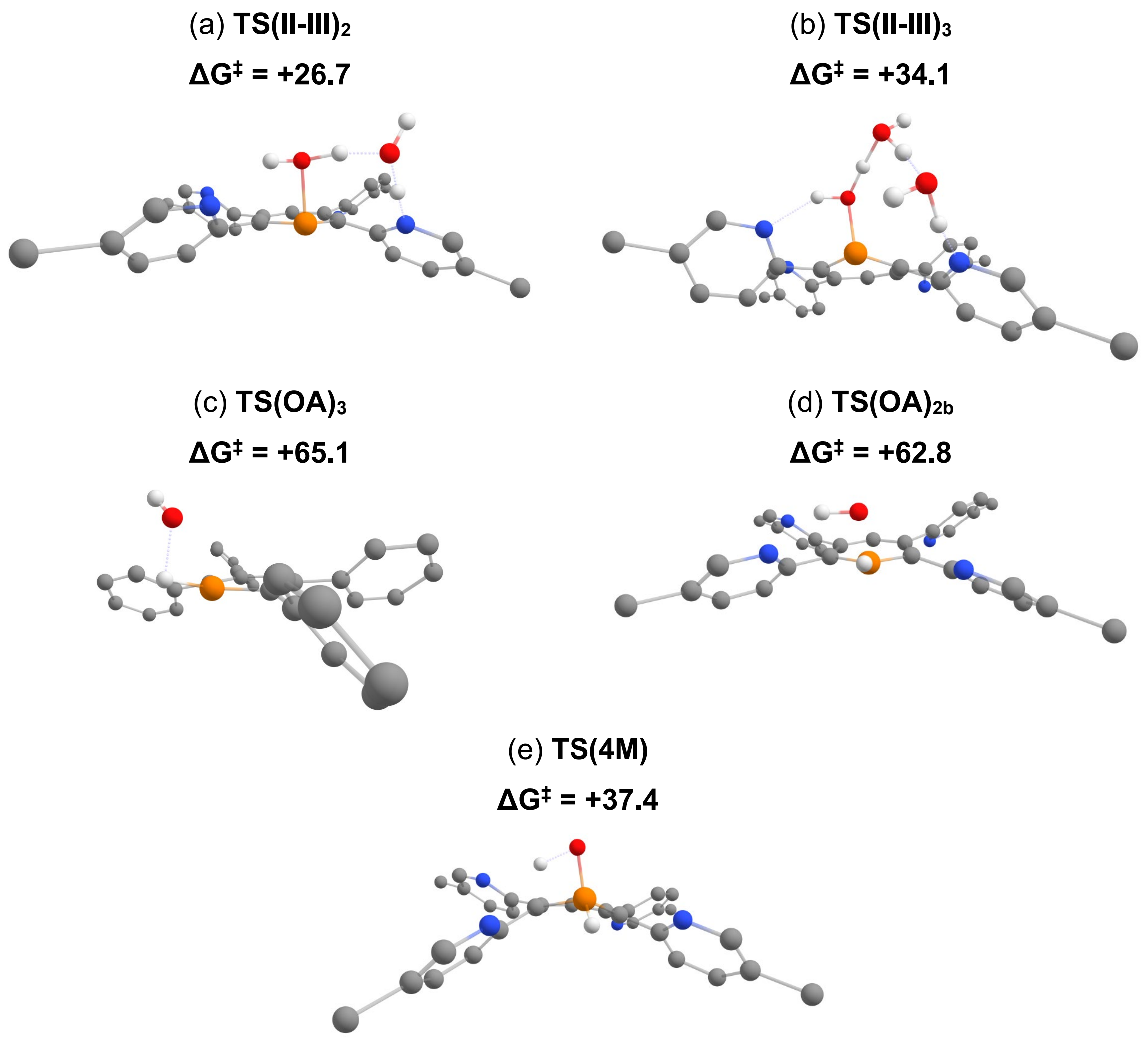

Figure S1.2. Structures of (a) transition states **TS(II-III)$_{2x}$**, (b) **TS(II-III)$_{3x}$** (c) **TS(OA)$_3$**, (d) **TS(OA)$_{2b}$** and (e) **TS(4M)**. Free energies quoted below the structures are in kcal mol^{-1}, and hydrogen atoms attached to carbons are omitted for clarity.

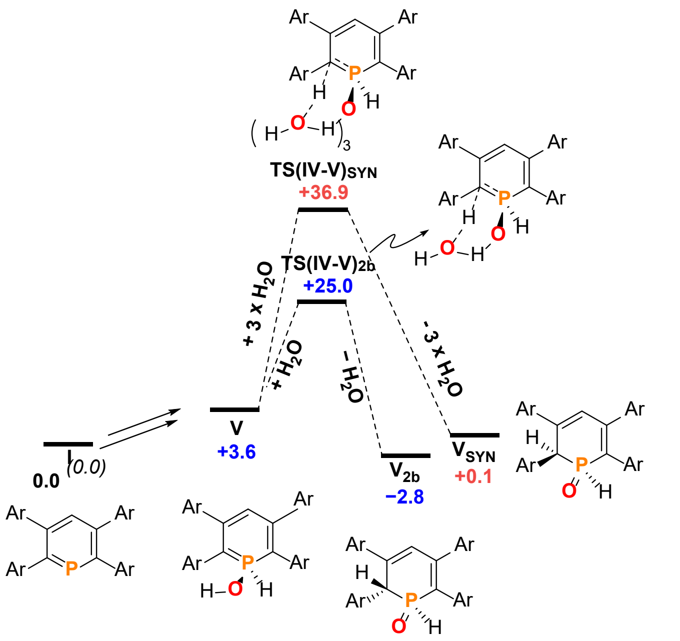

Figure S1.3. Free energy profiles of H+ transfer to form anti-addition product $\mathbf{V_{2b}}$ via **TS(IV-V)**$_{\mathbf{2b}}$, and syn-addition product $\mathbf{V_{SYN}}$ via **TS(IV-V)**$_{\mathbf{SYN}}$. Energies given in in kcal mol^{-1}.

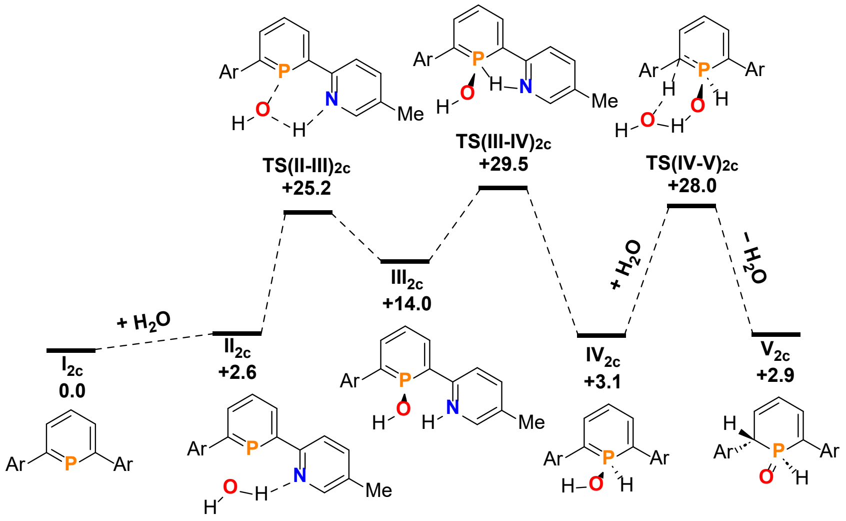

Figure S1.4. Free energy profile (calculated with DFT at the M052X-D3(CPCM=CH_2Cl_2)/def2-QZVPP//TPSS/def2-TZVPP level of theory, energies in kcal mol^{-1}) of H_2O addition at **2c** (2,6-(*p*-tolyl)pyridin-2-yl-phosphinine)

Table S1.1. NICS values at the B3LYP/def2-TZVPP level of theory for benzene (**C_6H_6**), **I_{2b}** and **V_{2b}**, in ppm. Note: NICS(+1) corresponds to the ghost atom positioned at the *syn*-face with respect to the –OH and =O groups in **IV_{2b}** and **V_{2b}** respectively, while NICS(–1) corresponds to the *anti*-face.

Entry	Structure	NICS(0)	NICS(+1)	NICS(– 1)
1	**C_6H_6**	-8.4	-10.0	-10.0
2	**I_{2b}**	-5.7	-7.3	-7.3
3	**IV_{2b}**	-2.9	-2.4	-3.9
4	**V_{2b}**	2.0	0.5	-0.3

Table S1.2. NICS values at the PBE/def2-TZVPP level of theory for benzene (**C_6H_6**), **I_{2b}** and **V_{2b}**, in ppm. Note: NICS(+1) corresponds to the ghost atom positioned at the *syn*-face with respect to the –OH and =O groups in **IV_{2b}** and **V_{2b}** respectively, while NICS(–1) corresponds to the *anti*-face.

Entry	Structure	NICS(0)	NICS(+1)	NICS(– 1)
1	**C_6H_6**	-7.9	-9.8	-9.8
2	**I_{2b}**	-5.2	-6.9	-7.0
3	**IV_{2b}**	-2.6	-2.2	-3.7
4	**V_{2b}**	1.9	0.2	-0.4

1.3 Compound Cartesian Coordinates (Å) and Energies (au)

MeOH
SCF (TPSS/def2-TZVP) Energy = -115.787750141
Enthalpy 0K = -115.737378
Enthalpy 298K = -115.733069
Free Energy 298K = -115.760174
Lowest Frequency = 297.7275 cm-1
Second Frequency = 1010.7323 cm-1
SCF (M052X-D3,DCM/def2-QZVPP) Energy = -115.766365986

C -0.04696 0.66959 -0.00000
H -1.09481 0.97621 -0.00000
H 0.43958 1.08147 0.89499
H 0.43958 1.08147 -0.89499
O -0.04696 -0.76197 0.00000
H 0.87306 -1.06096 0.00000

H_2O
SCF (TPSS/def2-TZVP) Energy = -76.4669886691

Enthalpy 0K = -76.446111
Enthalpy 298K = -76.442331
Free Energy 298K = -76.463774
Lowest Frequency = 1633.1505 cm-1
Second Frequency = 3713.2663 cm-1
SCF (M052X-D3,DCM/def2-QZVPP) Energy = -76.4629238628

O -0.00000 0.00000 0.11891
H -0.00000 0.76372 -0.47564
H -0.00000 -0.76372 -0.47564

I_{2b}
SCF (TPSS/def2-TZVP) Energy = -1681.26023332
Enthalpy 0K = -1680.800167
Enthalpy 298K = -1680.767700
Free Energy 298K = -1680.867279
Lowest Frequency = 15.3214 cm-1
Second Frequency = 28.4687 cm-1
SCF (M052X-D3,DCM/def2-QZVPP) Energy = -1681.01999812

P 0.00720 -1.89149 -0.10741
N -2.46316 2.58094 0.73183
N 2.81068 -2.51283 -0.84327
N -2.87180 -2.52269 -0.79681
N 3.36807 1.29720 -0.88667
C 1.36171 -0.77911 -0.03102
C 1.23506 0.62158 0.05786
C -0.01667 1.24837 0.08211
H -0.03831 2.33388 0.09543
C -1.25925 0.60496 0.02106
C -1.36406 -0.79781 -0.03355
C -2.67005 -1.50485 0.06195
C -3.60706 -1.19256 1.06306
H -3.40770 -0.38667 1.76218
C -2.44871 1.50439 -0.07653
C -3.46087 1.28477 -1.02506
H -3.40829 0.42727 -1.68767
C 2.67125 -1.48360 0.01234
C 3.60432 3.47173 0.84411
H 3.68975 4.31189 1.52999
C -4.00514 -3.22742 -0.68914
H -4.12414 -4.03784 -1.40861
C -5.00377 -2.99189 0.26101
C -4.77253 -1.94038 1.15795
H -5.50048 -1.71401 1.93416
C 4.59542 3.24381 -0.11747
C 2.42004 1.53275 0.03798
C -4.51028 2.18913 -1.11905
H -5.29332 2.03586 -1.85841
C 4.99590 -2.99483 0.08766
C 3.94142 -3.22680 -0.80030
H 4.00960 -4.04341 -1.51945
C 2.50942 2.61879 0.92363
H 1.73523 2.77934 1.66804
C 4.40897 2.13389 -0.95044
H 5.14767 1.90760 -1.71985
C 4.82269 -1.94111 0.99501
H 5.59417 -1.72080 1.72996
C -6.25165 -3.83579 0.31585
H -6.23371 -4.61138 -0.45501
H -7.15065 -3.22737 0.16285
H -6.35474 -4.32857 1.28945
C -3.49010 3.43450 0.63090
H -3.46275 4.28648 1.31078
C -4.55292 3.30092 -0.26814
C 3.66211 -1.18171 0.96184
H 3.51380 -0.37183 1.66880
C 6.24179 -3.84318 0.06875
H 6.15534 -4.64879 -0.66600
H 6.42984 -4.29649 1.04877
H 7.12501 -3.24637 -0.18830
C 5.80034 4.13857 -0.25963
H 5.50595 5.16095 -0.52364
H 6.47207 3.76948 -1.03975
H 6.36652 4.19498 0.67702

C -5.67167 4.30988 -0.31848
H -5.76156 4.75303 -1.31676
H -5.50218 5.11930 0.39728
H -6.63587 3.84544 -0.08129

II$_{2b}$
SCF (TPSS/def2-TZVP) Energy = -1757.73931044
Enthalpy 0K = -1757.255393
Enthalpy 298K = -1757.219519
Free Energy 298K = -1757.327839
Lowest Frequency = 14.4171 cm-1
Second Frequency = 21.6121 cm-1
SCF (M052X-D3,DCM/def2-QZVPP) Energy = -1757.49437518

P 0.01080 -1.73876 -0.16253
N 2.58115 2.71797 -0.67250
N -2.83158 -2.32987 0.57419
N 2.82743 -2.50725 0.51674
N -3.30493 1.43179 0.80267
C -1.32273 -0.60213 -0.15824
C -1.16852 0.79887 -0.15787
C 0.09653 1.39841 -0.13639
H 0.14065 2.48174 -0.07930
C 1.32646 0.72807 -0.10462
C 1.40565 -0.67634 -0.14278
C 2.69395 -1.41105 -0.25554
C 3.68044 -1.04622 -1.18839
H 3.53538 -0.17783 -1.82285
C 2.52997 1.59698 0.07077
C 3.51478 1.30270 1.02730
H 3.43119 0.40950 1.63753
C -2.64705 -1.27440 -0.24098
C -3.47545 3.74671 -0.74271
H -3.53574 4.64223 -1.35740
C 3.94215 -3.23831 0.39331
H 4.00565 -4.11160 1.04253
C 4.98719 -2.95430 -0.49171
C 4.82547 -1.82267 -1.30208
H 5.59168 -1.55560 -2.02670
C -4.48591 3.45761 0.18163
C -2.33682 1.72765 -0.08264
C 4.57706 2.18056 1.19809
H 5.33918 1.97031 1.94515
C -5.00584 -2.73272 -0.40869
C -3.97618 -3.02151 0.48900
H -4.06815 -3.85834 1.18000
C -2.39317 2.88446 -0.87647
H -1.60474 3.09295 -1.59354
C -4.33276 2.27843 0.92117
H -5.08755 2.00155 1.65749
C -4.79196 -1.64998 -1.27290
H -5.54514 -1.38535 -2.01199
C 6.21122 -3.83045 -0.56919
H 6.34826 -4.23146 -1.57990
H 6.13307 -4.67436 0.12188
H 7.11829 -3.26914 -0.31646
C 3.62032 3.54468 -0.49891
H 3.62377 4.43508 -1.12804
C 4.65899 3.33892 0.41483
C -3.61617 -0.91876 -1.19315
H -3.43674 -0.08688 -1.86625
C -6.26979 -3.55297 -0.44438
H -7.14489 -2.94279 -0.19184
H -6.21955 -4.38075 0.26824
H -6.44251 -3.97283 -1.44184
C -5.67729 4.35957 0.37996
H -5.36879 5.35239 0.72722
H -6.36538 3.94235 1.12045
H -6.22977 4.49974 -0.55607
C 5.79319 4.32240 0.54987
H 5.86668 4.70442 1.57435
H 5.65472 5.17521 -0.12065
H 6.75455 3.85465 0.30789
H -1.69514 -3.02280 2.01842
O -1.21341 -3.49376 2.73527

H -0.34038 -3.65461 2.34983

TS(II-III)$_{2b}$
SCF (TPSS/def2-TZVP) Energy = -1757.71652645
Enthalpy 0K = -1757.235333
Enthalpy 298K = -1757.201534
Free Energy 298K = -1757.302512
Lowest Frequency = -1039.8032 cm-1
Second Frequency = 17.3639 cm-1
SCF (M052X-D3,DCM/def2-QZVPP) Energy = -1757.46182129

P   0.03246 -1.78266 -0.16632
N   2.54952 2.74713 -0.63869
N -2.67641 -2.37400 0.71967
N   2.71635 -2.60379 0.30277
N -3.37574 1.40828 0.63691
C -1.34083 -0.63812 -0.21835
C -1.20529 0.75906 -0.26476
C   0.04556 1.39398 -0.22771
H   0.06642 2.47697 -0.17224
C   1.27975 0.73544 -0.14014
C   1.40724 -0.66478 -0.17917
C   2.68053 -1.38962 -0.29864
C   3.77218 -0.93960 -1.07079
H   3.71290 0.00982 -1.59206
C   2.45498 1.62705 0.10234
C   3.37391 1.34613 1.12767
H   3.25587 0.44811 1.72511
C -2.62664 -1.33350 -0.15824
C -3.51620 3.67001 -0.98778
H -3.56413 4.54423 -1.63371
C   3.81524 -3.35681 0.16698
H   3.78710 -4.32588 0.66695
C   4.95470 -2.98321 -0.54882
C   4.90219 -1.73354 -1.18572
H   5.74323 -1.39275 -1.78643
C -4.54561 3.41227 -0.07405
C -2.38523 1.67463 -0.23567
C   4.40938 2.23612 1.37590
H   5.11867 2.03492 2.17599
C -4.90952 -2.96463 0.08176
C -3.76390 -3.15653 0.83815
H -3.69335 -3.95463 1.57284
C -2.43218 2.80504 -1.07012
H -1.62751 2.98795 -1.77548
C -4.40716 2.25786 0.70429
H -5.17863 2.00422 1.43240
C -4.87486 -1.90207 -0.84445
H -5.73658 -1.72095 -1.48305
C   6.16173 -3.88201 -0.63667
H   6.41672 -4.10729 -1.67884
H   5.98150 -4.83009 -0.12122
H   7.04313 -3.41442 -0.18126
C   3.56393 3.58592 -0.38951
H   3.60499 4.47501 -1.01991
C   4.53409 3.39564 0.59901
C -3.75694 -1.09808 -0.96429
H -3.72670 -0.29640 -1.69261
C -6.11801 -3.85037 0.23962
H -6.99436 -3.27156 0.55317
H -5.94199 -4.62770 0.98820
H -6.37366 -4.34225 -0.70578
C -5.73894 4.32173 0.07308
H -5.43727 5.32017 0.41058
H -6.44927 3.92108 0.80201
H -6.26485 4.44658 -0.88036
C   5.64317 4.39369 0.81491
H   5.63166 4.78549 1.83854
H   5.54775 5.23970 0.12811
H   6.62659 3.93686 0.65330
H -1.59621 -2.49307 1.37382
O -0.40843 -2.31631 1.76437
H   0.06879 -3.15711 1.86403

III$_{2b}$

SCF (TPSS/def2-TZVP) Energy = -1757.73196196
Enthalpy 0K = -1757.245877
Enthalpy 298K = -1757.211606
Free Energy 298K = -1757.313948
Lowest Frequency = 17.3045 cm-1
Second Frequency = 21.0394 cm-1
SCF (M052X-D3,DCM/def2-QZVPP) Energy = -1757.48346127

P 0.00181 -1.58232 1.11702
H -1.75390 -3.09803 0.83135
O -0.02612 -1.09567 2.71833
N 2.26259 2.60788 -0.99607
N -2.54678 -2.77008 0.26679
N 2.86146 -2.40824 0.98393
N -3.52341 1.28364 0.95298
C -1.36656 -0.66968 0.34598
H -0.04064 -0.12612 2.79408
C -1.27718 0.75621 0.19249
C -0.04729 1.39444 0.17078
H -0.02634 2.47815 0.13162
C 1.22040 0.73614 0.14709
C 1.36953 -0.62272 0.39786
C 2.59544 -1.39294 0.13183
C 3.41643 -1.16022 -0.99079
H 3.16165 -0.37404 -1.69392
C 2.39029 1.64037 -0.06665
C 3.54182 1.54465 0.73418
H 3.60369 0.77668 1.49816
C -2.46262 -1.44033 -0.10967
C -3.63926 3.62268 -0.56089
H -3.67909 4.52491 -1.16789
C 3.94237 -3.16204 0.76444
H 4.11295 -3.96281 1.48547
C 4.83131 -2.99389 -0.30349
C 4.53081 -1.95933 -1.20045
H 5.16291 -1.79017 -2.07009
C -4.71712 3.28299 0.26665
C -2.48098 1.63207 0.16971
C 4.57861 2.44828 0.54938
H 5.46903 2.38815 1.17167
C -4.51611 -3.26423 -0.97391
C -3.49890 -3.64904 -0.13905
H -3.39474 -4.65789 0.24588
C -2.52127 2.80048 -0.61449
H -1.68734 3.04169 -1.26612
C -4.58849 2.09115 0.98913
H -5.40093 1.76863 1.64176
C -4.47865 -1.90776 -1.41213
H -5.23961 -1.55968 -2.10703
C 6.02782 -3.89331 -0.48125
H 6.06808 -4.65047 0.30737
H 6.96411 -3.32334 -0.44902
H 5.99789 -4.41241 -1.44654
C 3.27958 3.46317 -1.16392
H 3.13377 4.22294 -1.93277
C 4.47087 3.44247 -0.43225
C -3.50127 -1.03668 -1.00662
H -3.48211 -0.02660 -1.39180
C -5.59361 -4.21742 -1.41625
H -5.43010 -5.21730 -1.00466
H -5.62368 -4.30260 -2.50892
H -6.58339 -3.87653 -1.08982
C -5.95101 4.14170 0.37755
H -5.71527 5.12062 0.81159
H -6.70362 3.66573 1.01278
H -6.40106 4.32279 -0.60523
C 5.56615 4.44724 -0.68360
H 5.79402 5.02172 0.22167
H 5.27808 5.15288 -1.46819
H 6.49374 3.95396 -0.99680

TS(III-IV)$_{2b}$
SCF (TPSS/def2-TZVP) Energy = -1757.71320569
Enthalpy 0K = -1757.232629
Enthalpy 298K = -1757.198625

Free Energy 298K = -1757.300254
Lowest Frequency = -1266.9954 cm-1
Second Frequency = 17.8669 cm-1
SCF (M052X-D3,DCM/def2-QZVPP) Energy = -1757.45260395

P -0.02236 -1.49654 0.97843
H -0.99637 -2.77854 0.67285
O -0.00053 -1.42837 2.63306
N 2.29594 2.71644 -0.88734
N -2.31494 -2.76137 0.30667
N 2.64108 -2.54350 0.70567
N -3.53230 1.48210 0.90956
C -1.40962 -0.56819 0.36486
H 0.03216 -0.51154 2.95305
C -1.31063 0.83139 0.17889
C -0.05973 1.46073 0.14178
H -0.03253 2.54337 0.08290
C 1.19154 0.80402 0.12964
C 1.34914 -0.58168 0.32018
C 2.54079 -1.38054 0.01928
C 3.48990 -1.04974 -0.97065
H 3.37010 -0.14854 -1.56181
C 2.37452 1.70879 0.00322
C 3.48870 1.56660 0.84869
H 3.51363 0.76086 1.57516
C -2.50387 -1.44608 -0.01154
C -3.61935 3.65800 -0.83435
H -3.64763 4.49619 -1.52756
C 3.67620 -3.35118 0.46169
H 3.70251 -4.27294 1.04428
C 4.68738 -3.09138 -0.46966
C 4.55830 -1.90297 -1.20305
H 5.28878 -1.65508 -1.97071
C -4.69191 3.42777 0.03575
C -2.49766 1.72473 0.07859
C 4.53681 2.47132 0.75488
H 5.39828 2.37491 1.41234
C -4.35181 -3.47275 -0.72371
C -3.17345 -3.72974 -0.03614
H -2.89609 -4.73837 0.26349
C -2.51925 2.80954 -0.81608
H -1.68448 2.96733 -1.49174
C -4.57838 2.31457 0.87728
H -5.38650 2.08146 1.57179
C -4.56924 -2.12558 -1.08345
H -5.46019 -1.86399 -1.65062
C 5.83177 -4.04950 -0.67974
H 5.71010 -4.94478 -0.06289
H 6.79189 -3.58898 -0.41699
H 5.90077 -4.36702 -1.72676
C 3.32226 3.57329 -0.96630
H 3.21588 4.36761 -1.70603
C 4.47790 3.51215 -0.18139
C -3.67211 -1.12992 -0.74833
H -3.85267 -0.11073 -1.06066
C -5.32784 -4.56746 -1.06750
H -4.94279 -5.54574 -0.76568
H -5.52616 -4.60007 -2.14506
H -6.29042 -4.41627 -0.56427
C -5.90603 4.32055 0.07260
H -5.64174 5.33617 0.38996
H -6.65490 3.93488 0.77033
H -6.37209 4.40063 -0.91606
C 5.58750 4.52094 -0.33443
H 5.77237 5.05372 0.60546
H 5.34041 5.26199 -1.10005
H 6.52775 4.03702 -0.62343

IV_{2b}
SCF (TPSS/def2-TZVP) Energy = -1757.75120103
Enthalpy 0K = -1757.266802
Enthalpy 298K = -1757.232881
Free Energy 298K = -1757.334578
Lowest Frequency = 18.7780 cm-1
Second Frequency = 24.2247 cm-1

SCF (M052X-D3,DCM/def2-QZVPP) Energy = -1757.49757050

P 0.00954 -1.58764 0.45594
H -0.01856 -2.73435 -0.34264
O -0.08669 -2.15783 1.96874
N 2.50322 2.75561 -0.66341
N -2.63606 -2.50110 0.71195
N 2.56592 -2.70271 0.28606
N -3.40076 1.51592 0.72570
C -1.37956 -0.58917 0.06500
H -1.01054 -2.50989 2.01337
C -1.21339 0.79929 -0.05951
C 0.04881 1.42424 -0.03527
H 0.07546 2.50799 -0.03172
C 1.28506 0.77101 0.03093
C 1.42535 -0.63566 0.10188
C 2.61991 -1.43084 -0.17857
C 3.71664 -1.00100 -0.95701
H 3.72702 -0.00807 -1.39138
C 2.47467 1.66804 0.12961
C 3.47650 1.43047 1.08620
H 3.41166 0.55795 1.72807
C -2.61977 -1.36364 -0.03190
C -3.47155 3.67636 -1.03772
H -3.49209 4.51040 -1.73604
C 3.58507 -3.53064 0.03748
H 3.47782 -4.54125 0.43275
C 4.73552 -3.18071 -0.67435
C 4.76732 -1.87501 -1.18818
H 5.61417 -1.54867 -1.78870
C -4.53100 3.47961 -0.14424
C -2.38357 1.72389 -0.13151
C 4.52518 2.33087 1.21061
H 5.29938 2.16323 1.95630
C -4.82487 -3.07371 -0.14497
C -3.70074 -3.31314 0.64407
H -3.65078 -4.20883 1.26254
C -2.39160 2.80158 -1.03276
H -1.56257 2.93689 -1.72044
C -4.42579 2.37443 0.70881
H -5.22144 2.17115 1.42643
C -4.79224 -1.91052 -0.93173
H -5.62481 -1.68046 -1.59337
C 5.86176 -4.15927 -0.88765
H 5.59927 -5.14795 -0.49979
H 6.77700 -3.83250 -0.37900
H 6.10284 -4.26584 -1.95163
C 3.52919 3.60631 -0.53194
H 3.51363 4.46872 -1.19922
C 4.57869 3.45843 0.38046
C -3.69941 -1.06199 -0.88520
H -3.66128 -0.18018 -1.51419
C -5.99864 -4.01881 -0.15452
H -5.80166 -4.89519 0.46966
H -6.22007 -4.36866 -1.16941
H -6.90449 -3.53198 0.22607
C -5.72258 4.40159 -0.09214
H -5.42558 5.41710 0.19478
H -6.45945 4.04864 0.63498
H -6.21522 4.47106 -1.06864
C 5.69576 4.46699 0.46468
H 5.76007 4.90489 1.46740
H 5.54402 5.28058 -0.25043
H 6.66610 4.00445 0.24973

TS(IV-V)$_{2b}$
SCF (TPSS/def2-TZVP) Energy = -1834.20010048
Enthalpy 0K = -1833.696531
Enthalpy 298K = -1833.661054
Free Energy 298K = -1833.766388
Lowest Frequency = -1289.9068 cm-1
Second Frequency = 17.3324 cm-1
SCF (M052X-D3,DCM/def2-QZVPP) Energy = -1833.94026422

P 0.10409 -1.39220 1.18310

H 0.12381 -2.69171 0.66834
O 0.11803 -1.36399 2.71661
N 2.28397 2.71142 -1.00081
N -2.49508 -2.70202 0.34614
N 2.80141 -2.44736 0.83406
N -3.49108 1.34339 0.89177
C -1.37583 -0.59052 0.53792
H -2.03810 -0.53140 1.84816
C -1.22577 0.82420 0.22671
C -0.00342 1.46459 0.16982
H 0.00585 2.54554 0.07451
C 1.28072 0.83273 0.16559
C 1.46142 -0.52245 0.41784
C 2.64169 -1.32928 0.08940
C 3.51187 -1.04712 -0.98387
H 3.33567 -0.18474 -1.61746
C 2.43331 1.75240 -0.06705
C 3.58957 1.67381 0.72730
H 3.66411 0.91108 1.49540
C -2.32617 -1.47406 -0.18843
C -3.66490 3.51933 -0.84121
H -3.72577 4.36090 -1.52804
C 3.83157 -3.25542 0.56881
H 3.91051 -4.14082 1.20016
C 4.77360 -3.03759 -0.44254
C 4.57627 -1.89902 -1.23727
H 5.24925 -1.69052 -2.06648
C -4.75708 3.19824 -0.02452
C -2.44597 1.66294 0.10081
C 4.61255 2.59090 0.53064
H 5.50770 2.54678 1.14716
C -4.07970 -3.26573 -1.39818
C -3.33597 -3.55331 -0.24970
H -3.42655 -4.53253 0.22178
C -2.50762 2.75428 -0.78407
H -1.66200 2.97661 -1.42736
C -4.59640 2.09322 0.81859
H -5.41177 1.79602 1.47867
C -3.88121 -1.99635 -1.95713
H -4.40677 -1.71375 -2.86735
C 5.91800 -3.99076 -0.67153
H 5.84968 -4.85258 -0.00157
H 6.88379 -3.50245 -0.49415
H 5.92716 -4.36217 -1.70269
C 3.28795 3.57902 -1.18074
H 3.12735 4.33179 -1.95310
C 4.48412 3.57797 -0.45554
C -3.00105 -1.10099 -1.36509
H -2.82423 -0.12917 -1.81353
C -5.02843 -4.27308 -1.99676
H -4.97907 -5.22393 -1.45798
H -4.79296 -4.46842 -3.04939
H -6.06542 -3.91849 -1.95620
C -6.03952 3.98975 -0.04715
H -5.86502 5.03662 0.22696
H -6.76897 3.57412 0.65370
H -6.49036 3.98650 -1.04619
C 5.56308 4.59698 -0.71847
H 5.78137 5.18432 0.18075
H 5.26417 5.28931 -1.51059
H 6.49808 4.11436 -1.02567
H -1.15849 -0.96046 3.14110
O -2.19859 -0.65009 3.04594
H -2.30455 0.25879 3.37105

V_{2b}
SCF (TPSS/def2-TZVP) Energy = -1757.75250332
Enthalpy 0K = -1757.267507
Enthalpy 298K = -1757.233486
Free Energy 298K = -1757.337245
Lowest Frequency = 14.5418 cm-1
Second Frequency = 20.8625 cm-1
SCF (M052X-D3,DCM/def2-QZVPP) Energy = -1757.50646181

P 0.03507 -1.14111 1.85398

H 0.35094 -2.48933 1.60362
O -0.00657 -0.71725 3.28267
N 1.23719 2.68850 -1.34736
N -1.88671 -3.16097 0.08297
N 2.92368 -1.57896 1.67227
N -4.06838 0.23539 1.06827
C -1.54205 -0.91824 0.92771
H -2.34012 -1.20962 1.61942
C -1.73878 0.53083 0.55424
C -0.68593 1.35004 0.29009
H -0.87918 2.38374 0.02337
C 0.72402 0.98708 0.29442
C 1.18924 -0.20136 0.80448
C 2.54152 -0.75635 0.67081
C 3.36065 -0.56012 -0.45867
H 3.01674 0.05257 -1.28483
C 1.62694 2.05634 -0.22410
C 2.77592 2.44648 0.48198
H 3.04034 1.94160 1.40517
C -1.60797 -1.88836 -0.24651
C -4.82544 2.61284 -0.16009
H -5.11182 3.53694 -0.65748
C 4.12466 -2.15776 1.60503
H 4.38425 -2.80399 2.44364
C 5.02588 -1.99691 0.54556
C 4.59989 -1.18171 -0.51230
H 5.23623 -1.04332 -1.38386
C -5.79884 1.81764 0.46076
C -3.13383 1.01680 0.48934
C 3.54480 3.49484 -0.00448
H 4.42964 3.81718 0.53978
C -1.74352 -3.79231 -2.25363
C -1.95078 -4.06896 -0.89775
H -2.18376 -5.08647 -0.58298
C -3.49684 2.21726 -0.15319
H -2.75146 2.82089 -0.66021
C -5.34329 0.63299 1.04848
H -6.05302 -0.03687 1.53432
C -1.43923 -2.46531 -2.57525
H -1.25901 -2.18104 -3.60993
C 6.36524 -2.68690 0.54079
H 6.47997 -3.32552 1.42105
H 7.18576 -1.95954 0.54266
H 6.48799 -3.31202 -0.35102
C 2.00285 3.68657 -1.80744
H 1.65665 4.16207 -2.72530
C 3.17237 4.14256 -1.19000
C -1.36716 -1.50509 -1.57107
H -1.13252 -0.47221 -1.80771
C -1.84581 -4.87291 -3.30034
H -2.04401 -5.84534 -2.84058
H -0.91923 -4.95461 -3.87981
H -2.65571 -4.66400 -4.00897
C -7.25438 2.20397 0.49147
H -7.40497 3.13444 1.05150
H -7.85691 1.42427 0.96565
H -7.64359 2.36727 -0.51997
C 3.97081 5.28340 -1.76652
H 4.04342 6.11451 -1.05588
H 3.51010 5.66116 -2.68354
H 4.99363 4.97007 -2.00512

TS(IV-V)$_{2b,SYN}$
SCF (TPSS/def2-TZVP) Energy = -1987.15948242
Enthalpy 0K = -1986.606424
Enthalpy 298K = -1986.566402
Free Energy 298K = -1986.681379
Lowest Frequency = -957.3415 cm-1
Second Frequency = 17.9030 cm-1
SCF (M052X-D3,DCM/def2-QZVPP) Energy = -1986.88525638

P 0.19077 -1.51121 0.09483
H 0.31384 -2.25839 -1.09731
O 0.02332 -2.46861 1.25192
N 2.70714 3.03079 -0.28336

N -3.07765 -1.98942 0.30141
N 2.84337 -2.45777 0.01549
N -3.09751 1.34020 -1.40739
C -1.22563 -0.41070 -0.12928
C -1.00479 1.02099 -0.22572
C 0.22436 1.63074 -0.11957
H 0.29780 2.70455 -0.26002
C 1.47314 0.96383 0.08296
C 1.63055 -0.41186 0.00701
C 2.88578 -1.12886 -0.23529
C 4.02577 -0.55607 -0.84437
H 4.03167 0.48825 -1.13377
C 2.61269 1.87719 0.40282
C 3.48726 1.57686 1.46099
H 3.36657 0.64938 2.01115
C -2.45038 -0.83376 0.61635
C -3.38850 3.96170 -0.50503
H -3.49690 4.97969 -0.13701
C 3.91286 -3.20827 -0.25751
H 3.80826 -4.27144 -0.03893
C 5.10678 -2.72171 -0.79848
C 5.12737 -1.35378 -1.11016
H 6.00498 -0.91684 -1.58256
C -4.34915 3.41990 -1.36830
C -2.17167 1.87605 -0.57924
C 4.48449 2.48212 1.79448
H 5.16107 2.26577 2.61838
C -4.70424 -1.67230 2.06493
C -4.15129 -2.38396 1.00440
H -4.59374 -3.32881 0.69034
C -2.30271 3.19390 -0.10817
H -1.56439 3.59639 0.57775
C -4.13652 2.09960 -1.77638
H -4.85325 1.62535 -2.44749
C -4.05585 -0.47481 2.40066
H -4.41636 0.12444 3.23430
C 6.28535 -3.62482 -1.05406
H 6.02583 -4.66995 -0.86196
H 7.13338 -3.36581 -0.40814
H 6.63185 -3.54553 -2.09087
C 3.68595 3.88374 0.04601
H 3.73147 4.79896 -0.54494
C 4.61342 3.67609 1.07181
C -2.94024 -0.06372 1.69054
H -2.41594 0.84215 1.97441
C -5.91576 -2.17360 2.80789
H -6.26076 -3.12719 2.39809
H -6.74502 -1.45943 2.74535
H -5.69466 -2.32338 3.87095
C -5.54924 4.20358 -1.83381
H -6.15231 4.54766 -0.98581
H -6.18928 3.59496 -2.47883
H -5.24702 5.09243 -2.39958
C 5.68160 4.69283 1.38334
H 5.60448 5.55866 0.71960
H 6.68356 4.26423 1.26562
H 5.60063 5.04931 2.41663
H -0.18343 -3.99308 0.42379
O -0.41219 -4.72729 -0.21816
H -0.83445 -5.40844 0.32294
H -1.52144 -3.97741 -1.39353
O -2.27814 -3.44211 -1.76476
H -1.96904 -2.29932 -2.35816
H -2.58644 -0.72955 -2.79786
O -1.75559 -1.21009 -2.62833
H -1.48781 -0.79280 -1.59553
H -2.63982 -2.97387 -0.92075

$V_{2b,SYN}$
SCF (TPSS/def2-TZVP) Energy = -1757.74831537
Enthalpy 0K = -1757.263643
Enthalpy 298K = -1757.229678
Free Energy 298K = -1757.332573
Lowest Frequency = 12.9112 cm-1
Second Frequency = 26.2705 cm-1

SCF (M052X-D3,DCM/def2-QZVPP) Energy = -1757.50231364

P 0.02812 -1.67292 0.43567
H 0.12521 -2.83301 -0.35635
O -0.19076 -1.88663 1.89578
N 2.45159 2.73520 -0.57712
N -3.08133 -2.24823 -1.06449
N 2.75261 -2.68070 0.33005
N -3.33146 1.22775 -1.09417
C -1.30386 -0.73191 -0.43311
C -1.17396 0.75141 -0.14785
C 0.04124 1.32979 0.05221
H 0.08410 2.41143 0.13562
C 1.34095 0.67737 0.05472
C 1.49402 -0.68809 0.01538
C 2.73030 -1.44451 -0.21499
C 3.78265 -0.99473 -1.03689
H 3.72473 -0.02579 -1.52052
C 2.49576 1.61687 0.16985
C 3.52899 1.38456 1.09189
H 3.51450 0.49105 1.70724
C -2.62407 -1.40684 -0.12448
C -3.64270 3.56628 0.38936
H -3.76379 4.46688 0.98743
C 3.82342 -3.45228 0.12875
H 3.79119 -4.43550 0.59824
C 4.93460 -3.08345 -0.63877
C 4.87977 -1.81875 -1.24144
H 5.69355 -1.48471 -1.88156
C -4.63269 3.19311 -0.52866
C -2.37498 1.61006 -0.22564
C 4.54585 2.32133 1.21702
H 5.34547 2.16151 1.93686
C -4.96877 -2.78736 0.35930
C -4.22108 -2.90694 -0.81542
H -4.55929 -3.57805 -1.60539
C -2.51114 2.77770 0.54680
H -1.75017 3.04449 1.27323
C -4.40859 2.00495 -1.23471
H -5.14911 1.65790 -1.95571
C -4.45422 -1.93037 1.34049
H -4.97206 -1.81345 2.29006
C 6.10684 -4.01191 -0.82250
H 5.92848 -4.97070 -0.32774
H 7.02341 -3.58096 -0.40242
H 6.29717 -4.20737 -1.88395
C 3.45103 3.61749 -0.45225
H 3.38044 4.50224 -1.08514
C 4.53362 3.47553 0.42254
C -3.27287 -1.24001 1.10608
H -2.84396 -0.59475 1.86566
C -6.25127 -3.55586 0.55587
H -6.44879 -4.21216 -0.29674
H -7.10758 -2.87993 0.66575
H -6.20876 -4.17619 1.45837
C -5.88064 4.01034 -0.74192
H -6.44522 4.12432 0.19060
H -6.53650 3.53880 -1.47911
H -5.63817 5.01766 -1.10002
C 5.61374 4.52278 0.51134
H 5.41164 5.35395 -0.16993
H 6.59324 4.10387 0.25356
H 5.69081 4.92706 1.52702
H -1.10769 -0.89938 -1.50415

II_{2x}
SCF (TPSS/def2-TZVP) Energy = -1834.22167115
Enthalpy 0K = -1833.712655
Enthalpy 298K = -1833.674148
Free Energy 298K = -1833.788239
Lowest Frequency = 15.4053 cm-1
Second Frequency = 20.0817 cm-1
SCF (M052X-D3,DCM/def2-QZVPP) Energy = -1833.97216285

P -0.01540 -1.57469 -0.37011

N 2.45260 2.96182 -0.61196
N -2.80700 -2.29332 0.32076
N 2.87326 -2.28789 0.28402
N -3.37503 1.46613 0.83395
C -1.37109 -0.46714 -0.28681
C -1.24428 0.93293 -0.19414
C 0.00819 1.55720 -0.13971
H 0.02921 2.63571 -0.01676
C 1.25191 0.91294 -0.15438
C 1.35714 -0.48571 -0.27793
C 2.66343 -1.17596 -0.44924
C 3.60415 -0.75329 -1.40384
H 3.40594 0.12969 -2.00240
C 2.43995 1.79436 0.05820
C 3.45135 1.46199 0.97352
H 3.40085 0.53066 1.52783
C -2.67781 -1.16744 -0.40536
C -3.62471 3.85499 -0.58311
H -3.71590 4.78028 -1.14784
C 4.00595 -2.98239 0.09846
H 4.11839 -3.86535 0.72578
C 4.99928 -2.63860 -0.82078
C 4.76739 -1.48759 -1.58642
H 5.49474 -1.17235 -2.33126
C -4.61156 3.49399 0.34129
C -2.43101 1.83072 -0.05136
C 4.49869 2.35050 1.17955
H 5.28072 2.10965 1.89613
C -4.98720 -2.68864 -0.64939
C -3.92824 -3.01610 0.20101
H -3.97367 -3.90948 0.82219
C -2.52596 3.02624 -0.78089
H -1.75481 3.29072 -1.49833
C -4.41888 2.28153 1.01510
H -5.15346 1.94904 1.74874
C -4.83030 -1.53004 -1.42224
H -5.60883 -1.23128 -2.12096
C 6.24432 -3.47279 -0.97954
H 6.33134 -3.86099 -2.00069
H 6.23615 -4.32407 -0.29366
H 7.14534 -2.88235 -0.77786
C 3.47705 3.79888 -0.40586
H 3.44807 4.72766 -0.97598
C 4.53939 3.55838 0.47188
C -3.67842 -0.76564 -1.30446
H -3.54359 0.12552 -1.90860
C -6.22308 -3.54753 -0.72967
H -6.12678 -4.43171 -0.09384
H -6.40621 -3.88453 -1.75629
H -7.11171 -2.99338 -0.40529
C -5.81933 4.35598 0.60678
H -5.52705 5.33724 0.99806
H -6.48312 3.88571 1.33755
H -6.39287 4.52950 -0.31078
C 5.65485 4.55656 0.64795
H 5.74194 4.87296 1.69347
H 5.48387 5.44755 0.03735
H 6.62051 4.12729 0.35693
H -1.74064 -3.16315 1.80935
O -1.42364 -3.74752 2.52760
H 1.32234 -3.07189 3.13210
O 1.52174 -3.68825 2.41286
H 1.86076 -3.11890 1.68040
H -0.48928 -3.92022 2.30352

TS(II-III)$_{2x}$
SCF (TPSS/def2-TZVP) Energy = -1834.19962026
Enthalpy 0K = -1833.695571
Enthalpy 298K = -1833.659963
Free Energy 298K = -1833.764585
Lowest Frequency = -1222.6630 cm-1
Second Frequency = 18.1541 cm-1
SCF (M052X-D3,DCM/def2-QZVPP) Energy = -1833.93887975

P -0.03311 -1.67330 0.04437

N 2.37318 2.93327 -0.49758
N -2.75580 -2.38950 0.67898
N 2.90328 -2.39523 0.14540
N -3.50301 1.48616 0.67590
C -1.43123 -0.56556 -0.10628
C -1.31178 0.81937 -0.15781
C -0.06419 1.48852 -0.08468
H -0.07604 2.57098 -0.01551
C 1.17761 0.86924 -0.02476
C 1.33780 -0.54511 -0.06400
C 2.60401 -1.17463 -0.39337
C 3.51689 -0.63730 -1.33449
H 3.28719 0.31748 -1.79325
C 2.34267 1.77641 0.19338
C 3.33441 1.46897 1.14176
H 3.26928 0.54470 1.70673
C -2.70007 -1.31815 -0.15014
C -3.65107 3.65421 -1.07375
H -3.70037 4.49281 -1.76528
C 3.99923 -3.08392 -0.23811
H 4.12687 -4.04917 0.24674
C 4.91519 -2.61362 -1.16236
C 4.64402 -1.33970 -1.70749
H 5.31691 -0.91796 -2.45095
C -4.69337 3.42602 -0.16779
C -2.50543 1.72033 -0.19671
C 4.36833 2.36704 1.36669
H 5.13133 2.14287 2.10934
C -4.95200 -2.96257 -0.16083
C -3.84649 -3.16833 0.66461
H -3.83417 -4.01120 1.35525
C -2.55049 2.80550 -1.08777
H -1.73197 2.96776 -1.78249
C -4.54828 2.32055 0.67857
H -5.32718 2.09608 1.40828
C -4.87823 -1.86707 -1.03462
H -5.69593 -1.66550 -1.72366
C 6.12452 -3.41816 -1.56187
H 6.11316 -3.64327 -2.63476
H 6.16263 -4.36715 -1.01978
H 7.05218 -2.87204 -1.35464
C 3.38338 3.78172 -0.26943
H 3.36659 4.70106 -0.85613
C 4.42034 3.56615 0.64396
C -3.76009 -1.04860 -1.03375
H -3.68838 -0.21296 -1.72134
C -6.14890 -3.87774 -0.11707
H -5.99158 -4.69523 0.59250
H -6.34998 -4.31768 -1.10073
H -7.05253 -3.33665 0.18783
C -5.90711 4.31748 -0.09476
H -5.63103 5.34223 0.17991
H -6.61865 3.94960 0.65007
H -6.42286 4.36784 -1.06054
C 5.52175 4.57696 0.83782
H 5.57064 4.91668 1.87879
H 5.36412 5.45402 0.20351
H 6.50122 4.15198 0.58874
H -1.14319 -2.39438 1.88923
O -0.24211 -1.98557 1.94029
H 2.21148 -3.17666 2.93012
O 1.71637 -3.39984 2.12860
H 2.27926 -2.89309 1.15260
H 0.64175 -2.75130 2.13424

III_{2x}
SCF (TPSS/def2-TZVP) Energy = -1834.21612466
Enthalpy 0K = -1833.705330
Enthalpy 298K = -1833.668727
Free Energy 298K = -1833.775839
Lowest Frequency = 16.6297 cm-1
Second Frequency = 23.5788 cm-1
SCF (M052X-D3,DCM/def2-QZVPP) Energy = -1833.95819811

P -0.02960 -1.68382 0.27576

N 2.39268 2.96180 -0.29633
N -2.81202 -2.28150 0.88521
N 2.86915 -2.45695 -0.07933
N -3.51249 1.54908 0.56018
C -1.42391 -0.55272 0.00424
C -1.28921 0.81559 -0.12724
C -0.03036 1.48540 -0.02545
H -0.04511 2.56873 0.03520
C 1.20129 0.87237 0.07262
C 1.36218 -0.55654 0.04395
C 2.55826 -1.15730 -0.43327
C 3.46787 -0.56304 -1.35767
H 3.26981 0.45051 -1.68398
C 2.36396 1.76854 0.33203
C 3.36538 1.40896 1.25270
H 3.30300 0.45489 1.76603
C -2.70213 -1.29242 -0.03341
C -3.54212 3.62321 -1.30545
H -3.54389 4.42578 -2.04017
C 3.89740 -3.17087 -0.61198
H 3.98824 -4.18638 -0.24129
C 4.75981 -2.62746 -1.53007
C 4.51910 -1.26957 -1.88629
H 5.16549 -0.79154 -2.61876
C -4.63451 3.45114 -0.44734
C -2.46791 1.72640 -0.26988
C 4.40235 2.29246 1.51701
H 5.17189 2.02672 2.23901
C -4.97713 -2.89983 -0.00133
C -3.91443 -3.04239 0.89216
H -3.94904 -3.81754 1.65742
C -2.45212 2.76567 -1.21529
H -1.59450 2.88644 -1.87026
C -4.54702 2.39108 0.46249
H -5.36567 2.21297 1.16083
C -4.84364 -1.89294 -0.96883
H -5.62494 -1.74601 -1.71187
C 5.89524 -3.41802 -2.12370
H 5.79475 -3.49767 -3.21268
H 5.92747 -4.43143 -1.71423
H 6.86078 -2.93969 -1.92126
C 3.40487 3.79567 -0.02949
H 3.38493 4.74550 -0.56555
C 4.44969 3.53001 0.86180
C -3.71264 -1.09167 -0.98926
H -3.59433 -0.32251 -1.74484
C -6.19044 -3.79146 0.07008
H -6.07988 -4.54112 0.85896
H -6.35376 -4.31857 -0.87703
H -7.09762 -3.21267 0.28060
C -5.84006 4.35573 -0.48637
H -5.56671 5.39184 -0.25502
H -6.59281 4.03607 0.23998
H -6.30532 4.35647 -1.47878
C 5.55453 4.52739 1.10017
H 5.61450 4.80907 2.15780
H 5.39172 5.43844 0.51707
H 6.53106 4.11637 0.81801
H -1.12921 -2.18503 2.06487
O -0.18727 -1.89153 1.99903
H 2.31564 -3.44148 3.02036
O 1.79645 -3.67419 2.23761
H 2.35110 -2.90645 0.70154
H 0.97590 -3.10604 2.29890

II_{3x}
SCF (TPSS/def2-TZVP) Energy = -1910.70014158
Enthalpy 0K = -1910.166654
Enthalpy 298K = -1910.125464
Free Energy 298K = -1910.246018
Lowest Frequency = 15.1738 cm-1
Second Frequency = 17.5807 cm-1
SCF (M052X-D3,DCM/def2-QZVPP) Energy = -1910.44215719

P 0.00014 -1.43647 -0.47070

N	2.37538	3.16926	-0.40119
N	-2.80990	-2.15366	0.37876
N	2.92466	-2.15787	-0.21177
N	-3.51675	1.47881	0.62876
C	-1.38629	-0.37290	-0.37388
C	-1.30306	1.02612	-0.26529
C	-0.06740	1.67846	-0.14470
H	-0.07632	2.74878	0.03657
C	1.18927	1.06673	-0.16432
C	1.34472	-0.31704	-0.39477
C	2.66969	-0.91907	-0.68493
C	3.60031	-0.26950	-1.51794
H	3.36336	0.70454	-1.93180
C	2.34270	1.95371	0.17571
C	3.29781	1.56425	1.12875
H	3.22351	0.59188	1.60473
C	-2.67496	-1.11337	-0.46435
C	-3.70682	3.91753	-0.70949
H	-3.77402	4.86249	-1.24429
C	4.09095	-2.74472	-0.52165
H	4.22918	-3.74590	-0.11587
C	5.08372	-2.16474	-1.31295
C	4.80106	-0.89128	-1.82529
H	5.51713	-0.39244	-2.47466
C	-4.75050	3.50606	0.12737
C	-2.51614	1.89254	-0.16831
C	4.31151	2.44846	1.47226
H	5.04923	2.16424	2.21936
C	-4.95566	-2.68685	-0.60606
C	-3.92104	-2.89944	0.30713
H	-3.98413	-3.71853	1.02262
C	-2.58232	3.11412	-0.85824
H	-1.76711	3.41811	-1.50798
C	-4.58548	2.27018	0.76455
H	-5.36551	1.89718	1.42864
C	-4.78951	-1.61795	-1.49811
H	-5.55073	-1.41006	-2.24707
C	6.37526	-2.88375	-1.60566
H	6.36974	-3.89004	-1.17808
H	7.23253	-2.34352	-1.18714
H	6.54278	-2.97326	-2.68479
C	3.36781	4.00232	-0.06159
H	3.35860	4.97262	-0.55868
C	4.37411	3.70904	0.86434
C	-3.65181	-0.82735	-1.43070
H	-3.50885	-0.00128	-2.11990
C	-6.17760	-3.56926	-0.62847
H	-6.09951	-4.36541	0.11710
H	-6.31255	-4.03618	-1.61080
H	-7.08573	-2.99391	-0.41396
C	-5.98791	4.34011	0.34036
H	-5.74208	5.30055	0.80806
H	-6.70179	3.82343	0.98795
H	-6.48781	4.55971	-0.60981
C	5.45421	4.70755	1.19357
H	5.45402	4.95336	2.26155
H	5.31426	5.63590	0.63263
H	6.44749	4.31226	0.95146
H	-1.49756	-2.09700	1.95153
O	-0.76944	-1.91603	2.58004
H	1.01584	-4.32920	3.97225
O	0.36137	-4.47262	3.27507
H	0.88357	-4.49064	2.43334
H	-0.46063	-2.80086	2.87042
H	1.91033	-3.53136	0.55908
O	1.58289	-4.43636	0.80348
H	0.80865	-4.55364	0.23348

TS(II-III)$_{3x}$
SCF (TPSS/def2-TZVP) Energy = -1910.67571597
Enthalpy 0K = -1910.149352
Enthalpy 298K = -1910.111460
Free Energy 298K = -1910.221810
Lowest Frequency = -1284.2247 cm-1
Second Frequency = 17.8568 cm-1

SCF (M052X-D3,DCM/def2-QZVPP) Energy = -1910.40568572

P	-0.04829	-1.55304	0.10763
N	2.30508	3.11660	-0.11662
N	-2.87403	-2.13735	0.78036
N	2.91526	-2.26889	-0.41659
N	-3.60652	1.54703	0.47426
C	-1.45020	-0.45489	-0.12934
C	-1.33692	0.92744	-0.17237
C	-0.09541	1.59850	-0.00188
H	-0.11738	2.67395	0.13947
C	1.14336	0.98344	0.05360
C	1.30999	-0.43276	-0.09931
C	2.53568	-0.97673	-0.65199
C	3.35305	-0.21635	-1.53337
H	3.04550	0.79148	-1.78510
C	2.29793	1.86632	0.38822
C	3.30937	1.43104	1.26366
H	3.26484	0.42854	1.67669
C	-2.72743	-1.19982	-0.18493
C	-3.60515	3.78001	-1.19726
H	-3.59479	4.64506	-1.85715
C	4.01819	-2.79057	-0.99495
H	4.21223	-3.82983	-0.73796
C	4.86166	-2.08898	-1.83808
C	4.48605	-0.75544	-2.10327
H	5.08110	-0.15127	-2.78496
C	-4.72950	3.50913	-0.40852
C	-2.53004	1.82075	-0.28615
C	4.33617	2.29808	1.60913
H	5.11553	1.97179	2.29482
C	-5.00483	-2.80106	-0.15235
C	-3.97793	-2.89780	0.78724
H	-4.04348	-3.62920	1.59220
C	-2.49979	2.94031	-1.13511
H	-1.61866	3.13578	-1.73858
C	-4.65655	2.37259	0.40488
H	-5.50080	2.11460	1.04540
C	-4.83465	-1.84384	-1.16391
H	-5.58984	-1.73336	-1.93950
C	6.08981	-2.71925	-2.44098
H	6.18698	-3.76406	-2.13258
H	7.00054	-2.19118	-2.13420
H	6.05341	-2.69270	-3.53646
C	3.30691	3.93455	0.22717
H	3.26974	4.93328	-0.20993
C	4.36135	3.59566	1.08147
C	-3.70165	-1.04514	-1.18427
H	-3.55416	-0.31033	-1.96870
C	-6.22222	-3.68741	-0.08092
H	-6.14712	-4.39009	0.75396
H	-6.34630	-4.26845	-1.00204
H	-7.13593	-3.09734	0.05682
C	-5.95385	4.38879	-0.42174
H	-5.71547	5.40608	-0.09005
H	-6.72839	3.99159	0.24063
H	-6.37715	4.46677	-1.42977
C	5.45335	4.58034	1.41356
H	5.51197	4.75987	2.49333
H	5.27716	5.54099	0.92096
H	6.43448	4.21127	1.09230
H	-1.31076	-1.93622	1.92555
O	-0.34473	-1.69460	1.97023
H	1.55293	-3.15098	3.65206
O	0.92724	-3.49454	2.99904
H	1.50730	-3.86240	2.02671
H	0.28233	-2.59022	2.53816
H	2.33219	-3.19366	0.32654
O	2.00485	-4.17679	0.93096
H	1.27861	-4.55994	0.41580

III_{3x}
SCF (TPSS/def2-TZVP) Energy = -1910.69950371
Enthalpy 0K = -1910.164158
Enthalpy 298K = -1910.124606

Free Energy 298K = -1910.239161
Lowest Frequency = 16.2923 cm-1
Second Frequency = 19.5566 cm-1
SCF (M052X-D3,DCM/def2-QZVPP) Energy = -1910.43272731

P -0.04459 -1.52553 0.28285
N 2.30629 3.16089 -0.07809
N -2.82031 -2.16375 0.81988
N 2.78984 -2.29146 -0.56544
N -3.59177 1.62161 0.47346
C -1.45109 -0.42800 -0.04981
C -1.33079 0.94454 -0.14517
C -0.08159 1.62333 0.01920
H -0.11128 2.70051 0.14807
C 1.15605 1.02138 0.08290
C 1.32337 -0.40714 -0.04676
C 2.47753 -0.95115 -0.67302
C 3.36407 -0.21370 -1.51885
H 3.15290 0.83462 -1.68619
C 2.30876 1.90460 0.41383
C 3.33492 1.46234 1.26904
H 3.29776 0.45547 1.67215
C -2.71528 -1.18896 -0.11318
C -3.57097 3.76574 -1.31132
H -3.55256 4.59627 -2.01398
C 3.81849 -2.89863 -1.21260
H 3.89816 -3.96400 -1.02408
C 4.68374 -2.20511 -2.02160
C 4.41859 -0.81435 -2.15917
H 5.05029 -0.21615 -2.81214
C -4.69683 3.54639 -0.50909
C -2.51498 1.84434 -0.30285
C 4.36380 2.32902 1.60997
H 5.15366 1.99745 2.28097
C -4.95904 -2.83454 -0.09236
C -3.90988 -2.94275 0.82282
H -3.94558 -3.70410 1.60185
C -2.47345 2.91966 -1.20627
H -1.58994 3.07713 -1.81752
C -4.63329 2.45321 0.36289
H -5.47841 2.23804 1.01787
C -4.82806 -1.84095 -1.07360
H -5.60030 -1.71966 -1.83058
C 5.83127 -2.87673 -2.72772
H 5.85760 -3.94726 -2.50678
H 6.79243 -2.44525 -2.42386
H 5.75181 -2.75818 -3.81485
C 3.30960 3.97787 0.26192
H 3.26364 4.98126 -0.16352
C 4.37720 3.63291 1.09798
C -3.71170 -1.01838 -1.08798
H -3.59605 -0.25521 -1.85021
C -6.15653 -3.74794 -0.02734
H -6.04937 -4.47758 0.78051
H -6.28884 -4.29907 -0.96558
H -7.07882 -3.18225 0.15016
C -5.91273 4.43590 -0.56656
H -5.66398 5.46674 -0.28876
H -6.68979 4.08126 0.11663
H -6.33701 4.46517 -1.57675
C 5.47010 4.61769 1.42649
H 5.54118 4.78648 2.50723
H 5.28481 5.58266 0.94591
H 6.44848 4.25521 1.08972
H -1.11085 -1.84477 2.13494
O -0.17064 -1.57684 2.01702
H 2.03059 -3.02023 3.81583
O 1.30260 -3.43381 3.33240
H 1.61086 -4.20093 1.78069
H 0.77871 -2.68135 2.94146
H 2.21487 -2.92789 0.02667
O 1.66201 -4.43955 0.81733
H 0.74601 -4.34437 0.50807

TS(4M)

SCF (TPSS/def2-TZVP) Energy = -1757.69731931
Enthalpy 0K = -1757.217857
Enthalpy 298K = -1757.184179
Free Energy 298K = -1757.286015
Lowest Frequency = -1669.0738 cm-1
Second Frequency = 17.4557 cm-1
SCF (M052X-D3,DCM/def2-QZVPP) Energy = -1757.43851480

P 0.05630 -1.45592 1.07282
H 0.13289 -2.78797 0.66028
O -0.27472 -1.25437 2.57963
N 2.26922 2.66565 -0.94449
N -2.59147 -2.72640 0.49968
N 2.78318 -2.50796 0.86945
N -3.52203 1.32067 0.95520
C -1.44233 -0.63330 0.51641
H -1.35562 -0.75333 1.95402
C -1.27727 0.77759 0.23838
C -0.03395 1.39024 0.16421
H -0.01483 2.47192 0.08381
C 1.24674 0.76466 0.16422
C 1.43990 -0.59880 0.40127
C 2.63207 -1.40054 0.10678
C 3.52569 -1.12002 -0.94630
H 3.35927 -0.26249 -1.58917
C 2.40152 1.69253 -0.02349
C 3.54129 1.60138 0.79224
H 3.60230 0.82692 1.54968
C -2.52582 -1.49049 -0.03596
C -3.64164 3.60197 -0.64367
H -3.68271 4.48204 -1.28195
C 3.82273 -3.31411 0.63254
H 3.89342 -4.19226 1.27462
C 4.78660 -3.09823 -0.35802
C 4.60233 -1.96630 -1.16540
H 5.29591 -1.75828 -1.97746
C -4.72771 3.27712 0.17799
C -2.47610 1.65441 0.17300
C 4.56560 2.52454 0.63256
H 5.44770 2.47210 1.26691
C -4.46050 -3.27071 -0.94041
C -3.52688 -3.57137 0.05690
H -3.53278 -4.55609 0.52504
C -2.51236 2.79283 -0.65158
H -1.67251 3.02213 -1.29989
C -4.59668 2.11427 0.94743
H -5.41362 1.80614 1.60064
C -4.36014 -1.99729 -1.51397
H -5.03545 -1.70605 -2.31599
C 5.94431 -4.04373 -0.54931
H 5.86299 -4.90315 0.12222
H 6.90080 -3.54741 -0.34602
H 5.98601 -4.41926 -1.57808
C 3.27396 3.53872 -1.08882
H 3.12738 4.30380 -1.85169
C 4.45475 3.52818 -0.33858
C -3.39172 -1.10526 -1.07157
H -3.30089 -0.12644 -1.52888
C -5.50207 -4.27123 -1.37224
H -5.34662 -5.23527 -0.87926
H -5.47349 -4.43531 -2.45544
H -6.51241 -3.92601 -1.12214
C -5.97375 4.12280 0.23806
H -5.75631 5.12040 0.63751
H -6.72978 3.66145 0.87965
H -6.41020 4.25985 -0.75777
C 5.53581 4.55456 -0.56081
H 5.73167 5.12762 0.35263
H 5.25234 5.25889 -1.34797
H 6.47907 4.07983 -0.85467

TS(OA)$_{2b}$
SCF (TPSS/def2-TZVP) Energy = -1757.66271231
Enthalpy 0K = -1757.182778
Enthalpy 298K = -1757.148477

Free Energy 298K = -1757.251175
Lowest Frequency = -1419.0524 cm-1
Second Frequency = 17.3315 cm-1
SCF (M052X-D3,DCM/def2-QZVPP) Energy = -1757.39827371

P 0.03142 -1.68460 0.16159
H 0.00737 -3.09009 0.72247
O -0.12885 -2.47457 1.97431
N 2.51467 2.73838 -0.66400
N -2.78197 -2.40933 0.78098
N 2.71156 -2.65471 0.31454
N -3.37555 1.43561 0.73654
C -1.36651 -0.64794 -0.01600
H -1.11839 -2.51244 1.98715
C -1.20529 0.74443 -0.11712
C 0.05013 1.37754 -0.10033
H 0.06702 2.46171 -0.08353
C 1.29111 0.73621 -0.03897
C 1.43689 -0.66902 -0.01799
C 2.68516 -1.41792 -0.23026
C 3.75210 -0.95811 -1.02812
H 3.69560 0.00893 -1.51529
C 2.47058 1.64120 0.11402
C 3.44096 1.39855 1.10049
H 3.36247 0.51855 1.73049
C -2.65539 -1.36901 -0.07252
C -3.51246 3.61449 -0.99970
H -3.55874 4.45624 -1.68739
C 3.78674 -3.42421 0.11615
H 3.74974 -4.41204 0.57613
C 4.91168 -3.04221 -0.62048
C 4.86106 -1.77081 -1.21061
H 5.68666 -1.42444 -1.82896
C -4.54570 3.39821 -0.08049
C -2.38503 1.66173 -0.14575
C 4.47744 2.30597 1.27005
H 5.22748 2.13571 2.03936
C -4.97329 -2.90992 -0.11670
C -3.90457 -3.14115 0.74988
H -3.95041 -3.96388 1.46273
C -2.42532 2.74914 -1.03381
H -1.61659 2.90033 -1.74219
C -4.40811 2.28499 0.75733
H -5.18155 2.06706 1.49445
C -4.82145 -1.84611 -1.01880
H -5.60604 -1.62900 -1.74058
C 6.09916 -3.95688 -0.77826
H 5.90438 -4.93266 -0.32393
H 6.99238 -3.53656 -0.30047
H 6.34157 -4.11689 -1.83510
C 3.52961 3.59502 -0.48961
H 3.52872 4.46534 -1.14655
C 4.54959 3.44336 0.45492
C -3.66788 -1.07831 -1.00349
H -3.53509 -0.26742 -1.71142
C -6.21250 -3.76721 -0.08566
H -6.11098 -4.57712 0.64228
H -6.41073 -4.21520 -1.06606
H -7.09588 -3.17794 0.18724
C -5.74335 4.30885 0.01383
H -5.44694 5.32482 0.29950
H -6.45518 3.94378 0.75958
H -6.26512 4.38083 -0.94721
C 5.65564 4.45883 0.58774
H 5.68538 4.88289 1.59793
H 5.51998 5.28116 -0.12051
H 6.63555 4.00615 0.39673

I$_C$
SCF (TPSS/def2-TZVP) Energy = -1244.60424510
Enthalpy 0K = -1244.295153
Enthalpy 298K = -1244.274913
Free Energy 298K = -1244.344917
Lowest Frequency = 37.0166 cm-1
Second Frequency = 42.4472 cm-1

SCF (M052X-D3,DCM/def2-QZVPP) Energy = -1244.43573848

P -0.02290 -1.84942 0.06283
C 2.96278 -2.49877 -0.73535
N -2.83409 -2.55999 -0.44080
C 1.33295 -0.73002 0.00233
C 1.20358 0.66159 -0.03062
C -0.01440 1.35733 -0.02669
C -1.23659 0.66829 0.01676
C -1.37053 -0.71910 0.04812
C -2.72267 -1.32927 0.10225
C -3.81924 -0.68588 0.70173
H -3.69785 0.28440 1.17255
C 2.69358 -1.32584 -0.00852
C -4.02747 -3.16119 -0.41669
H -4.06718 -4.15149 -0.86716
C -5.17218 -2.58673 0.13684
C -5.05640 -1.32210 0.71181
H -5.91519 -0.84144 1.17197
C 5.27158 -2.46763 -0.01483
C 4.23703 -3.06242 -0.73867
H 4.42374 -3.96429 -1.31481
C 5.01938 -1.30458 0.71510
H 5.81489 -0.84006 1.29121
C 3.74552 -0.74051 0.71885
H 3.55355 0.14811 1.31383
H 6.26509 -2.90651 -0.01832
H -6.11857 -3.11822 0.12277
H 2.16551 -2.95627 -1.31478
H 2.11470 1.25173 -0.11747
H -2.14371 1.27045 0.01714
C -0.01339 2.84005 -0.09254
C -0.97957 3.53218 -0.84306
C 0.95441 3.59257 0.59497
C -0.97774 4.92434 -0.90395
C 0.95448 4.98470 0.53678
C -0.01145 5.65746 -0.21339
H -1.71968 2.97187 -1.40750
H 1.69636 3.08091 1.20151
H -1.72704 5.43717 -1.50042
H 1.70591 5.54563 1.08535
H -0.01039 6.74249 -0.26023

II$_C$
SCF (TPSS/def2-TZVP) Energy = -1321.08218339
Enthalpy 0K = -1320.749143
Enthalpy 298K = -1320.725540
Free Energy 298K = -1320.803863
Lowest Frequency = 20.2771 cm-1
Second Frequency = 36.2976 cm-1
SCF (M052X-D3,DCM/def2-QZVPP) Energy = -1320.90924308

P -0.71792 -1.41655 -0.30941
N -3.56423 -0.66503 0.19537
C 1.52827 -3.46550 0.53636
C -1.35787 0.21721 -0.21696
C -0.57339 1.36297 -0.08838
C 0.82692 1.37021 0.00831
C 1.55709 0.17229 -0.02026
C 1.00255 -1.10458 -0.14302
C 1.89835 -2.28853 -0.13784
C 3.13208 -2.26693 -0.81216
H 3.42413 -1.37758 -1.36385
C -2.83211 0.34768 -0.31855
C 2.36444 -4.58021 0.53786
H 2.06405 -5.47588 1.07404
C 3.58691 -4.54362 -0.13456
C 3.96668 -3.38234 -0.81031
H 4.91187 -3.34792 -1.34491
C -5.58444 0.46459 -0.45938
C -4.89964 -0.60122 0.12102
H -5.43262 -1.44401 0.55561
C -4.83522 1.50805 -1.00114
H -5.32492 2.35225 -1.47816
C -3.44706 1.44965 -0.93610

H -2.84199 2.23370 -1.37934
H -3.13328 -2.26732 1.25048
O -3.16059 -3.10700 1.76194
H -2.78715 -3.75466 1.14819
H 0.58739 -3.49095 1.07994
H -1.07800 2.32700 -0.04994
H 2.63499 0.24189 0.11772
C 1.54143 2.66086 0.16992
C 1.00383 3.69298 0.95808
C 2.77616 2.88471 -0.46303
C 1.67662 4.90408 1.10775
C 3.44707 4.09701 -0.31637
C 2.90062 5.11240 0.47019
H 0.06599 3.53250 1.48251
H 3.19946 2.11058 -1.09687
H 1.24841 5.68299 1.73227
H 4.39497 4.25172 -0.82395
H 3.42449 6.05658 0.58640
H -6.66915 0.47038 -0.48850
H 4.23882 -5.41231 -0.13210

TS(II-III)$_C$
SCF (TPSS/def2-TZVP) Energy = -1321.05314041
Enthalpy 0K = -1320.723331
Enthalpy 298K = -1320.701548
Free Energy 298K = -1320.774169
Lowest Frequency = -915.2542 cm-1
Second Frequency = 37.9542 cm-1
SCF (M052X-D3,DCM/def2-QZVPP) Energy = -1320.87402844

P -0.20908 -1.69247 -0.31848
N -3.09569 -1.91548 0.33796
C 2.62169 -2.79831 0.51261
C -1.39608 -0.35836 -0.29018
C -1.07248 0.99795 -0.19134
C 0.21876 1.50903 -0.03456
C 1.31799 0.62829 0.01900
C 1.28351 -0.75891 -0.09627
C 2.54771 -1.52798 -0.08791
C 3.71394 -1.02617 -0.69853
H 3.67879 -0.06408 -1.20208
C -2.79354 -0.75416 -0.31742
C 3.80489 -3.53543 0.50384
H 3.83403 -4.51079 0.98209
C 4.95123 -3.01882 -0.10025
C 4.89824 -1.75803 -0.69979
H 5.78107 -1.34922 -1.18411
C -5.40442 -1.74848 -0.21069
C -4.34588 -2.40475 0.38945
H -4.47100 -3.33149 0.94093
C -5.13812 -0.54273 -0.88047
H -5.94434 -0.00537 -1.37136
C -3.84603 -0.04838 -0.93541
H -3.62115 0.86236 -1.47904
H -2.13693 -2.34292 0.96098
O -0.94378 -2.47328 1.49846
H -0.69588 -3.41138 1.45019
H 1.74712 -3.19231 1.02279
H -1.89492 1.71283 -0.20834
H 2.29720 1.07934 0.17944
C 0.42916 2.97002 0.10048
C -0.49600 3.78200 0.78193
C 1.56074 3.59566 -0.45438
C -0.30323 5.15758 0.89642
C 1.75724 4.96988 -0.33553
C 0.82528 5.76083 0.33907
H -1.36133 3.32382 1.25335
H 2.28197 2.99967 -1.00683
H -1.03029 5.75812 1.43665
H 2.63690 5.42638 -0.78137
H 0.97824 6.83211 0.43164
H -6.40558 -2.15941 -0.15603
H 5.87463 -3.59038 -0.10549

III$_C$

SCF (TPSS/def2-TZVP) Energy = -1321.07297877
Enthalpy 0K = -1320.738286
Enthalpy 298K = -1320.716043
Free Energy 298K = -1320.790035
Lowest Frequency = 27.3168 cm-1
Second Frequency = 41.9278 cm-1
SCF (M052X-D3,DCM/def2-QZVPP) Energy = -1320.89783338

P   0.09630 1.68720 0.49711
N   2.87767 2.44910 -0.10880
C -2.92618 2.53776 0.55076
C   1.42878 0.52176 0.08669
C   1.22090 -0.88174 0.06160
C -0.02029 -1.49293 0.05521
C -1.22162 -0.71234 -0.02465
C -1.33232 0.65374 0.06893
C -2.63498 1.32930 -0.11160
C -3.62600 0.79488 -0.95885
H -3.41490 -0.11074 -1.52059
C   2.70371 1.07280 -0.18660
C -4.15644 3.17107 0.38855
H -4.35599 4.09809 0.91936
C -5.13170 2.61791 -0.44295
C -4.85713 1.42588 -1.11705
H -5.59994 0.99385 -1.78248
C   5.16425 2.42077 -0.71461
C   4.03277 3.10870 -0.36934
H   3.98749 4.18762 -0.27610
C   5.06801 1.00625 -0.80951
H   5.94287 0.42917 -1.09455
C   3.88445 0.35790 -0.56288
H   3.82476 -0.71784 -0.66814
H   2.02196 2.97353 0.10882
O   0.06589 1.71134 2.18375
H -0.09224 0.82537 2.55207
H -2.18474 2.97065 1.21722
H   2.08854 -1.53733 0.00375
H -2.14695 -1.27499 -0.14666
C -0.11424 -2.97149 0.06083
C   0.79725 -3.74953 0.79978
C -1.10370 -3.65277 -0.67292
C   0.73246 -5.14145 0.79485
C -1.17282 -5.04446 -0.67378
C -0.25436 -5.79902 0.05832
H   1.54783 -3.25072 1.40732
H -1.81079 -3.08724 -1.27309
H   1.44545 -5.71411 1.38215
H -1.94265 -5.54202 -1.25757
H -0.31028 -6.88367 0.05894
H   6.08969 2.94717 -0.90921
H -6.09021 3.11266 -0.57080

TS(III-IV)$_C$
SCF (TPSS/def2-TZVP) Energy = -1321.05160050
Enthalpy 0K = -1320.722364
Enthalpy 298K = -1320.700544
Free Energy 298K = -1320.773313
Lowest Frequency = -1295.4450 cm-1
Second Frequency = 32.0392 cm-1
SCF (M052X-D3,DCM/def2-QZVPP) Energy = -1320.86545055

P   1.34038 0.75828 0.57693
N   3.53641 -0.67696 -0.10155
C   0.43903 3.77321 0.47858
C   1.19574 -0.95656 0.17777
C -0.03965 -1.61116 0.09355
C -1.26531 -0.93964 0.09019
C -1.31073 0.48044 0.03694
C -0.25878 1.38543 0.12306
C -0.44692 2.83463 -0.08620
C -1.50659 3.33173 -0.87108
H -2.18226 2.63648 -1.36071
C   2.47924 -1.54195 -0.14923
C   0.26697 5.14113 0.28039
H   0.96482 5.83980 0.73381

C -0.79928 5.61496 -0.48569
C -1.68395 4.69959 -1.06023
H -2.50811 5.05238 -1.67450
C 5.08122 -2.34111 -0.80355
C 4.78086 -1.03997 -0.43095
H 5.53777 -0.26209 -0.37613
C 4.02295 -3.26489 -0.85847
H 4.21733 -4.29157 -1.15602
C 2.73210 -2.87590 -0.54854
H 1.91231 -3.58207 -0.61692
H 2.90243 0.54059 0.11973
O 1.54208 0.98222 2.21466
H 0.74772 0.72393 2.71249
H 1.26069 3.42457 1.09987
H -0.06067 -2.69182 -0.04418
H -2.30241 0.91793 -0.07319
C -2.52708 -1.71527 0.05478
C -2.64061 -2.93248 0.75296
C -3.64791 -1.28088 -0.67783
C -3.81345 -3.68439 0.71361
C -4.82388 -2.02721 -0.71037
C -4.91484 -3.23561 -0.01673
H -1.80417 -3.27696 1.35550
H -3.58867 -0.36110 -1.25307
H -3.87196 -4.61678 1.26896
H -5.66952 -1.66904 -1.29148
H -5.83166 -3.81723 -0.04332
H 6.09984 -2.62640 -1.03967
H -0.93531 6.68144 -0.63931

IV$_C$
SCF (TPSS/def2-TZVP) Energy = -1321.08910993
Enthalpy 0K = -1320.756100
Enthalpy 298K = -1320.734249
Free Energy 298K = -1320.807311
Lowest Frequency = 32.8960 cm-1
Second Frequency = 37.7628 cm-1
SCF (M052X-D3,DCM/def2-QZVPP) Energy = -1320.91027844

P 0.41746 1.49863 0.13189
N 3.19383 1.81968 0.13738
C -2.31833 3.06463 0.49262
C 1.50862 0.16068 -0.09769
C 1.02074 -1.14977 -0.07282
C -0.32490 -1.52097 0.03030
C -1.33924 -0.53984 0.06799
C -1.19657 0.84836 0.06291
C -2.35482 1.75928 -0.03682
C -3.53306 1.35485 -0.69715
H -3.57835 0.37146 -1.15581
C 2.91094 0.53531 -0.22253
C -3.41192 3.92095 0.37200
H -3.35620 4.92017 0.79508
C -4.57546 3.49730 -0.26958
C -4.62815 2.20679 -0.80223
H -5.52191 1.86772 -1.31897
C 5.51224 1.46618 -0.40021
C 4.45540 2.25952 0.03410
H 4.62107 3.29490 0.32514
C 5.23029 0.14603 -0.76731
H 6.02213 -0.50606 -1.12531
C 3.92758 -0.32259 -0.68959
H 3.68565 -1.33208 -1.00504
H 0.59915 2.49413 -0.84348
O 0.70964 2.33870 1.49687
H 1.66776 2.56306 1.43156
H -1.43762 3.40127 1.03189
H 1.75360 -1.95143 -0.14688
H -2.36355 -0.90473 0.12038
C -0.68863 -2.95762 0.08763
C 0.12051 -3.88375 0.77113
C -1.84782 -3.44690 -0.54211
C -0.20860 -5.23742 0.81574
C -2.18278 -4.79841 -0.49025
C -1.36394 -5.70390 0.18695

H 1.00352 -3.53170 1.29802
H -2.48084 -2.76391 -1.10220
H 0.43229 -5.92751 1.35802
H -3.08092 -5.14766 -0.99258
H -1.62410 -6.75761 0.22583
H 6.51864 1.86764 -0.45093
H -5.42876 4.16316 -0.35819

TS(IV-V)$_C$
SCF (TPSS/def2-TZVP) Energy = -1397.53307554
Enthalpy 0K = -1397.181088
Enthalpy 298K = -1397.157779
Free Energy 298K = -1397.234342
Lowest Frequency = -1381.5218 cm-1
Second Frequency = 25.5162 cm-1
SCF (M052X-D3,DCM/def2-QZVPP) Energy = -1397.34770043

P -0.33724 -1.51082 0.44859
N -3.13480 -1.81052 -0.18954
C 2.43922 -3.01577 0.47624
C -1.48409 -0.13828 0.22590
C -0.94609 1.19389 0.07576
C 0.38547 1.53859 0.06435
C 1.41676 0.53502 0.00708
C 1.26936 -0.82778 0.07251
C 2.39662 -1.75565 -0.15226
C 3.45205 -1.41548 -1.02163
H 3.42012 -0.47007 -1.55580
C -2.78508 -0.52057 -0.38363
C 3.50171 -3.89027 0.25348
H 3.51514 -4.85376 0.75525
C 4.54735 -3.53018 -0.59685
C 4.51619 -2.28684 -1.23348
H 5.31590 -2.00287 -1.91208
C -5.19986 -1.42336 -1.36141
C -4.30480 -2.24074 -0.67352
H -4.53257 -3.29032 -0.49635
C -4.83427 -0.09340 -1.57507
H -5.48915 0.57749 -2.12442
C -3.61380 0.36661 -1.09333
H -3.30079 1.38853 -1.28026
H -0.58766 -2.50804 -0.50566
O -0.43770 -2.12450 1.85857
H 1.64896 -3.29395 1.16836
H -1.67286 2.00114 -0.00610
H 2.43402 0.90853 -0.10364
C 0.77648 2.97097 0.03594
C 0.05150 3.92948 0.76654
C 1.86834 3.42196 -0.72770
C 0.39211 5.28037 0.72532
C 2.21416 4.77137 -0.76452
C 1.47739 5.70968 -0.03977
H -0.77546 3.60466 1.39263
H 2.43761 2.71335 -1.32281
H -0.18289 5.99723 1.30519
H 3.05723 5.09214 -1.37031
H 1.74862 6.76089 -0.06745
H -6.14303 -1.81831 -1.72473
H 5.37483 -4.21237 -0.76859
H -1.30698 -1.35976 2.56696
O -2.02851 -0.53996 2.78811
H -1.57805 0.09389 3.37029
H -1.87141 -0.15440 1.62426

V$_C$
SCF (TPSS/def2-TZVP) Energy = -1321.08074065
Enthalpy 0K = -1320.747148
Enthalpy 298K = -1320.725286
Free Energy 298K = -1320.799668
Lowest Frequency = 22.3661 cm-1
Second Frequency = 27.2403 cm-1
SCF (M052X-D3,DCM/def2-QZVPP) Energy = -1320.90972950

P -1.30845 0.39248 1.47329
N -1.42236 3.55642 0.50047

C -2.95870 -2.21625 0.74482
C -0.02567 1.68623 1.14799
C 1.32424 1.08876 0.87957
C 1.54042 -0.13301 0.33262
C 0.45044 -1.03245 -0.04916
C -0.85510 -0.92407 0.31341
C -1.88674 -1.89087 -0.10790
C -1.83196 -2.50101 -1.37517
H -1.03564 -2.23183 -2.06368
C -0.50843 2.66447 0.07867
C -3.92876 -3.13379 0.34703
H -4.74179 -3.38009 1.02391
C -3.85543 -3.73997 -0.90811
C -2.80303 -3.41791 -1.76809
H -2.74616 -3.87278 -2.75310
C -1.50031 4.47661 -1.72472
C -1.89838 4.43739 -0.38833
H -2.63213 5.14477 -0.00653
C -0.55968 3.54427 -2.15668
H -0.22144 3.53490 -3.18898
C -0.05386 2.62136 -1.24326
H 0.68509 1.88451 -1.54047
H -2.46951 0.95681 0.90018
O -1.45114 -0.03501 2.89637
H -3.00583 -1.77030 1.73524
H 2.18077 1.71837 1.11026
H 0.74416 -1.90765 -0.62748
C 2.93214 -0.60168 0.08606
C 3.94074 -0.37377 1.03638
C 3.27642 -1.26963 -1.10074
C 5.25124 -0.78543 0.80186
C 4.58819 -1.67785 -1.33736
C 5.58144 -1.43765 -0.38718
H 3.68444 0.10679 1.97627
H 2.51877 -1.45101 -1.85835
H 6.01323 -0.60796 1.55546
H 4.83447 -2.18244 -2.26734
H 6.60170 -1.76273 -0.56824
H -1.91697 5.21587 -2.40159
H -4.61482 -4.45225 -1.21719
H -0.00125 2.25230 2.08787

II_H
SCF (TPSS/def2-TZVP) Energy = -1797.05907854
Enthalpy 0K = -1796.546783
Enthalpy 298K = -1796.509430
Free Energy 298K = -1796.622652
Lowest Frequency = 13.0239 cm-1
Second Frequency = 16.2154 cm-1
SCF (M052X-D3,DCM/def2-QZVPP) Energy = -1796.79813566

P 0.02083 -1.59254 -0.35513
N 2.70472 2.82558 -0.54121
N -2.88869 -2.11535 0.33677
N 2.83363 -2.45892 0.28773
N -3.24101 1.57972 0.75932
C -1.28248 -0.42390 -0.28867
C -1.09535 0.97010 -0.19473
C 0.18309 1.53541 -0.11845
H 0.25328 2.61091 0.01255
C 1.39540 0.83325 -0.12269
C 1.44082 -0.56676 -0.25691
C 2.71494 -1.31792 -0.41824
C 3.70300 -0.91449 -1.33352
H 3.56965 -0.00728 -1.91404
C 2.61924 1.65637 0.12017
C 3.58939 1.26917 1.05842
H 3.47842 0.33730 1.60290
C -2.62529 -1.04775 -0.43944
C -3.32224 4.00679 -0.61071
H -3.34778 4.94678 -1.15773
C 3.93515 -3.19989 0.11482
H 3.98690 -4.11072 0.71142
C 4.98021 -2.88204 -0.75860
C 4.83412 -1.70178 -1.49967

H   5.60141 -1.40499 -2.21149
C -4.36205 3.67300 0.26471
C -2.24404 1.91831 -0.07729
C   4.67301 2.10413 1.29655
H   5.42433 1.82123 2.03047
C -5.03314 -2.39757 -0.75138
C -4.05898 -2.75231 0.18336
H -4.21885 -3.59479 0.85480
C -2.25558 3.13213 -0.78319
H -1.44532 3.37526 -1.46408
C -4.25344 2.43843 0.91652
H -5.03279 2.12443 1.61114
C -4.73617 -1.30275 -1.57460
H -5.44393 -0.98569 -2.33759
C   6.18819 -3.77308 -0.89583
H   6.30955 -4.12008 -1.92833
H   6.10122 -4.65250 -0.25163
H   7.10710 -3.24208 -0.62133
C   3.76390 3.61006 -0.30397
H   3.79493 4.54251 -0.86810
C   4.79053 3.31249 0.59798
C -3.53591 -0.62501 -1.42157
H -3.29191 0.21806 -2.05976
C -6.32715 -3.16205 -0.86525
H -7.18816 -2.51649 -0.65637
H -6.35218 -3.99637 -0.15901
H -6.46121 -3.56709 -1.87482
C -5.53834 4.58527 0.50203
H -5.21823 5.53888 0.93759
H -6.25758 4.12693 1.18650
H -6.05903 4.81427 -0.43469
C   5.94916 4.25343 0.80816
H   6.02569 4.55973 1.85755
H   5.83759 5.15505 0.19931
H   6.89956 3.77863 0.53822
H -2.10129 -2.92141 1.93114
O -1.98950 -3.46310 2.74398
C -0.85316 -4.30369 2.57099
H -0.97394 -5.00030 1.72697
H   0.07035 -3.72864 2.41250
H -0.74576 -4.88997 3.48864

TS(II-III)$_H$
SCF (TPSS/def2-TZVP) Energy = -1797.03781022
Enthalpy 0K = -1796.529187
Enthalpy 298K = -1796.493439
Free Energy 298K = -1796.599109
Lowest Frequency = -985.0112 cm-1
Second Frequency = 17.6349 cm-1
SCF (M052X-D3,DCM/def2-QZVPP) Energy = -1796.76990084

P   0.02265 -1.68255 -0.20643
N   2.53913 2.87184 -0.55374
N -2.73676 -2.24604 0.60864
N   2.76964 -2.48688 0.16174
N -3.40017 1.48182 0.60312
C -1.34258 -0.52388 -0.26177
C -1.20439 0.87379 -0.26504
C   0.04604 1.50485 -0.18975
H   0.06983 2.58487 -0.09376
C   1.27671 0.83867 -0.12901
C   1.39859 -0.56088 -0.21749
C   2.67563 -1.26422 -0.41146
C   3.71482 -0.77395 -1.23098
H   3.60764 0.18494 -1.72695
C   2.45898 1.71552 0.13168
C   3.40151 1.38298 1.11969
H   3.29627 0.45599 1.67360
C -2.63806 -1.20458 -0.26167
C -3.49722 3.81539 -0.91933
H -3.52793 4.71822 -1.52576
C   3.87549 -3.21258 -0.04778
H   3.89439 -4.19130 0.43375
C   4.96617 -2.80079 -0.81642
C   4.85358 -1.53985 -1.42288

H   5.65391 -1.16841 -2.05998
C -4.55188 3.51629 -0.04788
C -2.38510 1.78741 -0.22772
C   4.44444 2.25821 1.38793
H   5.17212 2.01592 2.15964
C -4.94010 -2.82067 -0.13217
C -3.83221 -3.02437 0.67524
H -3.79943 -3.82609 1.40826
C -2.41046 2.95498 -1.01072
H -1.58693 3.16969 -1.68460
C -4.43435 2.32720 0.67990
H -5.22600 2.04039 1.37322
C -4.85665 -1.75316 -1.04995
H -5.68801 -1.56293 -1.72510
C   6.18417 -3.67202 -0.98979
H   6.38019 -3.87819 -2.04860
H   6.05511 -4.63052 -0.47824
H   7.08173 -3.19156 -0.58181
C   3.56051 3.69621 -0.28635
H   3.58859 4.61602 -0.87184
C   4.55337 3.45544 0.66802
C -3.72903 -0.95655 -1.11624
H -3.66015 -0.15096 -1.83773
C -6.16027 -3.69919 -0.03523
H -7.04675 -3.11594 0.23907
H -6.02471 -4.48079 0.71728
H -6.37384 -4.18543 -0.99386
C -5.74955 4.41861 0.10744
H -5.45872 5.39905 0.50236
H -6.48140 3.98354 0.79414
H -6.24644 4.59052 -0.85420
C   5.66966 4.43997 0.90747
H   5.68532 4.77740 1.95024
H   5.55844 5.32102 0.26876
H   6.64766 3.99156 0.69676
H -1.68596 -2.36755 1.27358
O -0.47886 -2.24259 1.68035
C   0.09586 -3.48679 2.10187
H -0.52997 -3.91766 2.89342
H   0.17040 -4.19506 1.26524
H   1.10080 -3.30492 2.49069

III_H
SCF (TPSS/def2-TZVP) Energy = -1797.05215556
Enthalpy 0K = -1796.538172
Enthalpy 298K = -1796.502348
Free Energy 298K = -1796.608168
Lowest Frequency = 16.7947 cm-1
Second Frequency = 22.0190 cm-1
SCF (M052X-D3,DCM/def2-QZVPP) Energy = -1796.79144987

P   0.00943 -1.67190 0.84916
H -1.80039 -3.11039 0.64208
O -0.04452 -1.49998 2.50746
N   2.28209 2.59780 -1.12165
N -2.60477 -2.74919 0.11497
N   2.89023 -2.38982 0.89094
N -3.47666 1.29632 0.91207
C -1.36984 -0.67872 0.19743
C -1.26795 0.74771 0.06160
C -0.03883 1.38237 0.01315
H -0.01802 2.46605 -0.02746
C   1.22805 0.72731 0.01186
C   1.38138 -0.63048 0.26509
C   2.63017 -1.37927 0.03217
C   3.47142 -1.13851 -1.07350
H   3.22131 -0.35840 -1.78499
C   2.39774 1.63864 -0.18251
C   3.53295 1.55858 0.64271
H   3.58711 0.79539 1.41198
C -2.49653 -1.41611 -0.24354
C -3.64075 3.62773 -0.60883
H -3.69982 4.52723 -1.21833
C   3.98406 -3.13227 0.69454
H   4.14745 -3.93126 1.41918

C 4.89367 -2.95407 -0.35350
C 4.59969 -1.92419 -1.25813
H 5.24750 -1.74888 -2.11492
C -4.68659 3.29834 0.26290
C -2.46578 1.63359 0.08390
C 4.56571 2.46986 0.47324
H 5.44335 2.42164 1.11443
C -4.59876 -3.18320 -1.10960
C -3.57694 -3.60332 -0.29812
H -3.48665 -4.62112 0.06617
C -2.53004 2.79928 -0.70240
H -1.72056 3.03330 -1.38671
C -4.53459 2.11091 0.98770
H -5.32057 1.79783 1.67629
C -4.54161 -1.81970 -1.52280
H -5.30455 -1.44415 -2.20101
C 6.10544 -3.83805 -0.50385
H 6.13348 -4.59933 0.28134
H 7.03374 -3.25734 -0.44296
H 6.10780 -4.35167 -1.47249
C 3.29552 3.46000 -1.27550
H 3.15999 4.21183 -2.05397
C 4.47085 3.45537 -0.51835
C -3.54079 -0.97607 -1.11593
H -3.50602 0.03916 -1.48640
C -5.70259 -4.10634 -1.55083
H -5.55114 -5.11739 -1.16255
H -5.75389 -4.16835 -2.64423
H -6.67955 -3.75341 -1.19946
C -5.91130 4.16363 0.41675
H -5.65279 5.14863 0.82308
H -6.63523 3.70085 1.09371
H -6.40603 4.33020 -0.54699
C 5.56300 4.46724 -0.75442
H 5.76538 5.05214 0.15024
H 5.28679 5.16299 -1.55200
H 6.50159 3.97913 -1.04188
C -0.08811 -0.21380 3.14803
H -0.02461 -0.39810 4.22372
H 0.75784 0.40703 2.83284
H -1.02684 0.30029 2.91430

TS(III-IV)$_H$
SCF (TPSS/def2-TZVP) Energy = -1797.03450004
Enthalpy 0K = -1796.525866
Enthalpy 298K = -1796.490300
Free Energy 298K = -1796.595671
Lowest Frequency = -1272.3392 cm-1
Second Frequency = 18.0630 cm-1
SCF (M052X-D3,DCM/def2-QZVPP) Energy = -1796.76195068

P -0.02194 -1.51168 0.86804
H -0.99492 -2.78855 0.52283
O -0.02114 -1.52690 2.51423
N 2.27891 2.72269 -0.99540
N -2.31167 -2.76292 0.16917
N 2.66968 -2.51762 0.63578
N -3.52534 1.48262 0.84104
C -1.40991 -0.56930 0.27086
C -1.31185 0.83145 0.08760
C -0.06377 1.46346 0.04237
H -0.03976 2.54590 -0.02145
C 1.18917 0.80982 0.03570
C 1.35042 -0.57341 0.23768
C 2.54787 -1.36514 -0.06432
C 3.47915 -1.03867 -1.07271
H 3.34095 -0.14728 -1.67471
C 2.36902 1.71745 -0.10310
C 3.49247 1.58148 0.73127
H 3.52849 0.77746 1.45909
C -2.50189 -1.44147 -0.12377
C -3.63835 3.64850 -0.91389
H -3.67680 4.48292 -1.61115
C 3.70910 -3.31862 0.38642
H 3.75269 -4.23265 0.98013

C 4.70423 -3.06128 -0.56244
C 4.55274 -1.88400 -1.30948
H 5.26931 -1.63929 -2.09117
C -4.70046 3.41998 -0.03056
C -2.50128 1.72287 -0.00396
C 4.53672 2.48914 0.62444
H 5.40507 2.39703 1.27344
C -4.33910 -3.45979 -0.88968
C -3.16386 -3.72706 -0.20124
H -2.88421 -4.74082 0.07835
C -2.53570 2.80320 -0.90346
H -1.70874 2.95965 -1.58895
C -4.57393 2.31234 0.81634
H -5.37298 2.08156 1.52202
C -4.55936 -2.10571 -1.22165
H -5.44791 -1.83512 -1.78839
C 5.85538 -4.01051 -0.77610
H 5.74958 -4.90045 -0.14865
H 6.81407 -3.53822 -0.52943
H 5.91522 -4.33824 -1.82053
C 3.30170 3.58238 -1.08736
H 3.18539 4.37413 -1.82837
C 4.46538 3.52716 -0.31406
C -3.66756 -1.11451 -0.86078
H -3.84975 -0.09005 -1.15445
C -5.30997 -4.55002 -1.26124
H -4.92211 -5.53344 -0.98037
H -5.50471 -4.55899 -2.33990
H -6.27482 -4.41406 -0.75792
C -5.91691 4.30925 0.01465
H -5.65187 5.32838 0.31986
H -6.65537 3.92710 0.72530
H -6.39614 4.37936 -0.96847
C 5.57034 4.53879 -0.48110
H 5.76321 5.07473 0.45540
H 5.31327 5.27702 -1.24617
H 6.50906 4.05688 -0.77836
C -0.00469 -0.29002 3.25685
H 0.03445 -0.56577 4.31260
H 0.87806 0.30378 2.99735
H -0.91212 0.28776 3.05391

IV$_H$
SCF (TPSS/def2-TZVP) Energy = -1797.06756010
Enthalpy 0K = -1796.555473
Enthalpy 298K = -1796.519517
Free Energy 298K = -1796.626374
Lowest Frequency = 18.7338 cm-1
Second Frequency = 24.5654 cm-1
SCF (M052X-D3,DCM/def2-QZVPP) Energy = -1796.80400456

P -0.00436 -1.53074 0.62569
H 0.01609 -2.80237 0.07025
O -0.03108 -1.95503 2.18969
N 2.43675 2.76443 -0.80499
N -2.62403 -2.58949 0.40743
N 2.66163 -2.61837 0.41689
N -3.39572 1.51095 0.78878
C -1.38084 -0.58720 0.09750
C -1.22000 0.80985 -0.02903
C 0.03305 1.44461 -0.02293
H 0.05391 2.52857 -0.03416
C 1.27391 0.79113 0.00046
C 1.39811 -0.61198 0.12213
C 2.58851 -1.41047 -0.19015
C 3.56053 -1.02443 -1.13843
H 3.45914 -0.08672 -1.67339
C 2.47154 1.68210 -0.00381
C 3.55356 1.45005 0.86329
H 3.54464 0.58382 1.51674
C -2.59227 -1.36854 -0.17477
C -3.52885 3.65469 -0.99212
H -3.57363 4.48271 -1.69649
C 3.69273 -3.42198 0.13488
H 3.69363 -4.38343 0.64955

C 4.72819 -3.10808 -0.74959
C 4.62631 -1.87021 -1.40262
H 5.37634 -1.57836 -2.13509
C -4.56937 3.45053 -0.07836
C -2.39951 1.72252 -0.09318
C 4.61419 2.34463 0.88576
H 5.45089 2.17819 1.56094
C -4.75204 -3.06938 -0.64164
C -3.66625 -3.39371 0.17525
H -3.63240 -4.36378 0.67231
C -2.43784 2.79352 -1.00120
H -1.62434 2.93263 -1.70648
C -4.43231 2.35548 0.78346
H -5.21105 2.14915 1.51853
C -4.69309 -1.81860 -1.27470
H -5.48773 -1.51529 -1.95356
C 5.87221 -4.05790 -0.99587
H 5.72141 -4.99915 -0.45920
H 6.82543 -3.63035 -0.66188
H 5.97689 -4.28911 -2.06223
C 3.47358 3.61093 -0.77171
H 3.40229 4.46846 -1.44173
C 4.59994 3.46488 0.04430
C -3.62004 -0.97020 -1.05435
H -3.56464 -0.01781 -1.56858
C -5.90588 -4.01900 -0.83715
H -5.71755 -4.97196 -0.33390
H -6.07847 -4.22545 -1.89980
H -6.83696 -3.60435 -0.43192
C -5.77417 4.35446 -0.01408
H -5.48761 5.37956 0.24833
H -6.48838 4.00222 0.73565
H -6.29041 4.39874 -0.97982
C 5.72543 4.46720 0.01876
H 5.88519 4.91028 1.00847
H 5.51224 5.27772 -0.68404
H 6.66911 3.99787 -0.28284
C -0.23525 -0.93287 3.19189
H -0.33102 -1.46091 4.14099
H 0.62486 -0.25631 3.22818
H -1.14884 -0.36798 2.98364

I_3
SCF (TPSS/def2-TZVP) Energy = -1459.72308905
Enthalpy 0K = -1459.323139
Enthalpy 298K = -1459.297829
Free Energy 298K = -1459.379409
Lowest Frequency = 18.9496 cm-1
Second Frequency = 37.8316 cm-1
SCF (M052X-D3,DCM/def2-QZVPP) Energy = -1459.52662774

P 0.00001 -1.86468 0.05469
C -2.56230 2.62102 0.77021
C 2.98417 -2.54514 -0.77298
C -2.98418 -2.54524 -0.77269
C 3.42706 1.34996 -1.08762
C 1.37733 -0.78122 0.00939
C 1.25610 0.62434 -0.01743
C 0.00001 1.24987 -0.02383
H -0.00000 2.33370 -0.10447
C -1.25607 0.62433 -0.01743
C -1.37734 -0.78123 0.00944
C -2.69205 -1.47632 0.09098
C -3.63830 -1.13676 1.07387
H -3.42377 -0.32624 1.76329
C -2.44192 1.52932 -0.10397
C -3.42702 1.34979 -1.08771
H -3.34499 0.51733 -1.77944
C 2.69204 -1.47635 0.09085
C 3.63982 3.50113 0.67302
H 3.72004 4.33346 1.36667
C -4.18777 -3.24228 -0.66875
H -4.39634 -4.05988 -1.35304
C -5.11893 -2.89135 0.30854
C -4.83625 -1.83820 1.18166

H -5.55046 -1.56429 1.95316
C 4.61134 3.31207 -0.31020
C 2.44195 1.52934 -0.10389
C -4.49750 2.23540 -1.19247
H -5.24429 2.08508 -1.96697
C 5.11888 -2.89146 0.30824
C 4.18774 -3.24221 -0.66912
H 4.39633 -4.05971 -1.35354
C 2.56232 2.62093 0.77046
H 1.81218 2.76889 1.54297
C 4.49753 2.23559 -1.19224
H 5.24433 2.08537 -1.96676
C 4.83619 -1.83845 1.18154
H 5.55039 -1.56469 1.95311
C -3.63981 3.50119 0.67264
H -3.72004 4.33363 1.36617
C -4.61132 3.31200 -0.31057
C 3.63825 -1.13697 1.07383
H 3.42371 -0.32656 1.76338
H 5.44951 3.99823 -0.39016
H 6.05561 -3.43484 0.39215
H -2.26204 -2.81917 -1.53711
H -6.05567 -3.43470 0.39251
H -5.44949 3.99814 -0.39063
H 3.34505 0.51762 -1.77948
H 2.26202 -2.81894 -1.53745
H -1.81217 2.76911 1.54269

II_3
SCF (TPSS/def2-TZVP) Energy = -1536.19617970
Enthalpy 0K = -1535.773617
Enthalpy 298K = -1535.744176
Free Energy 298K = -1535.837267
Lowest Frequency = 16.4919 cm-1
Second Frequency = 17.7911 cm-1
SCF (M052X-D3,DCM/def2-QZVPP) Energy = -1535.99417278

P 0.00005 -1.61537 -0.24518
H 0.00123 -3.95245 0.73193
O 0.00326 -4.72313 1.33033
C 2.56878 2.89322 -0.67025
C -2.97273 -2.37169 0.55208
C 2.97311 -2.37093 0.55189
C -3.41912 1.50593 1.11140
C -1.38320 -0.55092 -0.12756
H 0.01013 -5.48319 0.73127
C -1.25800 0.85039 -0.01739
C -0.00034 1.47247 0.02358
H -0.00048 2.54953 0.16883
C 1.25748 0.85071 -0.01740
C 1.38301 -0.55058 -0.12760
C 2.69533 -1.24489 -0.24162
C 3.65237 -0.84298 -1.19017
H 3.44801 0.01323 -1.82528
C 2.44187 1.74892 0.13265
C 3.41848 1.50675 1.11136
H 3.33028 0.63288 1.74909
C -2.69533 -1.24558 -0.24147
C -3.64723 3.76350 -0.50879
H -3.73363 4.63780 -1.14786
C 4.17519 -3.06450 0.41149
H 4.37147 -3.92760 1.04108
C 5.11773 -2.65199 -0.53002
C 4.84865 -1.54079 -1.33293
H 5.57204 -1.21839 -2.07661
C -4.61045 3.51174 0.46853
C -2.44261 1.74832 0.13265
C 4.48809 2.38327 1.28034
H 5.22820 2.18421 2.05016
C -5.11730 -2.65347 -0.52968
C -4.17459 -3.06566 0.41178
H -4.37053 -3.92883 1.04138
C -2.56985 2.89253 -0.67032
H -1.82600 3.08983 -1.43789
C -4.48896 2.38218 1.28037

H -5.22899 2.18297 2.05022
C -4.84863 -1.54219 -1.33263
H -5.57218 -1.22004 -2.07626
C 3.64594 3.76446 -0.50871
H 3.73209 4.63882 -1.14775
C 4.60926 3.51290 0.46856
C -3.65257 -0.84399 -1.18996
H -3.44852 0.01227 -1.82512
H -3.33067 0.63211 1.74917
H -5.44818 4.19073 0.59833
H -6.05281 -3.19415 -0.64064
H -2.24559 -2.69870 1.29031
H 2.24610 -2.69828 1.29010
H 6.05341 -3.19236 -0.64102
H 1.82486 3.09037 -1.43779
H 5.44682 4.19211 0.59837

TS(OA)$_3$
SCF (TPSS/def2-TZVP) Energy = -1536.11592406
Enthalpy 0K = -1535.697301
Enthalpy 298K = -1535.669531
Free Energy 298K = -1535.756027
Lowest Frequency = -1243.5391 cm-1
Second Frequency = 19.3897 cm-1
SCF (M052X-D3,DCM/def2-QZVPP) Energy = -1535.89880072

P -0.00297 -1.64569 0.07380
H -0.05072 -3.06097 0.49313
O 0.22981 -2.80514 1.94513
C 2.49626 2.76947 -0.76270
C -3.04959 -2.54577 0.44492
C 3.10301 -2.41961 0.62009
C -3.47229 1.39725 1.10722
C -1.42459 -0.66858 0.00118
H -0.57387 -2.75007 2.49118
C -1.27198 0.73870 0.06735
C -0.01736 1.36374 0.09897
H -0.01742 2.44236 0.22030
C 1.24486 0.74353 0.04041
C 1.41389 -0.65621 -0.00057
C 2.71636 -1.34731 -0.20232
C 3.56229 -0.96996 -1.26055
H 3.26731 -0.15582 -1.91525
C 2.42643 1.65558 0.08847
C 3.45742 1.45740 1.02016
H 3.41161 0.60725 1.69332
C -2.71230 -1.36831 -0.24457
C -3.63599 3.65528 -0.51919
H -3.69751 4.52987 -1.16073
C 4.30962 -3.08313 0.39354
H 4.59626 -3.90552 1.04306
C 5.14526 -2.69477 -0.65338
C 4.76453 -1.63611 -1.48148
H 5.40276 -1.33205 -2.30646
C -4.63493 3.40539 0.42226
C -2.45932 1.63820 0.16542
C 4.52486 2.34919 1.09749
H 5.30812 2.18581 1.83218
C -5.10829 -2.75050 -0.80728
C -4.23407 -3.22844 0.16780
H -4.47236 -4.13335 0.71947
C -2.55536 2.78238 -0.64220
H -1.78397 2.97696 -1.38233
C -4.54579 2.27519 1.23760
H -5.31377 2.07766 1.98002
C -4.78178 -1.58752 -1.50928
H -5.44775 -1.21284 -2.28151
C 3.57139 3.65494 -0.69306
H 3.61368 4.50526 -1.36794
C 4.58912 3.44840 0.23872
C -3.59942 -0.90682 -1.23518
H -3.34955 -0.01451 -1.79957
H -3.40721 0.52337 1.74792
H -5.47536 4.08617 0.52166
H -6.03132 -3.28028 -1.02353

H -2.38226 -2.92949 1.21100
H 2.45791 -2.71855 1.44132
H 6.08376 -3.21356 -0.82687
H 1.70892 2.93020 -1.49424
H 5.42531 4.13917 0.29691

III_3
SCF (TPSS/def2-TZVP) Energy = -1536.19953015
Enthalpy 0K = -1535.775671
Enthalpy 298K = -1535.748559
Free Energy 298K = -1535.833359
Lowest Frequency = 22.7707 cm-1
Second Frequency = 32.8210 cm-1
SCF (M052X-D3,DCM/def2-QZVPP) Energy = -1535.99574900

P 0.00005 -1.60048 0.51396
H 0.00012 -2.80883 -0.18990
O -0.00002 -2.33981 1.99609
C 2.49511 2.77152 -0.70980
C -3.04031 -2.54114 0.54127
C 3.04004 -2.54144 0.54087
C -3.52297 1.43334 1.01213
C -1.40743 -0.65026 0.17073
H -0.00090 -1.68452 2.71461
C -1.25329 0.75666 0.14472
C 0.00005 1.39219 0.18052
H 0.00003 2.47322 0.27186
C 1.25339 0.75669 0.14481
C 1.40751 -0.65024 0.17071
C 2.65447 -1.38242 -0.16008
C 3.45391 -0.97820 -1.24800
H 3.16441 -0.10390 -1.82186
C 2.44715 1.65189 0.13645
C 3.52306 1.43326 1.01231
H 3.49898 0.57678 1.67901
C -2.65448 -1.38237 -0.15995
C -3.58497 3.64092 -0.68662
H -3.60650 4.49557 -1.35721
C 4.17971 -3.25882 0.17525
H 4.45532 -4.14714 0.73703
C 4.96671 -2.83521 -0.89472
C 4.59508 -1.68985 -1.60409
H 5.19165 -1.35558 -2.44852
C -4.64549 3.41301 0.19108
C -2.44706 1.65187 0.13627
C 4.60750 2.30681 1.04308
H 5.42472 2.12467 1.73543
C -4.96697 -2.83488 -0.89435
C -4.18011 -3.25838 0.17577
H -4.45593 -4.14651 0.73776
C -2.49498 2.77142 -0.71010
H -1.67646 2.94745 -1.40247
C -4.60741 2.30691 1.04278
H -5.42465 2.12484 1.73513
C -4.59507 -1.68978 -1.60399
H -5.19153 -1.35560 -2.44854
C 3.58511 3.64102 -0.68620
H 3.60666 4.49574 -1.35670
C 4.64560 3.41301 0.19150
C -3.45378 -0.97826 -1.24801
H -3.16408 -0.10416 -1.82205
H -3.49893 0.57693 1.67892
H -5.49397 4.09086 0.21215
H -5.85661 -3.39047 -1.17615
H -2.45183 -2.87320 1.39199
H 2.45146 -2.87357 1.39150
H 5.85626 -3.39091 -1.17662
H 1.67663 2.94764 -1.40219
H 5.49409 4.09086 0.21266

I_{2c}
SCF (TPSS/def2-TZVP) Energy = -1108.14722052
Enthalpy 0K = -1107.875528
Enthalpy 298K = -1107.856455
Free Energy 298K = -1107.924091

Lowest Frequency = 32.7462 cm-1
Second Frequency = 38.0220 cm-1
SCF (M052X-D3,DCM/def2-QZVPP) Energy = -1107.99626629

P -0.00000 -0.25923 0.04919
N 2.80699 -1.03340 -0.41267
N -2.80700 -1.03336 -0.41273
C 1.35197 0.85888 -0.11359
C 1.22368 2.23702 -0.31645
C -0.00000 2.89554 -0.41680
H 0.00000 3.96596 -0.60401
C -1.22369 2.23704 -0.31636
C -1.35197 0.85889 -0.11357
C -2.70185 0.25198 -0.01999
C -3.81571 0.95320 0.47470
H -3.71059 1.97371 0.82956
C 2.70186 0.25199 -0.02003
C -4.00008 -1.62888 -0.33934
H -4.02928 -2.66760 -0.66951
C -5.17205 -1.01316 0.11644
C -5.04899 0.31674 0.53604
H -5.91550 0.85028 0.92097
C 5.17207 -1.01313 0.11646
C 4.00007 -1.62890 -0.33925
H 4.02931 -2.66764 -0.66934
C 5.04902 0.31679 0.53594
H 5.91553 0.85038 0.92079
C -6.48561 -1.75124 0.15242
H -6.92071 -1.73934 1.15810
H -6.35657 -2.79462 -0.14881
H -7.21698 -1.29325 -0.52391
C 3.81572 0.95324 0.47458
H 3.71061 1.97378 0.82935
C 6.48559 -1.75128 0.15250
H 7.21727 -1.29286 -0.52321
H 6.35663 -2.79445 -0.14947
H 6.92026 -1.74008 1.15838
H 2.12853 2.83156 -0.43565
H -2.12854 2.83161 -0.43543

II_{2c}
SCF (TPSS/def2-TZVP) Energy = -1184.62581828
Enthalpy 0K = -1184.330284
Enthalpy 298K = -1184.307817
Free Energy 298K = -1184.384102
Lowest Frequency = 20.0067 cm-1
Second Frequency = 29.3187 cm-1
SCF (M052X-D3,DCM/def2-QZVPP) Energy = -1184.47048275

P 0.07863 -0.03495 -0.20148
N -2.76620 -0.83370 0.19070
N 2.84361 -0.93693 0.13481
C -1.26245 1.05306 0.13605
C -1.11857 2.38219 0.54829
C 0.11379 3.00005 0.75023
H 0.12681 4.02900 1.09897
C 1.33051 2.34844 0.55697
C 1.44532 1.01668 0.14592
C 2.78303 0.40078 -0.02426
C 3.92769 1.14492 -0.35775
H 3.85992 2.21509 -0.52623
C -2.61927 0.48571 -0.04938
C 4.02264 -1.54615 -0.01399
H 4.01623 -2.62713 0.12649
C 5.22269 -0.89405 -0.32319
C 5.14569 0.49227 -0.50135
H 6.03650 1.05939 -0.76262
C -5.11102 -0.70411 -0.39527
C -3.96787 -1.39561 0.01807
H -4.01944 -2.46251 0.23132
C -4.95011 0.66331 -0.65039
H -5.79527 1.25769 -0.99061
C 6.51779 -1.65286 -0.45671
H 6.98486 -1.47244 -1.43136
H 6.35374 -2.72900 -0.35285

H   7.23771 -1.34598 0.31106
C -3.70782 1.26137 -0.48312
H -3.57222 2.31387 -0.71072
C -6.43609 -1.40351 -0.55575
H -7.18549 -0.99316 0.13092
H -6.34153 -2.47337 -0.35165
H -6.82612 -1.28415 -1.57281
H -1.57557 -2.15122 1.04704
O -1.12803 -2.91399 1.47529
H -0.23716 -2.88865 1.09802
H -2.01642 2.96210 0.75755
H   2.24066 2.90614 0.77269

TS(II-III)$_{2c}$
SCF (TPSS/def2-TZVP) Energy = -1184.60247708
Enthalpy 0K = -1184.309810
Enthalpy 298K = -1184.289420
Free Energy 298K = -1184.358303
Lowest Frequency = -1099.8978 cm-1
Second Frequency = 32.1207 cm-1
SCF (M052X-D3,DCM/def2-QZVPP) Energy = -1184.43684774

P   0.05269 -0.02693 -0.41185
N -2.66292 -0.86501 0.25705
N   2.67011 -0.96115 0.02392
C -1.32678 1.08007 -0.10453
C -1.19459 2.43317 0.22280
C   0.02365 3.07543 0.43130
H   0.02479 4.12316 0.71452
C   1.24398 2.39431 0.35457
C   1.40061 1.05170 0.01722
C   2.70652 0.39289 -0.03976
C   3.93687 1.06878 -0.17420
H   3.96071 2.15058 -0.26050
C -2.62990 0.44518 -0.13409
C   3.81947 -1.64210 -0.04589
H   3.72886 -2.72807 0.00066
C   5.08451 -1.06018 -0.15798
C   5.11543 0.34153 -0.22345
H   6.06663 0.85981 -0.32777
C -5.02786 -1.03437 -0.12086
C -3.80649 -1.57248 0.25604
H -3.72242 -2.60463 0.58798
C -5.01847 0.32245 -0.50983
H -5.94652 0.79519 -0.82363
C   6.33739 -1.89695 -0.20563
H   6.91222 -1.70362 -1.11894
H   6.09604 -2.96371 -0.17942
H   6.99601 -1.68058 0.64412
C -3.84463 1.05179 -0.51874
H -3.83735 2.08394 -0.85208
C -6.29117 -1.85513 -0.10994
H -7.04931 -1.40829 0.54331
H -6.09724 -2.87143 0.24388
H -6.72650 -1.92488 -1.11358
H -1.53455 -1.22745 0.78431
O -0.38128 -1.16224 1.21788
H   0.12934 -1.96858 1.03319
H -2.10527 3.01217 0.38009
H   2.14297 2.96486 0.58960

III$_{2c}$
SCF (TPSS/def2-TZVP) Energy = -1184.61735113
Enthalpy 0K = -1184.320003
Enthalpy 298K = -1184.299012
Free Energy 298K = -1184.369923
Lowest Frequency = 22.3637 cm-1
Second Frequency = 41.6154 cm-1
SCF (M052X-D3,DCM/def2-QZVPP) Energy = -1184.45801385

P   0.02822 -0.15024 0.50241
H -1.86224 -1.49640 0.27665
O   0.08480 -0.14669 2.18539
N -2.75372 -1.01562 0.09929
N   2.77675 -0.96633 0.31832

C -1.37637 0.96940 0.13725
H 0.21414 0.75163 2.53266
C -1.23122 2.37799 0.05948
C -0.01263 3.01713 -0.01275
H 0.01005 4.10151 -0.06571
C 1.22071 2.31818 -0.09585
C 1.39042 0.95629 0.02688
C 2.69633 0.30487 -0.13447
C 3.81779 0.92400 -0.72618
H 3.74525 1.93055 -1.12614
C -2.64152 0.35904 -0.03665
C 3.93719 -1.61868 0.21549
H 3.93923 -2.64035 0.59802
C 5.10689 -1.08377 -0.33369
C 5.01448 0.22992 -0.81643
H 5.88060 0.70364 -1.27464
C -5.08206 -1.12365 -0.38597
C -3.89291 -1.73320 -0.07974
H -3.79178 -2.80570 0.04854
C -5.03548 0.29643 -0.52815
H -5.94904 0.82843 -0.78370
C 6.38278 -1.88269 -0.40747
H 6.23792 -2.88781 -0.00054
H 7.18856 -1.40268 0.16082
H 6.73202 -1.98464 -1.44184
C -3.87346 1.00521 -0.36859
H -3.87182 2.07845 -0.51236
C -6.35892 -1.90058 -0.56242
H -6.19048 -2.97268 -0.42852
H -6.78104 -1.74748 -1.56265
H -7.11906 -1.58573 0.16248
H -2.12314 3.00438 0.04387
H 2.10570 2.93018 -0.27392

TS(III-IV)$_{2c}$
SCF (TPSS/def2-TZVP) Energy = -1184.59896609
Enthalpy 0K = -1184.307044
Enthalpy 298K = -1184.286412
Free Energy 298K = -1184.356248
Lowest Frequency = -1315.1299 cm-1
Second Frequency = 27.9982 cm-1
SCF (M052X-D3,DCM/def2-QZVPP) Energy = -1184.42858933

P -0.04366 0.11659 0.58997
H -1.17481 -1.06655 0.32619
O 0.07866 0.02319 2.24517
N -2.52496 -0.90692 0.18856
N 2.55120 -0.96397 0.22870
C -1.40580 1.18373 0.15793
H 0.32939 0.88018 2.62828
C -1.25674 2.55568 -0.08073
C -0.01128 3.16345 -0.21282
H 0.03722 4.23343 -0.38890
C 1.19140 2.43170 -0.23484
C 1.34189 1.06627 -0.00544
C 2.60106 0.33552 -0.14507
C 3.80242 0.89308 -0.62967
H 3.84200 1.92953 -0.94983
C -2.63397 0.43896 -0.01067
C 3.65581 -1.70853 0.14024
H 3.55059 -2.74767 0.45434
C 4.89416 -1.24364 -0.31385
C 4.93866 0.10239 -0.70655
H 5.86753 0.52898 -1.08012
C -4.81254 -1.32285 -0.36969
C -3.54428 -1.75422 -0.00307
H -3.32666 -2.80703 0.16402
C -4.95950 0.06824 -0.57007
H -5.92860 0.46194 -0.86935
C 6.10154 -2.14359 -0.37758
H 5.85133 -3.15608 -0.04732
H 6.91169 -1.77229 0.26142
H 6.49784 -2.21020 -1.39774
C -3.89838 0.93726 -0.40847
H -4.02484 1.99790 -0.59559

C -5.96179 -2.28348 -0.53256
H -5.63442 -3.31588 -0.38056
H -6.40015 -2.21254 -1.53469
H -6.76190 -2.07573 0.18798
H -2.14214 3.17781 -0.21517
H   2.09204 2.99611 -0.47709

IV_{2c}
SCF (TPSS/def2-TZVP) Energy = -1184.63431720
Enthalpy 0K = -1184.338931
Enthalpy 298K = -1184.317903
Free Energy 298K = -1184.389161
Lowest Frequency = 25.2801 cm-1
Second Frequency = 37.6457 cm-1
SCF (M052X-D3,DCM/def2-QZVPP) Energy = -1184.47308018

P -0.00001 0.00868 0.33277
H -0.00004 -1.15128 -0.43165
O   0.00018 -0.71634 1.79606
N -2.64578 -0.98018 0.06339
N   2.64570 -0.98021 0.06298
C -1.39074 1.04019 0.05505
H -0.00030 -0.05828 2.50998
C -1.22262 2.42877 0.00854
C -0.00001 3.10171 0.01781
H -0.00003 4.18619 -0.01253
C   1.22262 2.42878 0.00855
C   1.39075 1.04022 0.05507
C   2.68521 0.36549 -0.06932
C   3.90462 1.02346 -0.33059
H   3.93842 2.10007 -0.46241
C -2.68522 0.36548 -0.06934
C   3.77992 -1.67744 -0.04821
H   3.68173 -2.75760 0.06395
C   5.03745 -1.11562 -0.28627
C   5.07003 0.27934 -0.43143
H   6.01400 0.78276 -0.63047
C -5.03748 -1.11559 -0.28624
C -3.78001 -1.67740 -0.04780
H -3.68189 -2.75753 0.06468
C -5.06996 0.27931 -0.43191
H -6.01385 0.78270 -0.63136
C   6.27828 -1.96585 -0.38136
H   6.03333 -3.02696 -0.27841
H   6.99958 -1.70975 0.40388
H   6.78414 -1.82802 -1.34410
C -3.90453 1.02342 -0.33109
H -3.93824 2.09998 -0.46335
C -6.27835 -1.96578 -0.38109
H -6.03317 -3.02705 -0.28027
H -6.78558 -1.82632 -1.34287
H -6.99857 -1.71113 0.40562
H -2.12281 3.03722 -0.05868
H   2.12279 3.03724 -0.05866

$TS(IV-V)_{2c}$
SCF (TPSS/def2-TZVP) Energy = -1261.07983118
Enthalpy 0K = -1260.765370
Enthalpy 298K = -1260.743274
Free Energy 298K = -1260.817259
Lowest Frequency = -1425.5980 cm-1
Second Frequency = 20.4933 cm-1
SCF (M052X-D3,DCM/def2-QZVPP) Energy = -1260.91044064

P -0.10951 -0.10861 0.53882
H -0.00615 1.15936 -0.03837
O -0.24317 -0.01128 2.06809
N   2.60215 0.96059 0.42353
N -2.69671 0.96987 0.21745
C   1.37003 -1.06048 0.08145
H   1.65096 -1.41710 1.43103
C   1.19826 -2.35721 -0.53073
C -0.00418 -2.94662 -0.81773
H -0.01166 -3.96140 -1.20405
C -1.25451 -2.26648 -0.72426

C -1.45749 -0.99396 -0.24660
C -2.73589 -0.27950 -0.29594
C -3.93106 -0.80242 -0.82757
H -3.96159 -1.80084 -1.25232
C 2.57128 -0.23106 -0.20585
C -3.81196 1.70471 0.22099
H -3.71745 2.70403 0.64602
C -5.04756 1.27023 -0.26841
C -5.07898 -0.02548 -0.80568
H -6.00739 -0.42346 -1.21003
C 4.76727 1.44220 -0.54710
C 3.65971 1.75872 0.24505
H 3.62375 2.71277 0.77133
C 4.71792 0.20827 -1.20953
H 5.53518 -0.09144 -1.86254
C -6.27059 2.14884 -0.21549
H -6.02936 3.13001 0.20310
H -7.05546 1.70135 0.40573
H -6.69492 2.30171 -1.21457
C 3.62139 -0.62927 -1.05267
H 3.57147 -1.56819 -1.59479
C 5.93705 2.38388 -0.68066
H 5.74710 3.32023 -0.14809
H 6.13682 2.62645 -1.73067
H 6.85270 1.94340 -0.26849
H 0.73269 -0.74087 2.60658
O 1.63450 -1.41442 2.68791
H 1.31626 -2.28673 2.97168
H 2.10485 -2.92699 -0.73312
H -2.11808 -2.80688 -1.11126

V_{2c}
SCF (TPSS/def2-TZVP) Energy = -1184.62647781
Enthalpy 0K = -1184.330276
Enthalpy 298K = -1184.309562
Free Energy 298K = -1184.381721
Lowest Frequency = 17.6758 cm-1
Second Frequency = 24.6883 cm-1
SCF (M052X-D3,DCM/def2-QZVPP) Energy = -1184.47297462

P -0.01875 -1.13782 1.15242
H 0.20886 0.19457 1.54234
O -0.56915 -2.05863 2.18724
N 3.11362 0.16298 1.22610
N -2.38701 0.46364 0.83636
C 1.65466 -1.63503 0.50777
H 2.13036 -2.10201 1.37852
C 1.55515 -2.62603 -0.61513
C 0.51427 -2.69428 -1.47064
H 0.55100 -3.41923 -2.28028
C -0.66478 -1.85037 -1.41197
C -1.00577 -1.03790 -0.37463
C -2.21378 -0.20400 -0.32358
C -3.14073 -0.08168 -1.37512
H -2.99550 -0.61356 -2.31015
C 2.49251 -0.40378 0.17834
C -3.46691 1.23487 0.98155
H -3.55822 1.74446 1.94056
C -4.44830 1.41569 0.00056
C -4.25337 0.72877 -1.20623
H -4.97683 0.82898 -2.01240
C 4.00889 1.87463 -0.24027
C 3.84391 1.26333 1.00762
H 4.33251 1.68569 1.88572
C 3.34837 1.27333 -1.31729
H 3.43135 1.69838 -2.31530
C -5.64341 2.30191 0.23630
H -5.60257 2.75887 1.22883
H -6.57783 1.73339 0.16386
H -5.69426 3.10651 -0.50630
C 2.58519 0.12834 -1.11283
H 2.07141 -0.35318 -1.93861
C 4.85553 3.11149 -0.40315
H 5.26500 3.43483 0.55794
H 4.27117 3.94039 -0.81837

H   5.69491 2.93118 -1.08450
H   2.39557 -3.30436 -0.74599
H -1.32733 -1.91070 -2.27518

2. Experimental Procedures

2.1 General Remarks

Unless otherwise stated, all reactions and workups were performed following standard Schlenk techniques or using an argon filled *Unilab* glovebox by *MBraun* (H_2O < 0.1 ppm, O_2 < 0.1 ppm). Room temperature refers to T = 23 °C, while elevated reaction temperatures are referred to the respective oil bath's temperature. Dry acetonitrile; toluene; dichloromethane and *n*-pentane were collected from a solvent purification system, *MB SPS-800* by *MBraun*, and additionally stored over 4 Å molecular sieves prior to use. Solvents and reagents were degassed using the *freeze-pump-thaw* method (three cycles) or by purging with argon. Dioxane was dried by stirring over ground KOH for 7 d, followed by distillation and storage over 4 Å molecular sieves. CD_2Cl_2 and $CDCl_3$ were dried over CaH_2, distilled, and degassed. 2,2-Dimethylpropanenitrile was degassed and stored over 4 Å molecular sieves. NEt_3 was distilled, degassed, and stored over 4 Å molecular sieves. PCl_3 was refluxed and distilled prior to use. All other commercially available chemicals were used without further purification. 1,2-Bis(tributylstannyl)ethyne[8] and tetraphenylphosphinine **3**[9] were prepared according to literature procedures. Preparative, inert column chromatography was performed on dried silica gel (particle size: 0.040-0.063 mm, surface area: 500 m^2/g) by *Sigma-Aldrich* as the stationary phase under an argon atmosphere. Microwave reactions were conducted using a *Discover 2.0* reactor by *CEM*. The ^1H, $^1H\{^{31}P\}$, $^{13}C\{^1H\}$, ^{31}P and $^{31}P\{^1H\}$ NMR spectra were recorded on *JEOL* (ECX 400, Lamba 400, ECP 500, ECZ 600) and *Bruker* (AVANCE 500, AVANCE 700) spectrometers and all chemical shifts are reported relative to the residual resonance in the respective deuterated solvents. Quantitative ^{31}P NMR spectroscopic measurements were carried out using an internal triphenylphosphine standard. Infrared (IR) spectra were recorded on a Fourier-transform *Nicolet iS FT-IR* spectrometer by *Thermo Fisher Scientific*. High resolution mass spectrometry (HRMS) was performed on an *Agilent 6210 ESI-TOF*.

2.2 Synthetic Procedures

Phosphinines **2a** and **2b** were synthesized *via* [4+2]-cycloaddition-cycloreversion reactions between 4,6-di-*tert*-butyl-1,3,2-diazaphosphinine and the respective alkynes. The starting materials were obtained over three- and two-step syntheses respectively, following modified literature procedures.

Bis(η^5-cyclopentadienyl)dimethyltitanium (A)

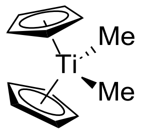

A

$C_{12}H_{16}Ti$

M = 208.13 g/mol

The title compound **A** was prepared using a modified literature procedure.[10] In a 500 mL dried Schlenk flask, dried and degassed *n*-pentane (250 mL) was cooled to –78 °C. Titanocene dichloride (10 g, 40 mmol, 1.0 equiv) was added under vigorous stirring, resulting in a dark red suspension. Methyllithium (1.6 M in Et_2O, 49 mL, 88 mmol, 2.2 equiv) was added slowly. The flask was allowed to warm up to rt and the mixture was stirred for 1.5 h, resulting in bright orange solution containing an off-white precipitate. The mixture was filtered over a Celite® plug, which was washed thoroughly with dry and degassed *n*-pentane (4 x 20 mL). The solvent was removed *in vacuo*, affording an orange solid. Dried and degassed toluene (60 mL) was added, and the yield was calculated by quantitative ^{1}H NMR spectroscopy. All steps, including the workup, were conducted under the exclusion of light. Dimethyltitanocene **A** was obtained as a 0.55 M solution in toluene (7.0 g, 33 mmol, 83%).

^{1}H NMR (400 MHz, $CDCl_3$): δ = 6.12 (s, 10H, H_{Cp}), −0.06 (s, 6H, Me) ppm.

The spectroscopic data is consistent with the literature.[11]

Bis(η^5-cyclopentadienyl)*N,N*-(2,2,6,6-tetramethylheptane-3,5-diimine)titanium (B)

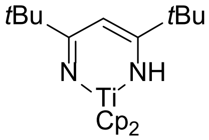

B

$C_{21}H_{30}N_2Ti$

M = 358.35 g/mol

The title compound **B** was prepared using a modified literature procedure.[12] In a dried 100 mL microwave vial, dried and degassed 2,2-dimethylpropanenitrile (4.2 mL, 38 mmol, 2.3 equiv) was added to a 0.39 M toluene solution of **A** (42 mL, 16 mmol, 1.0 equiv). The mixture was put to a microwave reaction at 150 °C for 15 min, employing 300 W, inducing a colour change from deep red to dark brown. After the mixture was cooled down to 50 °C, it was transferred into a dried 250 mL Schlenk flask, and the microwave vial rinsed with dried and degassed toluene (20 mL). The yield was directly calculated from the reaction mixture by quantitative ^{1}H NMR spectroscopy. Titanacycle **B** was obtained as a 0.24 M solution in toluene (5.8 g, 16 mmol, >99%).

^{1}H NMR (400 MHz, $CDCl_3$): δ = 5.87 (s, 10H, H_{Cp}), 4.76 (s, 1H, C*H*), 1.21 (s, 9H, H_{tBu}), 1,16 (s, 9H, H_{tBu}) ppm.

The spectroscopic data is consistent with the literature.[12]

4,6-Di-*tert*-butyl-1,3,2-diazaphosphinine (C)

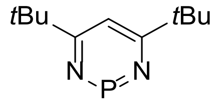

C

$C_{11}H_{19}N_2P$

M = 210.26 g/mol

The title compound **C** was prepared using a modified literature procedure.[12] A 0.24 M toluene solution of **B** (66 mL, 16 mmol, 1.0 equiv) was directly used for the synthesis of **C**. The Schlenk flask was cooled to −78 °C and dried and degassed NEt_3 (11 mL, 81 mmol, 5.0 equiv) was added. The mixture was stirred for 5 min at −78 °C and freshly distilled and degassed PCl_3 (1.5 mL, 17 mmol, 1.1 equiv) was added dropwise under vigorous stirring. The mixture was heated to 80 °C and stirred for 3 h, resulting in a brown solution containing a dark green precipitate. The flask was allowed to cool down to r. t. and thoroughly sonicated. The mixture was filtered, and the residue thoroughly washed with dry toluene (4 x 30 mL). The dark brown filtrates were combined in a dried 250 mL Schlenk flask, and the yield was calculated *via* quantitative ^{31}P NMR spectroscopy. Diazaphosphinine **C** was obtained as a 0.06 M solution in toluene (0.93 g, 4.4 mmol, 28%).

^{31}P NMR (162 MHz): δ = 268.4 ppm.

The spectroscopic data is consistent with the literature.[12]**1,2-bis(pyridin-2-yl)ethyne (D)**

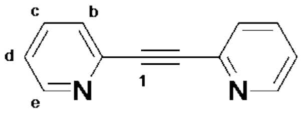

D

$C_{12}H_8N_2$
M = 180.21 g/mol

1,2-Bis(tributylstannyl)ethyne (6.4 g, 11 mmol, 1.0 equiv.), 2-bromopyridine (2.1 mL, 22 mmol, 2.1 equiv.), and $Pd(PPh_3)_4$ (0.22 g, 1.5 mol%) were dissolved in dried and degassed dioxane (80 mL) and stirred at 100 °C for 15 h. The solvent was removed *in vacuo* and the residue dissolved in CH_2Cl_2 (25 mL). The crude product was transferred to the aqueous phase with HCl (20 mL, 6.0 M) and the aqueous phase was washed with CH_2Cl_2 (5 x 30 mL). The combined organic phases were extracted with HCl (2 x 20 mL, 6.0 M). The combined aqueous phases were rendered basic with an aqueous ammonium hydroxide solution (100 mL, 25%). The aqueous phase was extracted with CH_2Cl_2 (3 x 25 mL). The organic phase was washed with KF solution (total, 23 mL) and dried over NaCl solution (saturated, 20 mL) and $MgSO_4$. The solvent was removed *in vacuo*, and **D** was obtained as yellow crystals *via* sublimation (120 °C, 0.007 mbar, 1.3 g, 6.9 mmol, 65%).

^{1}H NMR (400 MHz; $CDCl_3$): δ = 8.60 (ddd, $^3J_{H\text{-}e\text{-}H\text{-}d}$ = 4.9 Hz, $^4J_{H\text{-}e\text{-}H\text{-}c}$ = 1.7 Hz, $^5J_{H\text{-}e\text{-}H\text{-}b}$ = 1.0 Hz, 2H, H-e), 7.67 (ddd, $^3J_{H\text{-}c\text{-}H\text{-}d/e}$ = 7.7 Hz, $^4J_{H\text{-}c\text{-}H\text{-}e}$ = 1.8 Hz, 2H, H-c), 7.58 (ddd, $^3J_{H\text{-}d\text{-}H\text{-}e/c}$ = 7.8 Hz, $^4J_{H\text{-}c\text{-}H\text{-}}$ = 1.0, 2H, H_p), 7.24 (ddd, $^3J_{H\text{-}b\text{-}H\text{-}c}$ = 7.6 Hz, $^4J_{H\text{-}b\text{-}H\text{-}d}$ = 4.9 Hz, $^5J_{H\text{-}b\text{-}H\text{-}e}$ = 1.3 Hz, 2H, H-e) ppm.

^{13}C{^{1}H} NMR (100 MHz; $CDCl_3$): δ = 150.1 (2C, C-e), 142.2 (2C, C-a) 136.1 (2C; C-c), 127.7 (2C, C-b), 123.2 (2C, C-d), 87.6 (2C, C-1) ppm.

The spectroscopic data is consistent with the literature.[[13]]

1,2-bis(5-methylpyridin-2-yl)ethyne (E)

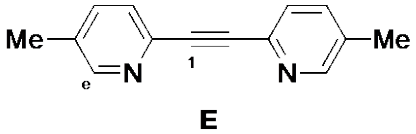

E

$C_{13}H_{10}N_2$
M = 208.26 g/mol

In a Schlenk flask, $Pd(PPh_3)_4$ (0.23 g, 0.20 mmol, 1.5 mol%) was dissolved in dried and degassed dioxane (40 mL) and 2-bromo-5-methylpyridine (5.4 g, 32 mmol, 2.1 equiv) was added. 1,2-Bis(tributylstannyl)ethyne (9.1 g, 15 mmol, 1.0 equiv) was added directly into the solution and the flask was immersed into a preheated oil bath. The mixture was stirred at

100 °C for 16 h. Under vigorous stirring, the flask was slowly allowed to cool down to rt, causing the desired product to precipitate. This step should be done very carefully to prevent the product from crystallising too quickly, causing an incorporation of impurities. The flask was further cooled to 0 °C to ensure the complete precipitation of the product and the supernatant was filtered off. The residue was washed with ice-cold *n*-pentane (4 x 10 mL) and the product was thoroughly dried *in vacuo*. Acetylene **E** was obtained as a light yellow, crystalline powder (2.4 g, 12 mmol, 80%).

^{1}H NMR (400 MHz, $CDCl_3$): δ = 8.44 (s, 2H, H-e), 7.49 (m, 4H, H-b/H-c), 2.35 (s, 6H, Me) ppm.

^{13}C{^{1}H} NMR (151 MHz, $CDCl_3$): δ = 150.8 (2C, C-e), 140.1 (2C, C-a), 136.8 (2C; C-c), 133.4 (2C, C-d), 127.3 (2C, C-b), 87.6 (2C, C-1), 18.7 (2C, Me) ppm.

HRMS-ESI: *m/z* calculated for $C_{14}H_{13}N_2^+$ [M+H]$^+$: 209.1074. Found: 209.1082.

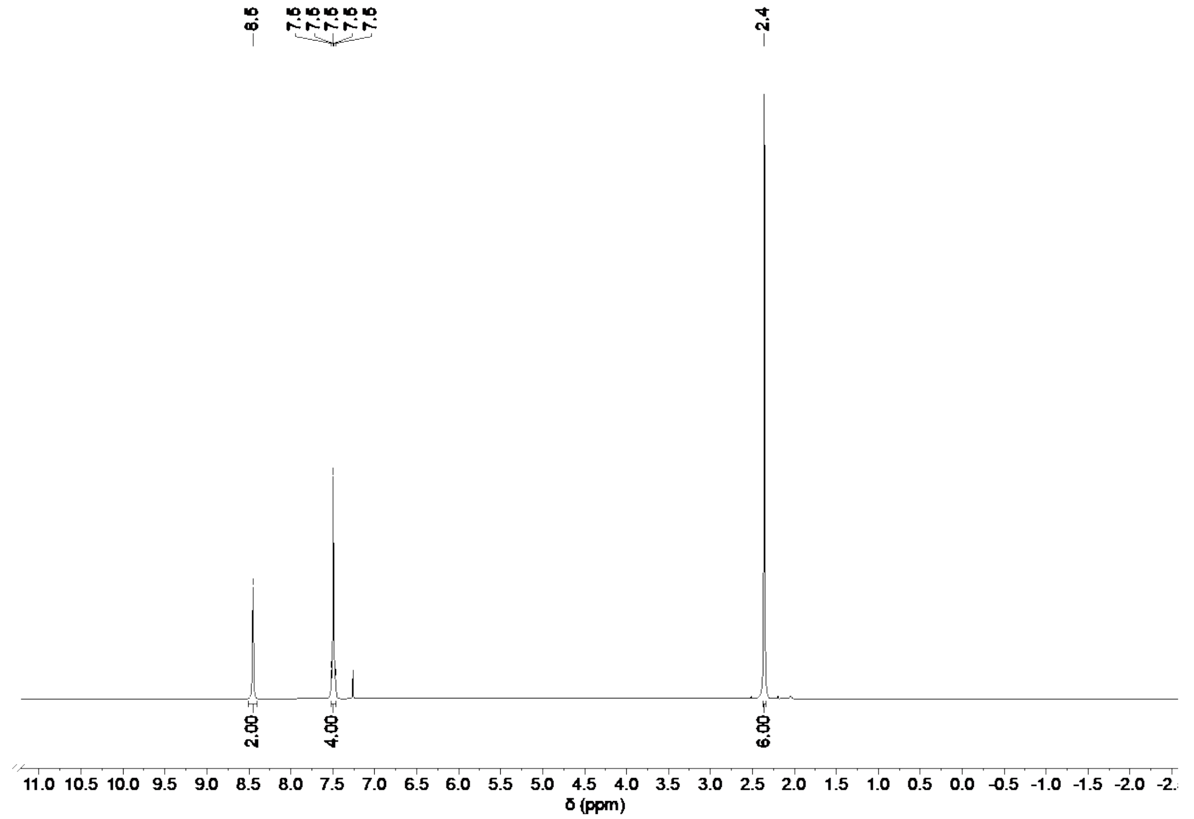

Figure S2.1: ^{1}H NMR spectrum ($CDCl_3$) of **E**.

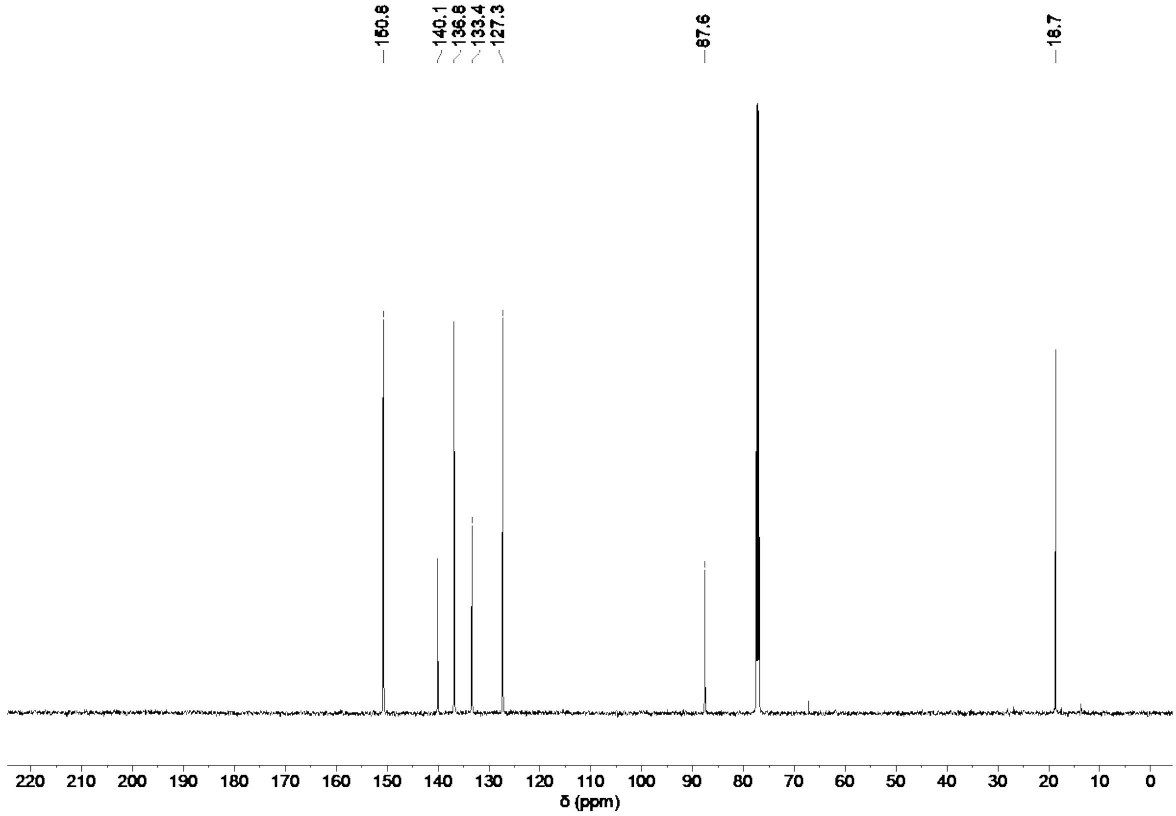

Figure S2.2: $^{13}C\{^1H\}$ NMR spectrum ($CDCl_3$) of **E**.

2,3,5,6-Tetrakis(pyridin-2-yl)-phosphinine (2a)

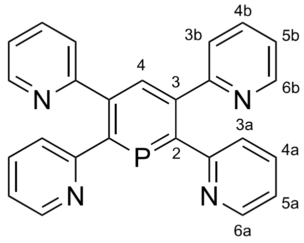

2a

$C_{25}H_{17}N_4P$
M = 404.41 g/mol

To a 0.39 M solution of diazaphosphinine **B** in toluene (19.5 mL, 2.0 mmol, 1.0 equiv) in a 100 mL Schlenk flask, acetylene **D** (0.89 g, 50.0 mmol, 2.5 equiv) was added. The reaction mixture was stirred at 130 °C for 4 d. The solution was allowed to cool down without stirring, causing the product to precipitate. The supernatant was removed by filtration. The crude product was recrystallized three times from acetonitrile (40 mL). **2a** was isolated as white crystals (0.44 g, 1.1 mmol, 53%).

^{1}H NMR (400 MHz, CD_2Cl_2): δ = 8.56 (ddd, $^{3}J_{H\text{-}6a\text{-}H5a}$ = 4.8 Hz, $^{4}J_{H6a\text{-}H4a}$ = 1.8 Hz, $^{4}J_{H6a\text{-}H3a}$ = 1.0 Hz, 2H, H-6a), 8.54 (ddd, $^{3}J_{H6b\text{-}H5b}$ = 4.9 Hz, $^{4}J_{H6b\text{-}H4b}$ = 1.9 Hz, $^{4}J_{H6b\text{-}H3b}$ = 1.9 Hz, 2H, H-6b), 8.07 (d, $^{4}J_{H\text{-}4\text{-}P}$ = 3.3 Hz, 1H, H-4), 7.54 (ddd, $^{3}J_{H4a\text{-}H5a}$ = 7.7 Hz, $^{3}J_{H4a\text{-}H3a}$ = 7.7 Hz, $^{4}J_{H4a\text{-}H3a}$ = 1.8 Hz, 2H, H-4a), 7.49 (ddd, $^{3}J_{H4b\text{-}H5b}$ = 7.7 Hz, $^{3}J_{H4b\text{-}H3b}$ = 7.7 Hz, $^{4}J_{H4b\text{-}H3b}$ = 1.8 Hz, 2H, H-4b), 7.21 (ddd, $^{3}J_{H3a\text{-}H4a}$ = 7.8 Hz, $^{4}J_{H3a\text{-}H5a}$ = 1.1 Hz, $^{4}J_{H3a\text{-}H6a}$ = 1.1 Hz, 2H, H-3a), 7.18-7.13 (m, 4H, H-5a, H-5a), 7.08 (ddd, $^{3}J_{H3b\text{-}H4b}$ = 7.8 Hz, $^{4}J_{H3b\text{-}H5b}$ = 1.1 Hz, $^{4}J_{H3b\text{-}H6b}$ Hz, 2H, H-3b) ppm.

^{13}C{^{1}H} NMR (100 MHz, CD_2Cl_2): δ = 169.0 (d, $^{1}J_{C\text{-}2\text{-}P}$ = 53.6 Hz, 2C, C-2), 160.3 (d, $^{2}J_{C2,P}$ = 23.7 Hz, 2C, C-3), 159.6 (s, 2C, C-2a), 149.8 (s, 2C, C-6a), 149.7 (s, 2C, C-6b), 145.3 (d, $^{3}J_{C\text{-}5,P}$ = 11.2 Hz, 2C, C-2b), 136.9 (d, $^{3}J_{C\text{-}4,P}$ = 12 Hz, 1C, C-4), 136.0 (s, 2C, C-4a), 135.9 (s, 2C, C-4b), 125.3 (s, 2C, C-5a), 125.2 (s, 2C, C-5b), 122.2 (s, 2C, C-3b), 122.1 (s, 2C, C-3a) ppm.

^{31}P NMR (162 MHz, CD_2Cl_2): δ = 213.9 (d, $^{4}J_{P\text{-}H\text{-}4}$ = 3.5 Hz) ppm.

HRMS-ESI: *m/z* calculated for $C_{25}H_{18}N_4P^+$ [M+H]$^+$: 405.1269, Found: 405.1358.

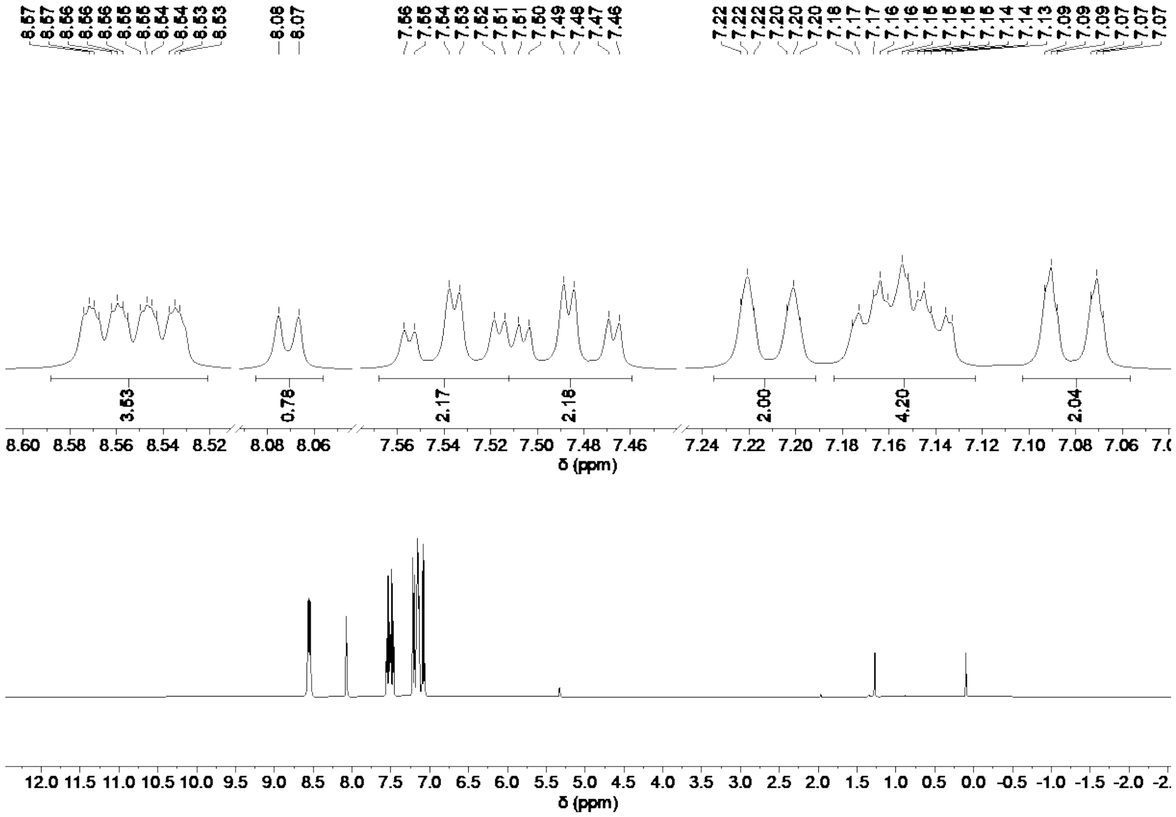

Figure S2.3: ^{1}H NMR spectrum (CD_2Cl_2) of **2a**.

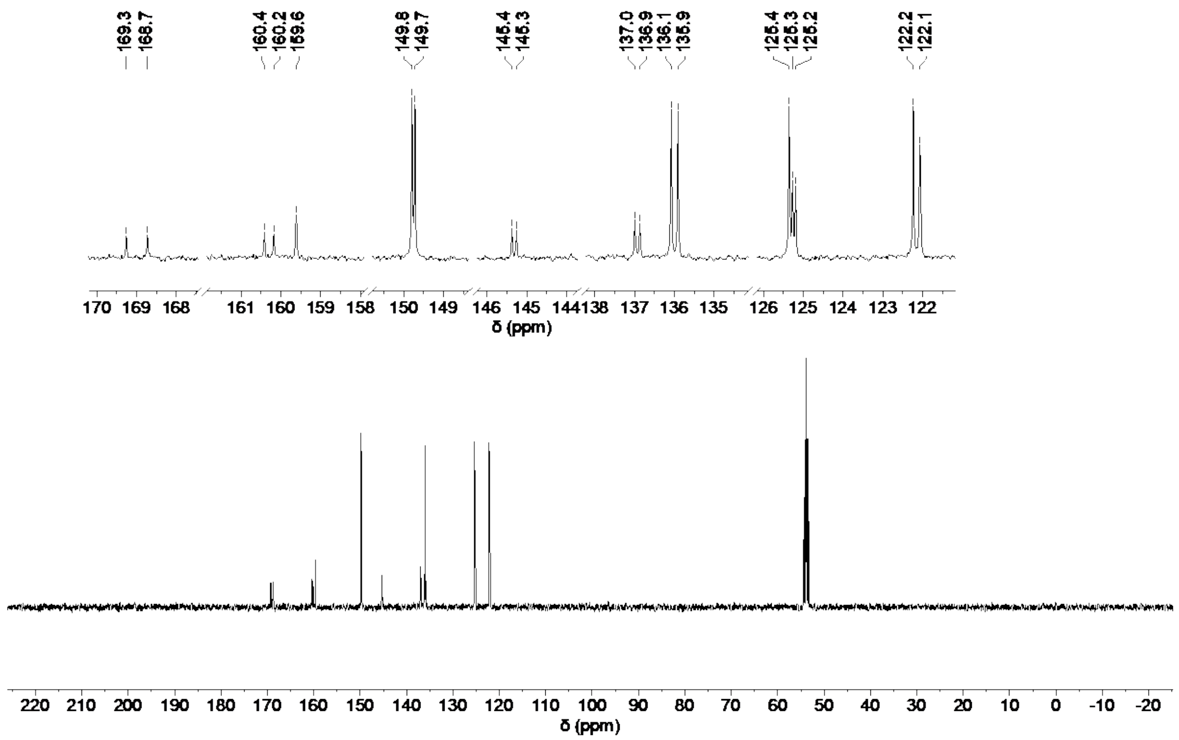

Figure S2.4: $^{13}C\{^{1}H\}$ NMR spectrum (CD_2Cl_2) of **2a.**

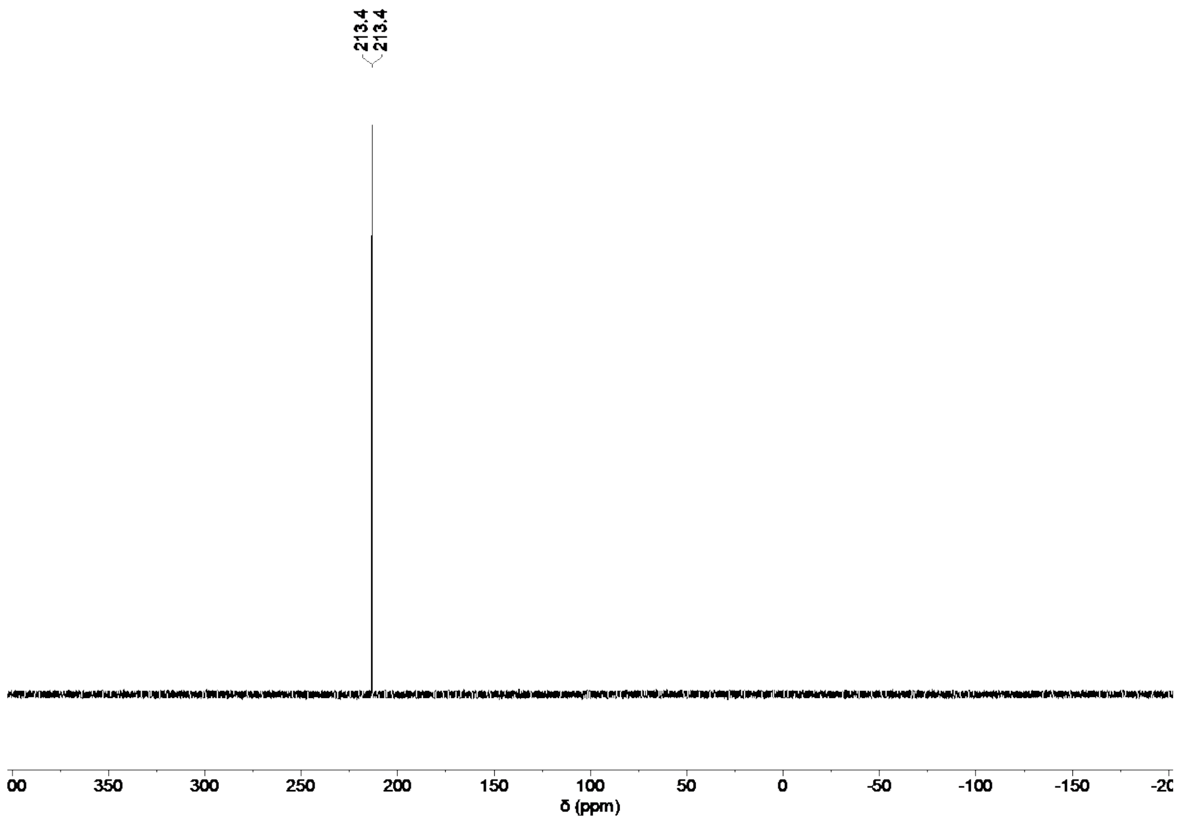

Figure S2.5: ^{31}P NMR spectrum (CD_2Cl_2) of **2a**.

2,3,5,6-Tetrakis(5-methylpyridin-2-yl)phosphinine (2b)

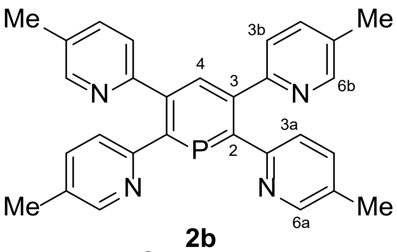

2b

$C_{29}H_{25}N_4P$
M = 460.52 g/mol

In a 100 mL Schlenk flask, 1,2-bis(5-methylpyridin-2-yl)ethyne **D** (0.79 g, 3.8 mmol, 4.0 equiv) was added to a 0.04 M toluene solution of diazaphosphinine **C** (25 mL, 1.0 mmol, 1.0 equiv). The mixture was immersed into a preheated oil bath and stirred at 130 °C for 16 h. The mixture was allowed to cool down to room temperature without stirring, leading to the precipitation of a crystalline, red solid. The supernatant was filtered off and the residue was washed with dry acetonitrile (2 x 5 mL). The crude solid was recrystallised from dry acetonitrile (4 x 5 mL) to afford phosphinine **2b** as a white powder (92 mg, 0.20 mmol, 20%).

^{1}H NMR (600 MHz, CD_2Cl_2): δ = 8.41 (d, $^4J_{H\text{-}6',H\text{-}4'}$ = 2.4 Hz, 2H, H-6a), 8.38 (d, $^4J_{H\text{-}6'',H\text{-}4''}$ = 2.4 Hz, 2H, H-6b), 8.00 (d, $^4J_{H\text{-}4,P}$ = 3.1 Hz, 1H, H-4), 7.34 (dd, $^3J_{H\text{-}4',H\text{-}3'}$ = 8.0 Hz, $^4J_{H\text{-}4',H\text{-}6'}$ = 2.3 Hz, 2H, H-4a), 7.29 (dd, $^3J_{H\text{-}4'',H\text{-}3''}$ = 8.0 Hz, $^4J_{H\text{-}4'',H\text{-}6''}$ = 2.1 Hz, 2H, H-4b), 7.06 (d, $^3J_{H\text{-}3',H\text{-}4'}$ = 7.9 Hz, 2H, H-3a), 6.93 (d, $^3J_{H\text{-}3'',H\text{-}4''}$ = 8.0 Hz, 2H, H-3b), 2.31 (s, 6H, Me-a), 2.30 (s, 6H, Me-b) ppm.

$^{13}C\{^1H\}$ NMR (151 MHz, CD_2Cl_2): δ = 168.8 (d, $^2J_{C\text{-}2',P}$ = 53.0 Hz, 2C, C-2a), 157.7 (d, $^3J_{C\text{-}2'',P}$ = 23.6 Hz, 2C, C-2b), 156.9 (s, 2C, C-2/C-6), 150.2 (s, 2C, C-6a), 150.16 (s, 2C, C-6b), 145.0 (d, $^2J_{C\text{-}5,P}$ = 11.2 Hz, 2C, C-3/C-5), 137.1 (d, $^3J_{C\text{-}4,P}$ = 12.3 Hz, 1C, C-4), 136.6 (s, 2C, C-4a), 136.4 (s, 2C, C-4b), 131.8 (s, 2C, C-5a), 131.7 (s, 2C, C-5b), 124.8 (s, 2C, C-3b), 124.7 (d, $^3J_{C\text{-}3',P}$ = 7.2 Hz, 2C, C-3a), 18.29 (s, 4C, Me-a/b) ppm.

$^{31}P\{^1H\}$ NMR (162 MHz, CD_2Cl_2): δ = 211.3 ppm.

IR (ATR): $\tilde{\nu}$ = 2996 (w), 2921 (w), 1596 (m), 1562 (m), 1478 (vs), 1376 (w), 1222 (w), 1028 (m), 842 (w) cm^{-1}.

HRMS-ESI: *m/z* calculated for $C_{29}H_{24}N_4P^+$ $[M+H]^+$: 461.1890. Found: 461.1953.

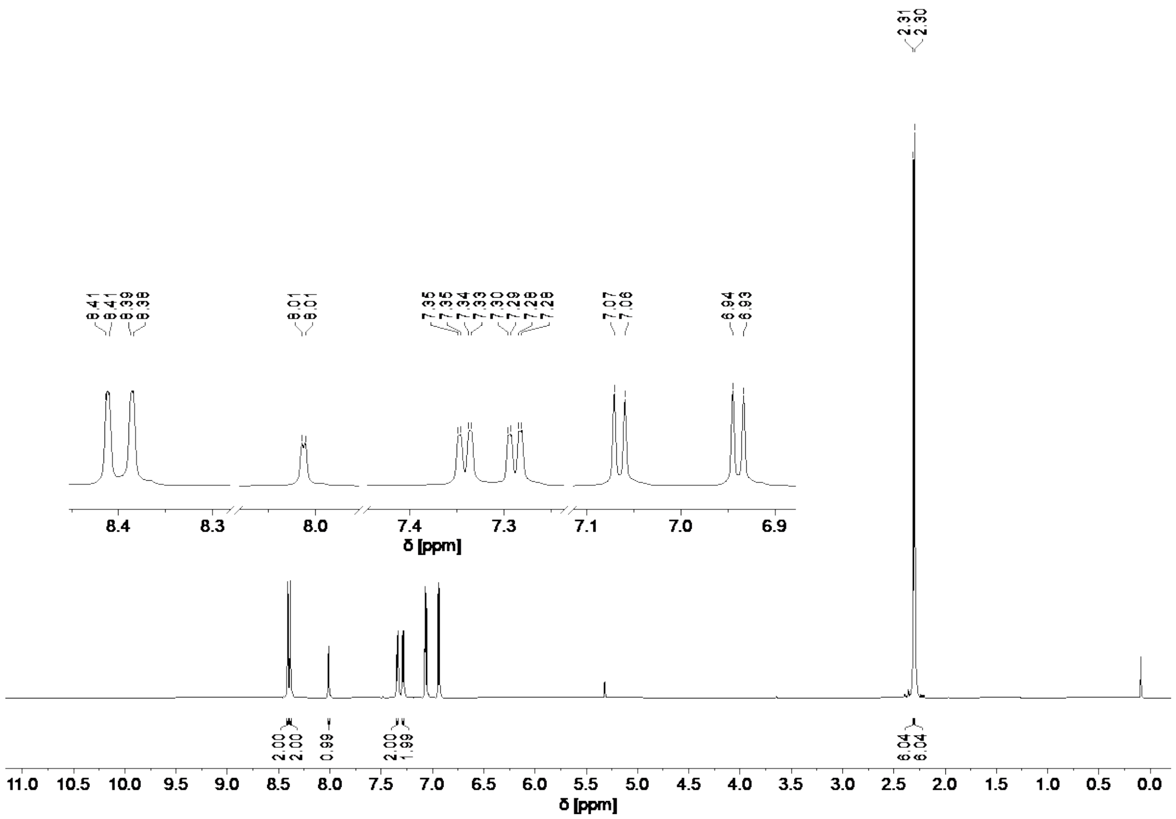

Figure S2.6: ^{1}H NMR spectrum (CD_2Cl_2) of **2b**.

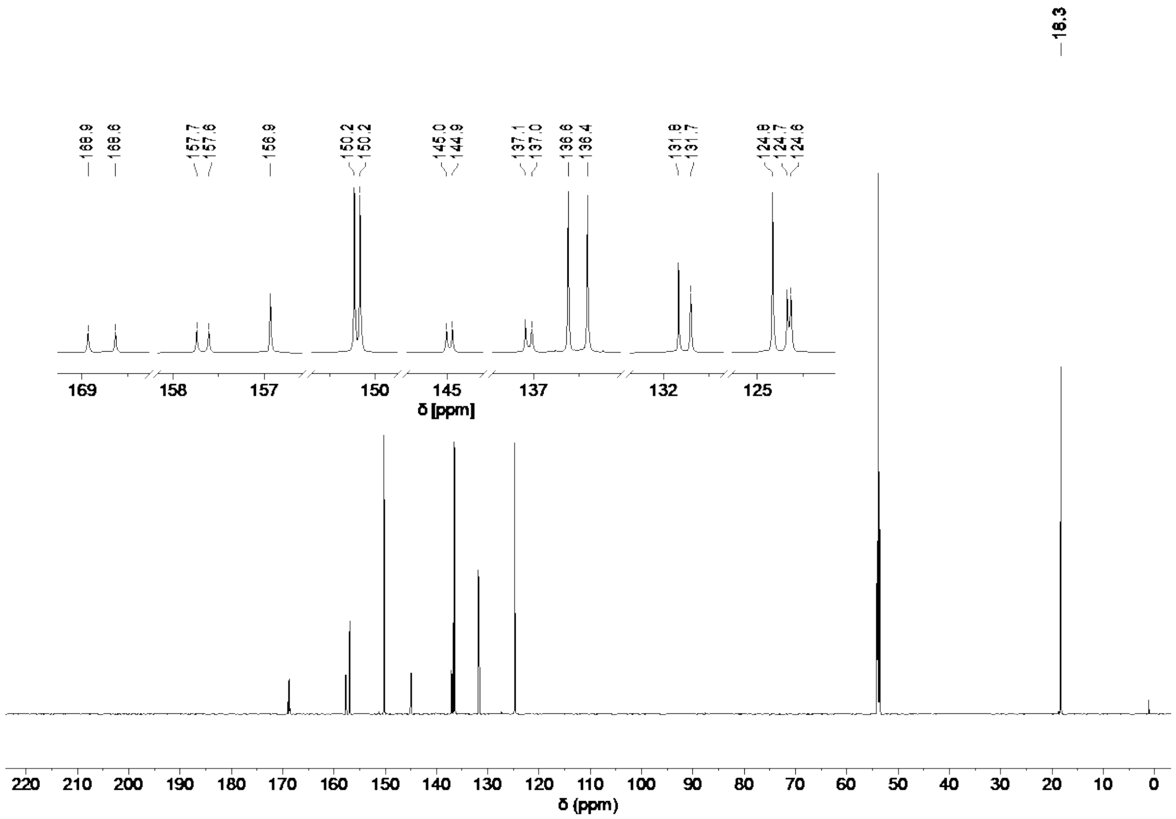

Figure S2.7: $^{13}C\{^{1}H\}$ NMR spectrum (CD_2Cl_2) of **2b**.

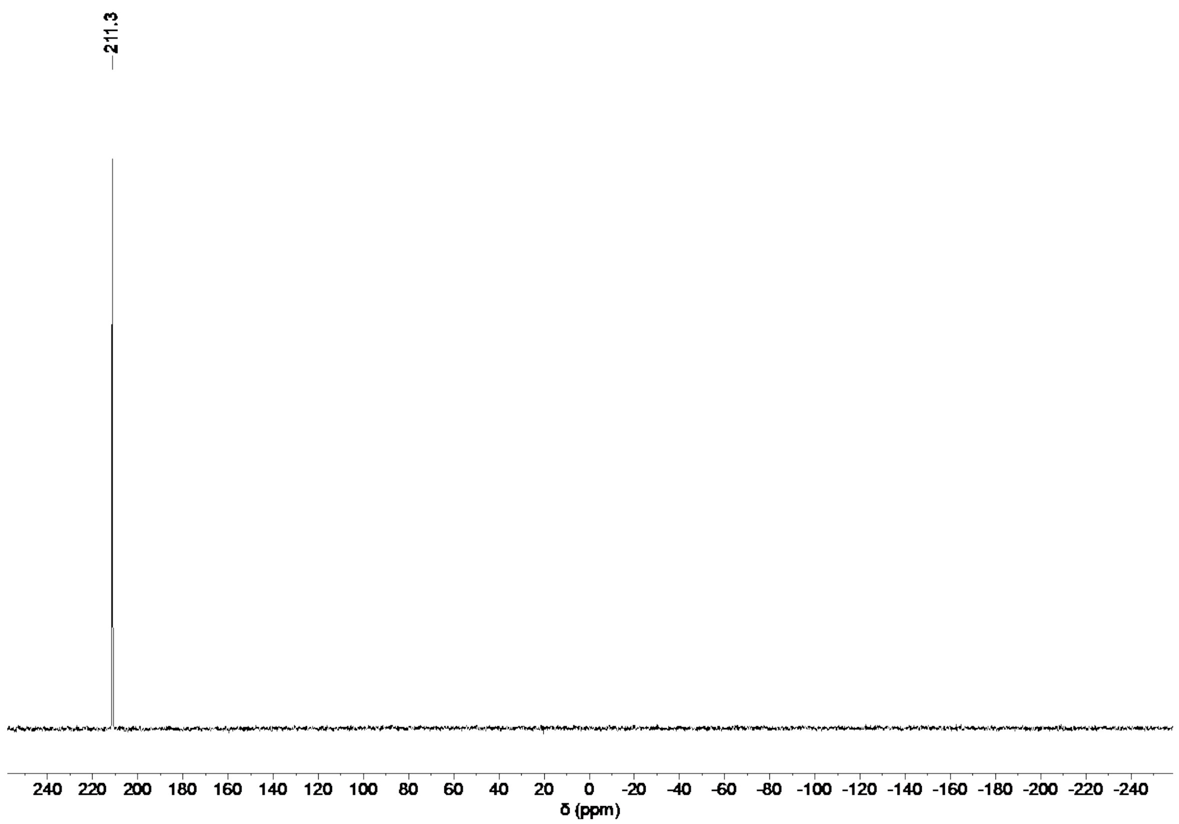

Figure S2.8: ^{31}P NMR spectrum (CD_2Cl_2) of **2b.**

2.3 Oxidative Addition of Water

General Procedure for the Synthesis of 4b

In a J. Young NMR tube, phosphinine **2b** (1.0 equiv) was dissolved in dried and degassed CD_2Cl_2 (0.60 mL) and degassed H_2O (10 equiv) was added using a microliter syringe. The sample was mixed by thorough shaking and the reaction conversion was controlled by ^{31}P NMR spectroscopy. If needed, the reaction mixtures can be heated to T = 70 °C for 1 h (**2b**) to accelerate the conversion. Once the starting material was fully consumed, the reaction mixture was filtered over $MgSO_4$ in a glovebox and the solvent was removed. The residue was carefully dried *in vacuo* without heating, to avoid the re-formation of the respective phosphinines. For storage over longer time in solution, the drying step must be left out.

The same procedure was followed during the screening of reaction conditions using a variety of solvents, as well as for all comparative reactions between phosphinines **2a/b** and **3** (1.0 equiv).

(1S,2S)-2,3,5,6-Tetrakis(pyiridin-2-yl)-anti-1*H*,2*H*-dihydrophosphinine 1-oxide (4a)

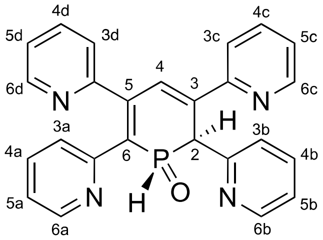

4a

$C_{25}H_{18}N_4OP$
M = 422.43 g/mol

For the synthesis of 1,2-dihydrophosphinine oxide **4a** phosphinine **2a** (59 mg, 0.15 mmol, 1.0 equiv) was introduced in a Schlenk flask and dissolved in CH_2Cl_2 (4 mL). Subsequently H_2O (25 µL, 1.4 mmol, 9.5 equiv) was added. The reaction mixture was stirred for 16 h at rt. The orange solution was filtered over $MgSO_4$ and the solvent removed *in vacou*. The product was obtained as a dark red powder (53 mg, 0.13 mmol, 86%) in a mixture of 97:3 with the syn-product **4b**.

^{1}H NMR (400 MHz, CD_2Cl_2): δ = 8.76 ($^3J_{H\text{-}6b\text{-}H\text{-}5b}$ = 4.9 Hz, $^4J_{H\text{-}6b\text{-}H\text{-}4b}$ = 1.8 Hz, $^5J_{H\text{-}6b\text{-}H3b}$ = 1.0 Hz, 1H, H-6b), 8.58 (ddd, $^3J_{H\text{-}6c\text{-}H\text{-}5c}$ = 4.8 Hz, $^4J_{H\text{-}6c\text{-}H\text{-}4c}$ = 1.7 Hz, $^5J_{H6c\text{-}H3c}$ = 1.0 Hz, 1H, H-6c), 8.57-8.54 (m, 2H, H-6d, H-6a), 7.84 (d, $^4J_{H\text{-}4,P}$ = 1.9 Hz, 1H, H-4), 7.76-7.62 (m, 2H, H-3d, H-4c), 7.63-7.50 (m, 2H, H-4a, H-4b), 7.39-7.25 (m, 3H, H-3a, H-4d, H-5d), 7.37 (d, $^1J_{P\text{-}H\text{-}P}$ = 530 Hz,1H, P-H), 7.23-7.10 (m, 3H, H-3c, H-5b, H-5c), 7.08 (dd, $^3J_{H\text{-}5a\text{-}H\text{-}6a}$ = 7.5 Hz, $^4J_{H\text{-}5a\text{-}H\text{-}4a}$ = 4.9 Hz, 1H, H-5a), 6.73 (d, $^3J_{H\text{-}3b,H\text{-}4c}$ = 8.0 Hz, 1H, H-3b), 5.62 (d, $^2J_{H\text{-}2,P}$ = 22.0 Hz, 1H, H-2) ppm.

^{13}C{^{1}H} NMR (176 MHz, CD_2Cl_2): δ =158.1 (d, $^{2}J_{C\text{-}2b,P}$ = 13.9 Hz, 1C, C-2b), 155.6 (d, $^{3}J_{C\text{-}2d,P}$ = 6.1 Hz, 1C, C-2d), 153.4 (s, C-2c), 150.7 (s, 1C, C-6a), 150.6 (s, 1C, C-6b), 150.1 (s, 1C, C-6c), 149.8 (s, 1C, C-6d), 148.1 (d, $^{2}J_{C\text{-}2a,P}$ = 4.9 Hz, 1C, C-2a), 138.3 (d, $^{2}J_{C\text{-}6,P}$ = 6.7 Hz, 1C, C-6), 137.6 (s, 1C, C-4b), 137.1 (s, 1C, C-4d), 136.8 (s, 1C, C-4a), 136.3 (s, 1C, C-4c), 129.1 (d, $^{3}J_{C\text{-}4,P}$ = 16.5 Hz, 1C, C-4), 129.0 (s, 1C, C-5), 128.5 (s, 1C, C-3), 125.3 (d, $^{3}J_{C\text{-}3d\text{-}P}$ = 5.3 Hz, 1C, C-3d), 125.2 (s, 1C, C-3b), 123.9 (s, 1C, C-3a), 123.6 (s, 1C, C-5b) 122.8 (s, 1C, C-5c), 122.8 (s, 1C, C-5a), 122.5 (d, $^{4}J_{C\text{-}3c,P}$ = 4.6 Hz, 1C, C-3c) 121.2 (s, 1C, C-5d), 47.4 (d, $^{2}J_{C\text{-}6,P}$ = 63.8 Hz, 1C, C-2), ppm.

^{31}P NMR (162 MHz, CD_2Cl_2): δ = 21.1 (dd, $^{1}J_{P,H\text{-}P}$ = 528 Hz, $^{2}J_{P,H\text{-}2}$ = 22.0 Hz) ppm.

^{31}P{^{1}H} NMR (162 MHz, CD_2Cl_2): δ = 21.1 (s) ppm.

HRMS (ESI) (m/z): *m/z* calculated for $C_{25}H_{18}N_4OPH^+$ $[M+H]^+$: 423.1369, found: 423.1429; calculated for $C_{25}H_{18}N_4OPNa^+$ $[M+Na]^+$: 445.1188, found: 445.1218.

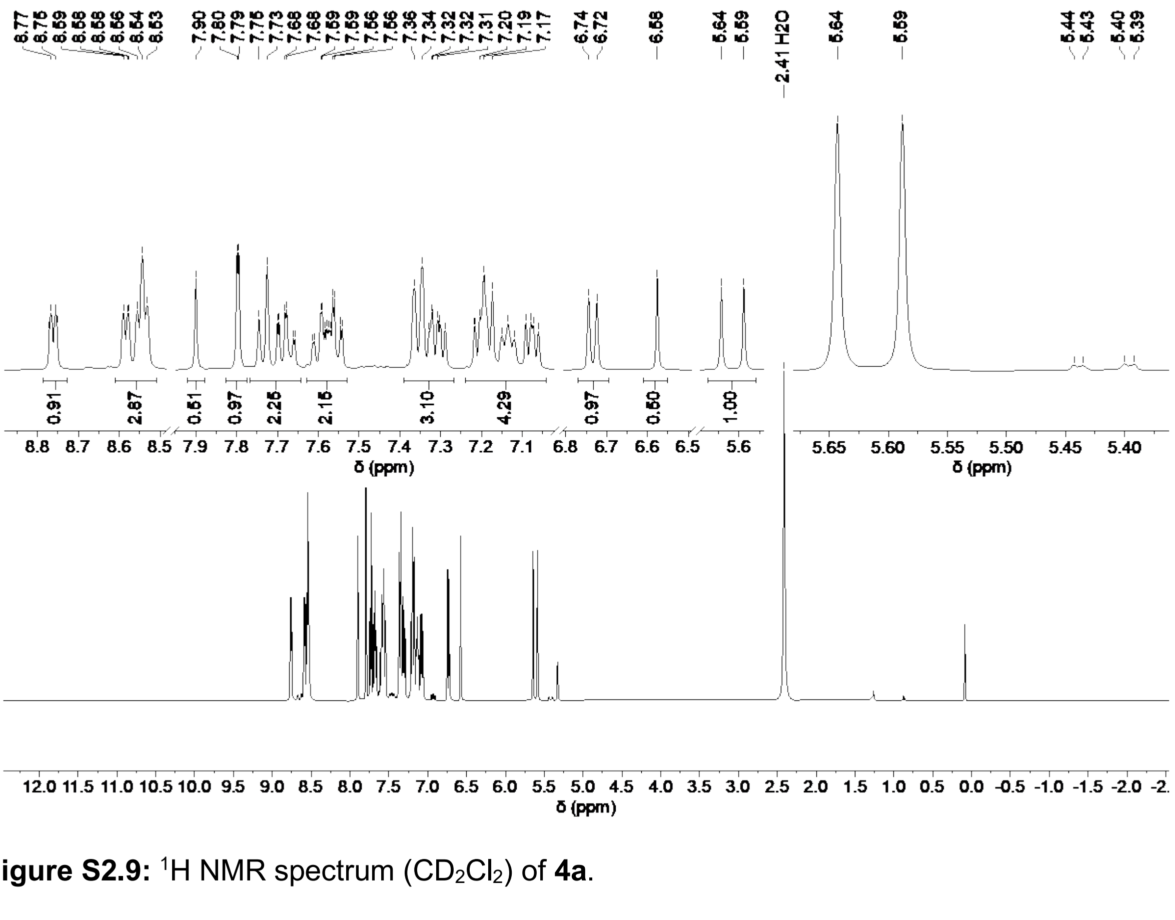

Figure S2.9: ^{1}H NMR spectrum (CD_2Cl_2) of **4a**.

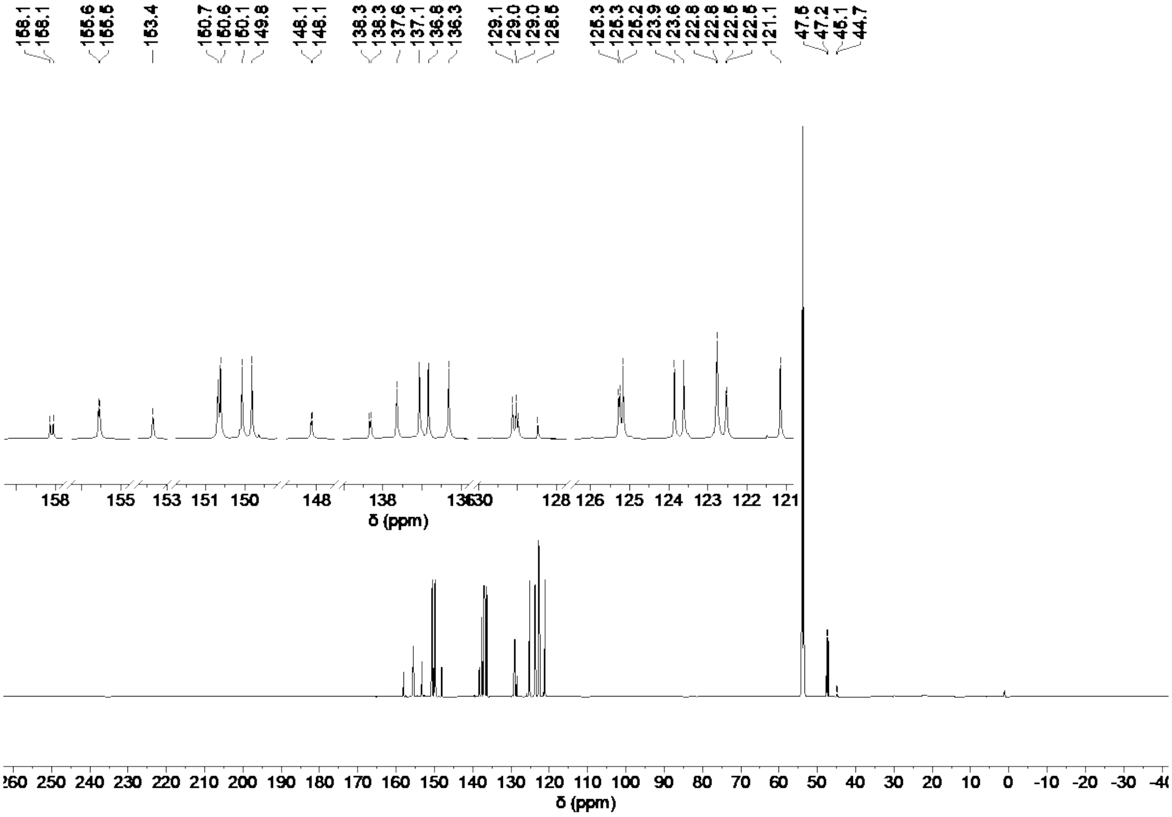

Figure S2.10: $^{13}C\{^1H\}$ NMR spectrum (CD_2Cl_2) of **4a**.

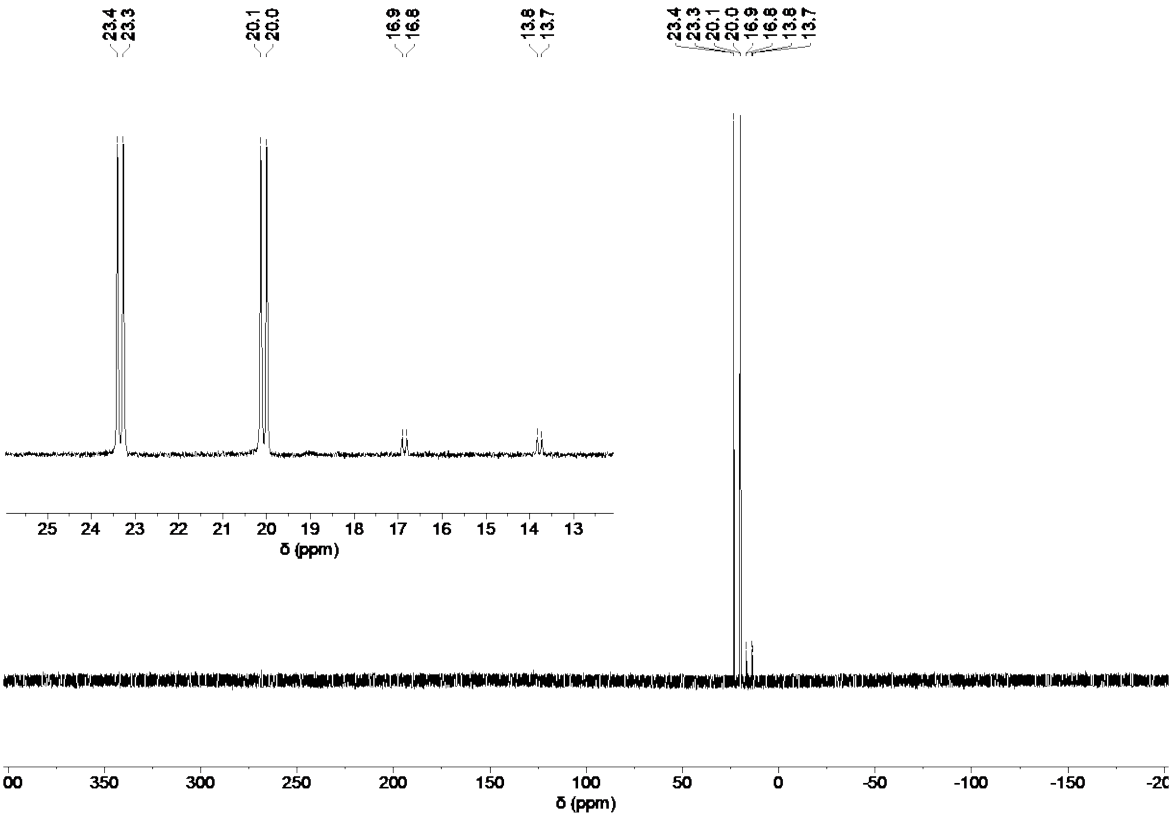

Figure S2.11: ^{31}P NMR spectrum (CD_2Cl_2) of **4a** and **5a**.

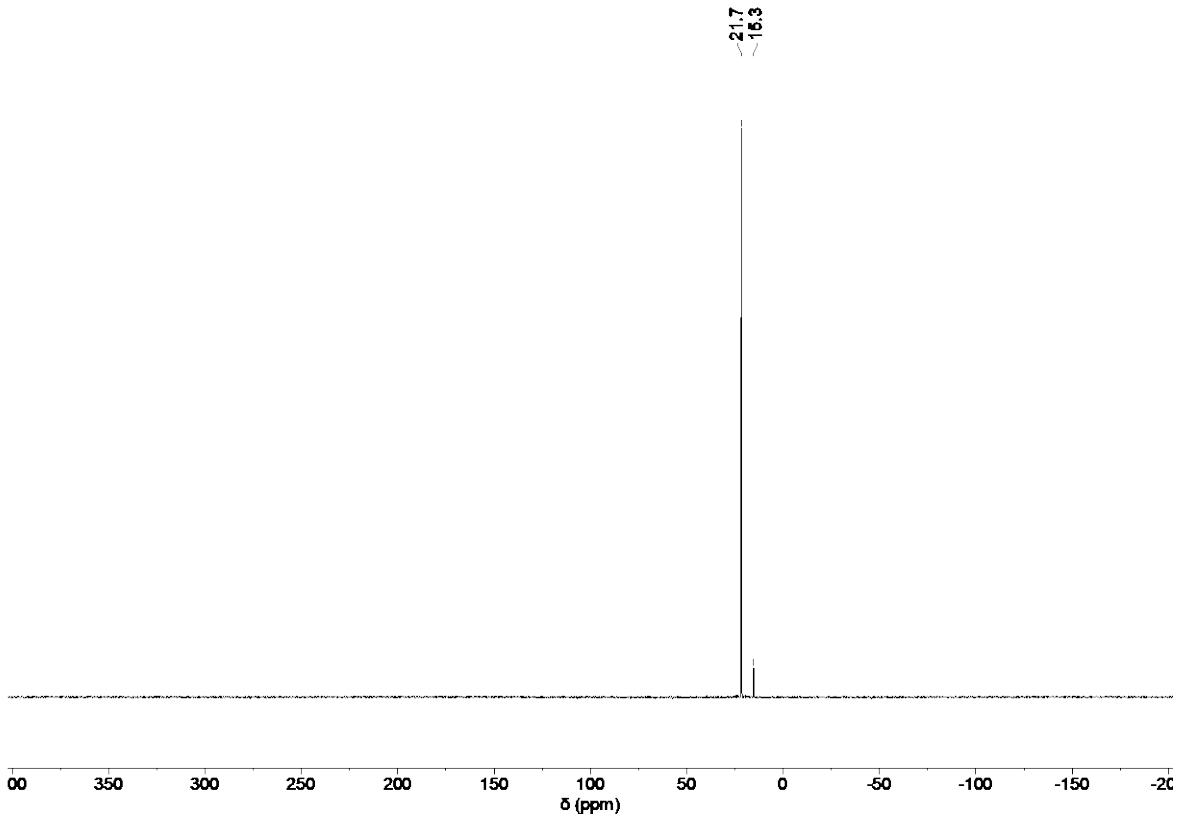

Figure S2.12: $^{31}P\{^1H\}$ NMR spectrum (CD_2Cl_2) of **4a** and **5a**.

(1S,2S)-2,3,5,6-Tetrakis(pyiridin-2-yl)-syn-1*H*,2*H*-dihydrophosphinine 1-oxide (5a)

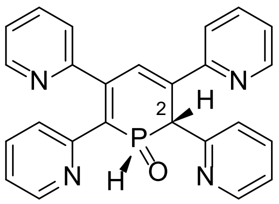

5a

$C_{25}H_{19}N_4OP$
M = 422.43 g/mol

Phosphinine 1-oxide **5a** was not isolated, but obtained as a diastereomeric mixture with its anti-diastereomer in a ratio of 97:3 (anti:syn). Its structure was confirmed by thorough analysis of the ^1H, $^{13}C\{^1H\}$ and ^{31}P NMR spectra obtained for **4a** (**Figures S2.9–11**). **5a** could not be completely characterized by NMR spectroscopy, as most of its signals overlap with the ones of **4a**, but the assignable signals are listed below.

^{1}H NMR (400 MHz, CD_2Cl_2): δ = 5.42 (dd, $^2J_{H\text{-}2,P}$ = 17 Hz, $^3J_{H\text{-}2,H\text{-}P}$ = 3.3 Hz, 1H, H-2) ppm.

$^{13}C\{^1H\}$ NMR (176 MHz, CD_2Cl_2): δ = 44.9 (d, $^1J_{C\text{-}2,P}$ = 65 Hz, 1C, C-2) ppm.

^{31}P NMR (162 MHz, CD_2Cl_2): δ = 15.3 (dd, $^1J_{P,H\text{-}P}$ = 498 Hz, $^2J_{P,H\text{-}2}$ = 16.5 Hz) ppm.

$^{31}P\{^1H\}$ NMR (162 MHz, CD_2Cl_2): δ = 15.3 (s) ppm.

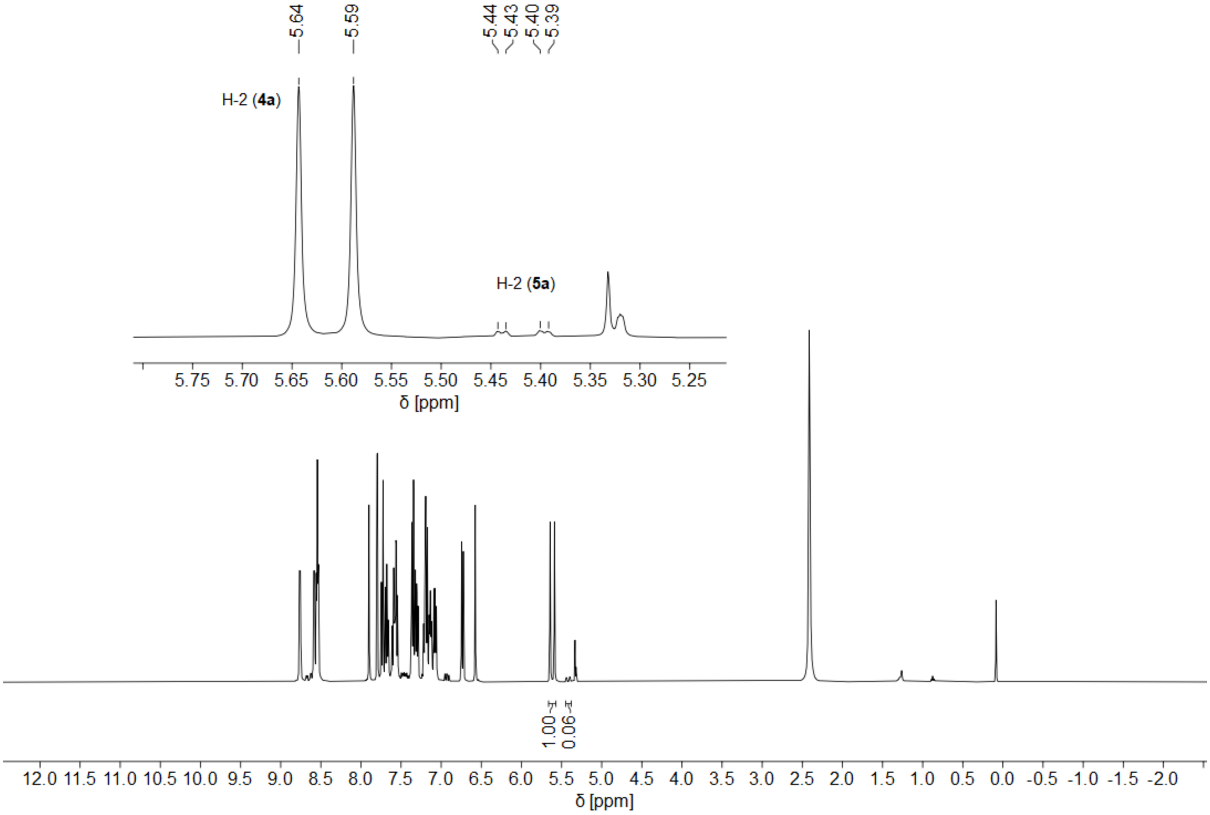

Figure S2.13: ^1H NMR spectrum (CD_2Cl_2) of **4a**/**5a**, zoomed-in on the H-2 signals of both diastereomers.

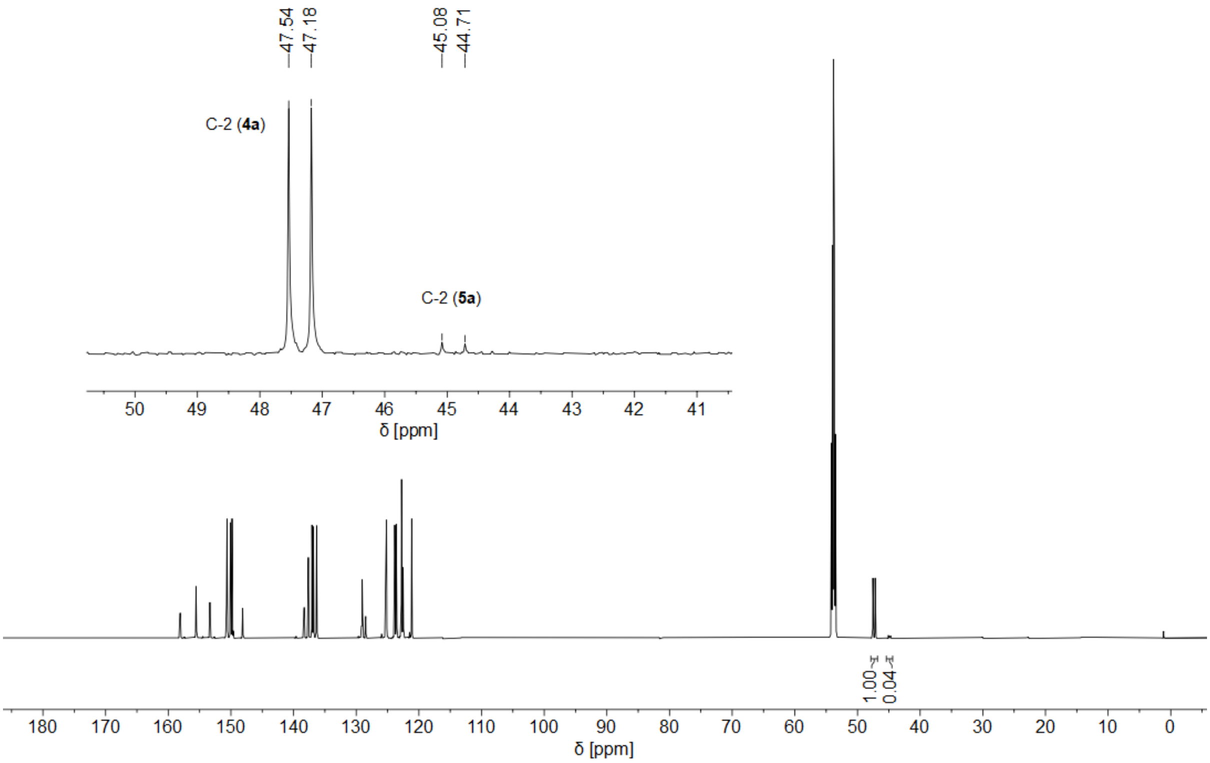

Figure S2.14: $^{13}C\{^{1}H\}$ NMR spectrum (CD_2Cl_2) of **4a**/**5a**, zoomed-in on the C-2 signals of both diastereomers.

(1S,2S)-2,3,5,6-Tetrakis(5-methylpyridin-2-yl)-anti-1*H*,2*H*-dihydrophosphinine 1-oxide (4b)

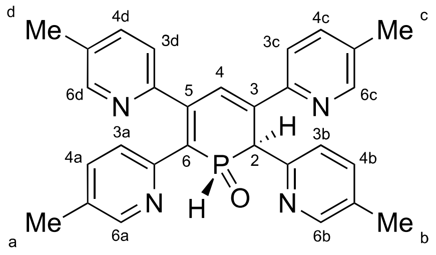

4b

$C_{29}H_{27}N_4OP$
M = 478.54 g/mol

1,2-dihydrophosphinine oxide **4b** was synthesised following the general procedure using phosphinine **2b** (20 mg, 43 µmol, 1.0 equiv) and H_2O (8.0 µL, 0.43 mmol, 10 equiv). The product was obtained as a dark red powder (18 mg, 38 µmol, 89%) in a mixture of 97:3 with the syn-product **5b**.

^{1}H NMR (600 MHz, CD_2Cl_2): δ = 8.58 (d, $^{4}J_{H\text{-}6b,H\text{-}4b}$ = 2.3 Hz, 1H, H-6b), 8.39 (d, $^{4}J_{H\text{-}6c,H\text{-}4c}$ = 2.3 Hz, 1H, H-6c), 8.37 (s, 1H, H-6d), 8.37 (d, $^{4}J_{H\text{-}6a,H\text{-}4a}$ = 2.5 Hz, 1H, H-6a), 7.75 (d, $^{4}J_{H\text{-}4,P}$ = 1.8 Hz, 1H, H-4), 7.59 (d, $^{3}J_{H\text{-}3d,H\text{-}4d}$ = 8.2 Hz, 1H, H-3d), 7.46 (dd, $^{3}J_{H\text{-}4d,H\text{-}3d}$ = 8.1 Hz, $^{4}J_{H\text{-}4d,H\text{-}6d}$ = 2.3 Hz, 1H, H-4d), 7.36 (appt. td, $^{3}J_{H\text{-}4a/H\text{-}4b,H\text{-}3a/H\text{-}3b}$ = 7.8 Hz, $^{4}J_{H\text{-}4a/H\text{-}4b,H\text{-}6a/H\text{-}6b}$ = 2.3 Hz, 2H, H-4a/H-4b), 7.18 (appt. dd, $^{3}J_{H\text{-}3b,H\text{-}4b}$ = 8.1 Hz, 1H, H-3b), 7.16 (d, $^{2}J_{H\text{-}P,P}$ = 527 Hz, 1H, H-P), 7.15 (dd, $^{3}J_{H\text{-}4c,H\text{-}3c}$ = 8.2 Hz, $^{4}J_{H\text{-}4c,H\text{-}6c}$ = 2.3 Hz, 1H, H-4c), 7.06 (d, $^{3}J_{H\text{-}3a,H\text{-}4a}$ = 8.0 Hz, 1H, H-3a), 6.62 (d, $^{3}J_{H\text{-}3c,H\text{-}4c}$ = 8.1 Hz, 1H, H-3c), 5.50 (d, $^{2}J_{H\text{-}6,P}$ = 22 Hz, 1H, H-2), 2.36 (s, 3H, Me-a), 2.29 (s, 3H, Me-d) 2.22 (s, 6H, Me-b/Me-c) ppm.

^{13}C{^{1}H} NMR (151 MHz, CD_2Cl_2): δ = 155.4 (d, $^{3}J_{C\text{-}2d,P}$ = 14 Hz, 1C, C-2d), 153.0 (s, 1C, C-2b), 152.9 (d, $^{3}J_{C\text{-}2c,P}$ = 14 Hz, 1C, C-2c), 151.1 (s, 1C, C-6a), 150.9 (s, 1C, C-6b), 150.4 (s, 1C, C-6c), 150.3 (s, 1C, C-6d), 150.2 (s, 1C, C-3), 147.7 (s, 1C, C-2a), 138.2 (s, 1C, C-4b), 137.5 (s, 1C, C-4d), 137.4 (s, 1C, C-4a), 136.9 (s, 1C, C-4c), 133.9 (s, 1C, C-5a), 133.7 (s, 1C, C-5d), 132.7 (s, 1C, C-5c), 132.6 (s, 1C, C-5b), 128.4 (d, $^{3}J_{C\text{-}4,P}$ = 17 Hz, 1C, C-4), 128.0 (s, 1C, C-6), 127.5 (s, 1C, C-5), 124.7 (s, 2C, C-3c/C-3a), 122.0 (s, 1C, C-3b), 120.7 (s, 1C, C-3d), 46.9 (d, $^{2}J_{C\text{-}6,P}$ = 64 Hz, 1C, C-2), 18.5 (s, 1C, Me-a), 18.4 (s, 2C, Me-c/Me-d), 18.1 (s, 1C, Me-b) ppm.

^{31}P NMR (162 MHz, CD_2Cl_2): 21.1 (dd, $^{1}J_{P,H\text{-}P}$ = 528 Hz, $^{2}J_{P,H\text{-}2}$ = 22.0 Hz) ppm.

^{31}P{^{1}H} NMR (162 MHz, CD_2Cl_2): δ = 21.1 (s) ppm.

IR (ATR): $\tilde{\nu}$ = 3428 (w), 3001 (w), 2921 (w), 1564 (m), 1474 (vs), 1259 (m), 1198 (s), 1028 (s), 828 (s), 804 (s), 705 (m) cm^{-1}.

HRMS-ESI: *m/z* calculated for $C_{29}H_{27}N_4OPNa^+$ [M+Na]$^+$: 501.1815. Found: 501.1843.

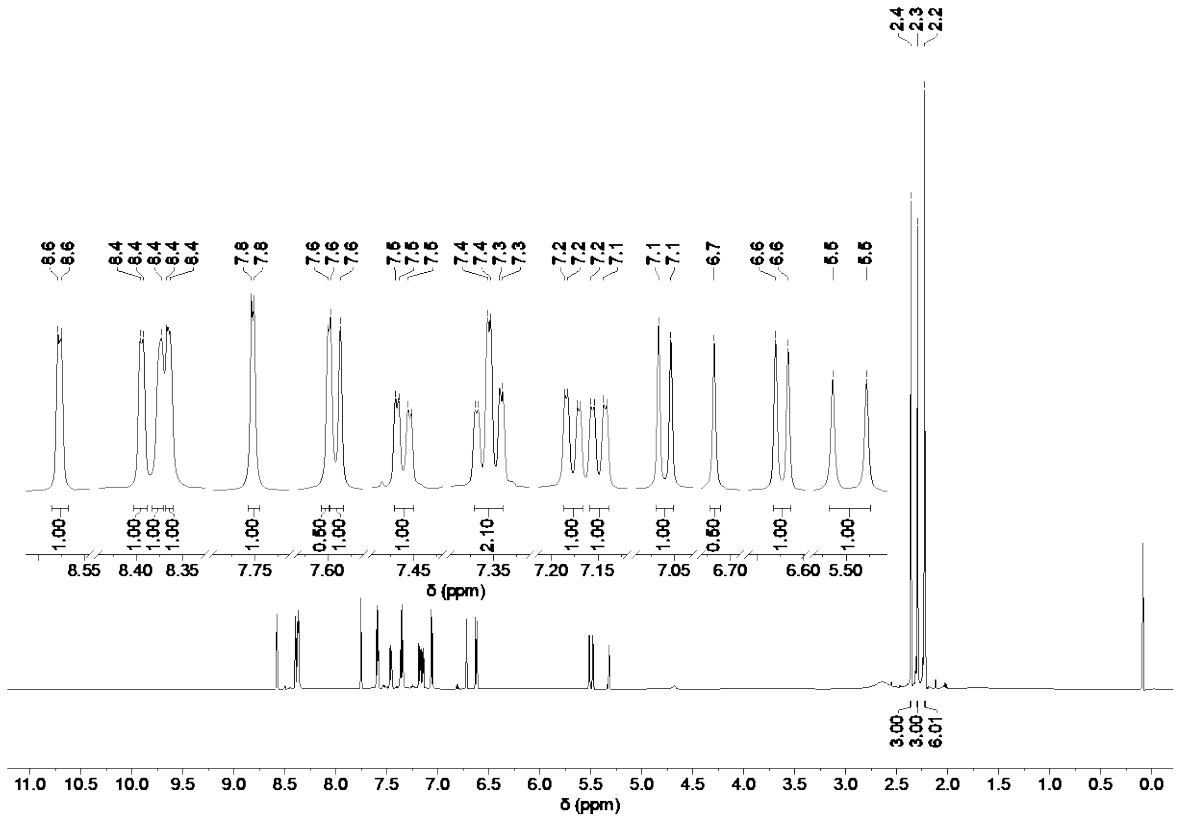

Figure S2.15: ^{1}H NMR spectrum (CD_2Cl_2) of **4b**.

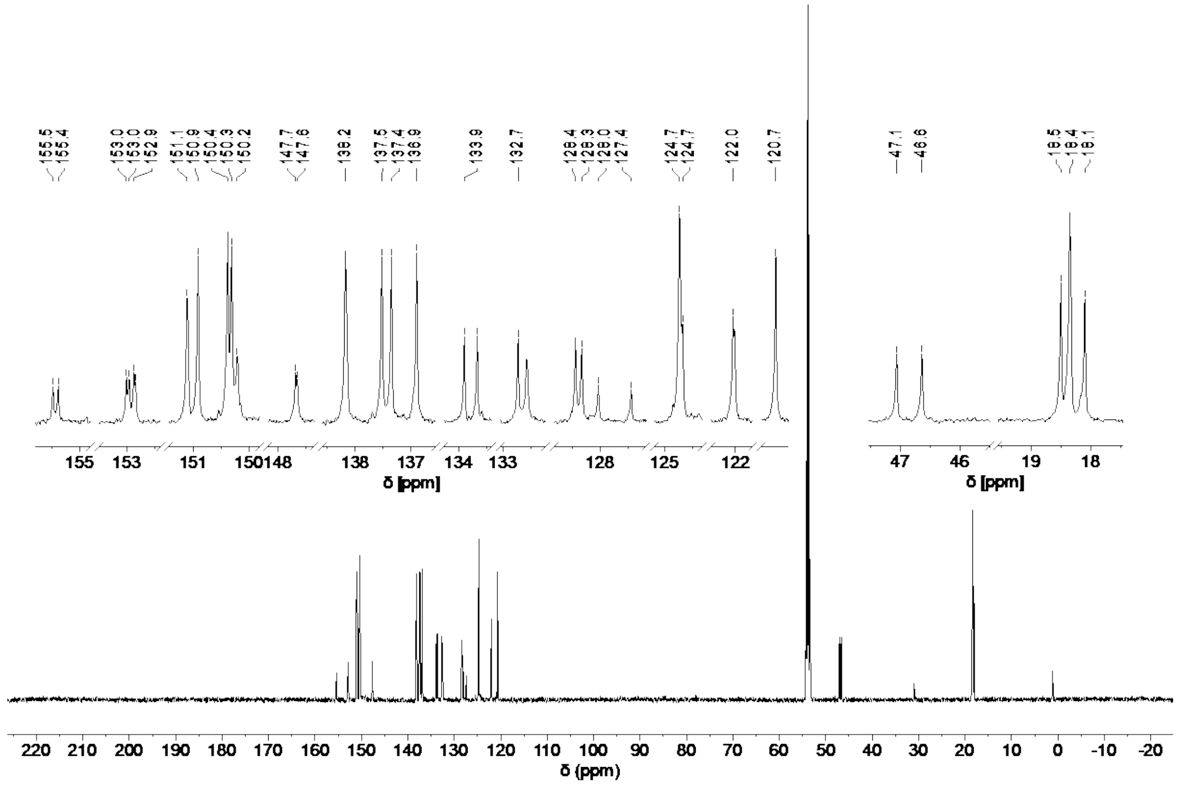

Figure S2.16: $^{13}C\{^{1}H\}$ NMR spectrum (CD_2Cl_2) of **4b**.

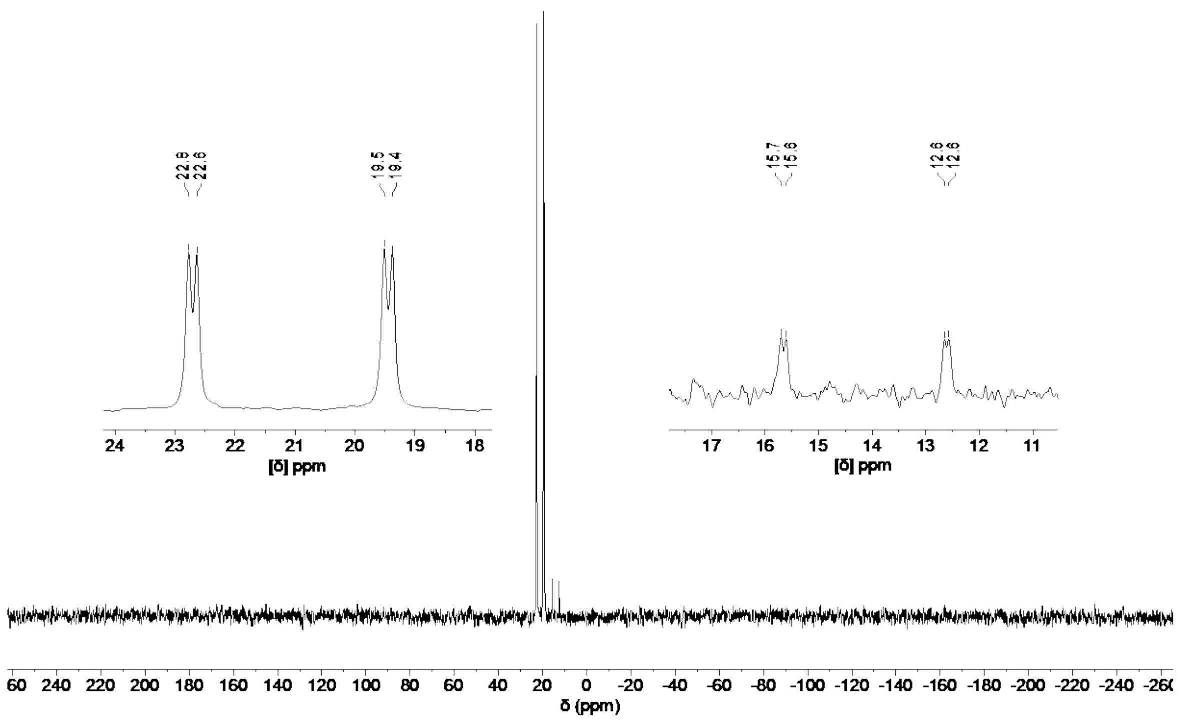

Figure S2.17: ^{31}P NMR spectrum (CD_2Cl_2) of **4b** and **5b**.

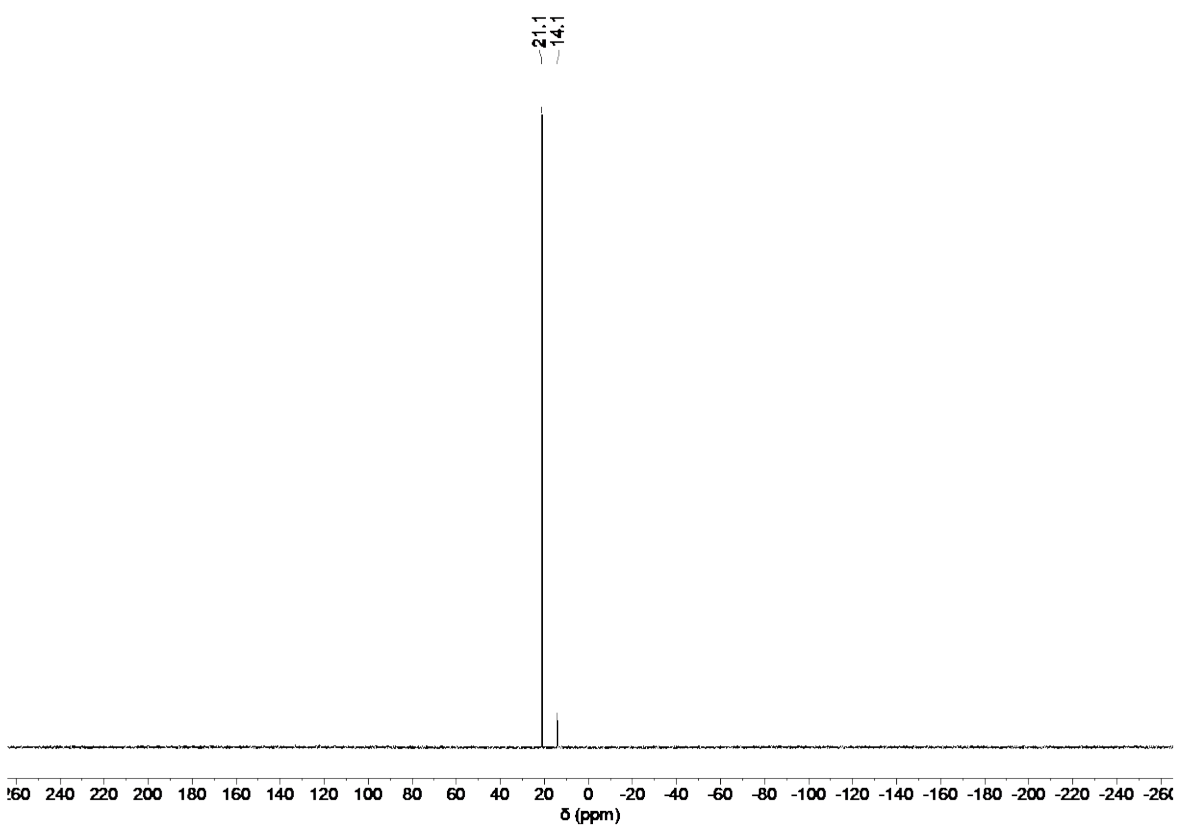

Figure S2.18: ^{31}P{^{1}H} NMR spectrum (CD_2Cl_2) of **4b**.

(1S,2S)-2,3,5,6-Tetrakis(5-methylpyiridin-2-yl)-syn-1*H*,2*H*-dihydrophosphinine 1-oxide (5b)

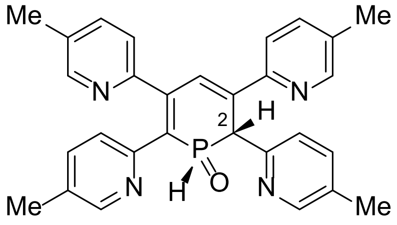

5b

$C_{29}H_{27}N_4OP$
M = 478.54 g/mol

Phosphinine 1-oxide **5b** was not isolated, but obtained as a diastereomeric mixture with its anti-diastereomer in a ratio of 97:3 (anti:syn). Its structure was confirmed by thorough analysis of the ^{1}H and ^{31}P NMR spectra obtained for **4b** (**Figures S2.15/17**). **5b** could not be completely characterized by NMR spectroscopy, as most of its signals overlap with the ones of **5a**, but the assignable signals are listed below.

^{1}H NMR (400 MHz, CD_2Cl_2): δ = 2.31 (s, 3H, Me), 2.29 (s, 3H, Me), 2.24 (s, 3H, Me) ppm.

^{31}P NMR (162 MHz, CD_2Cl_2): δ = 14.1 (dd, $^{1}J_{P,H\text{-}P}$ = 493 Hz, $^{2}J_{P,H\text{-}2}$ = 13.1 Hz) ppm.

^{31}P{^{1}H} NMR (162 MHz, CD_2Cl_2): δ = 14.1 (s) ppm.

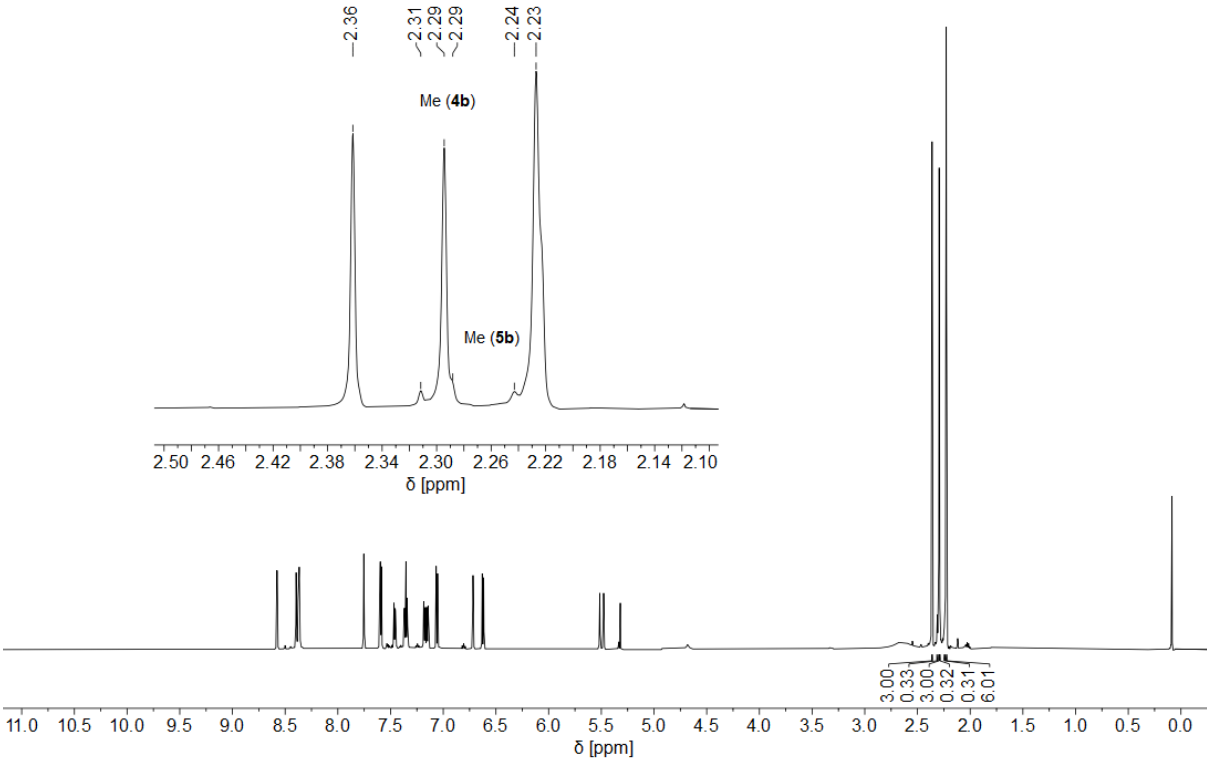

Figure S2.19: ^{1}H NMR spectrum (CD_2Cl_2) of **4a**/**5a**, zoomed-in on the methyl signals of both diastereomers.

2.4 Deprotonation Studies

General Procedure for the Synthesis of 6b

1,2-Dihydrophosphinine oxide **4b** was freshly prepared, and the sample were thoroughly dried as described above. **4b** (43 µmol, 1.0 equiv) was redissolved in CD_2Cl_2 (0.60 mL) and the respective investigated base (10 equiv: pyridine, imidazole, isopropylamine, piperidine, NEt_3; 2.0 equiv: KHMDS) was added at room temperature. The reactions with *n*BuLi and LDA (1.0 equiv) were conducted at −78 °C. The deprotonations using piperidine, LDA and *n*BuLi proceeded instantly to the desired product, as indicated by a colour change from light yellow to dark red (piperidine) or fluorescent orange (LDA, *n*BuLi).

Caution: Reactions of organolithium reagents in dichloromethane should be handled with care. The former can induce α-elimination of the solvent, yielding carbenoids (α-haloorganolithium compounds), which can subsequently generate the corresponding, highly thermolabile, carbenes.[14] For reactions on larger scales or with excesses of the organolithium reagent, the use of dichloromethane or chloroform should therefore be avoided and substituted for more suitable solvents (e.g. *n*-pentane, *n*-hexane, benzene,…).

2,3,5,6-Tetrakis(pyridin-2-yl)-λ⁵-phosphinin-1-olate-tetramer (6a(Li))

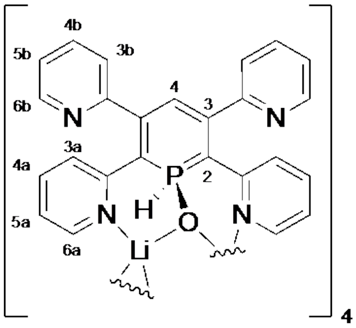

6a(Li)

$C_{100}H_{68}Li_4N_{16}O_4P_4$
M = 1713.44 g/mol

For the synthesis of **6a(Li)** a solution of **4a** (57 mg, 0.13 mmol) in CH_2Cl_2 (10 mL) was freshly prepared in a 10 mL Schlenkflask. At room temperature under vigorous stirring, MeLi (1.5 M in Et_2O, 0.10 mL, 0.15 mmol, 1.1 equiv) was added. Immediately after addition, the colour changed to fluorescent orange, with an orange, fluorescent solid precipitating. The solvent was removed *in vacuo*, yielding **6a(Li)** (38 mg, 22 µmol, 65%).

Unfortunately, a suitable solvent to redissolve the obtained product could not be found. A full NMR characterization could therefore not be performed, and the identity of the title compound was solely confirmed by ^{31}P NMR spectroscopy. Similar resonances in the ^{31}P NMR spectrum

were found for compound **6b(Li)**. The product's nature and connectivity could then however be confirmed *via* X-ray diffraction. Suitable crystals for single crystal XRD were obtained as orange, fluorescent needles from a saturated solution of **6a(Li)** in CH_2Cl_2.

^{31}P NMR (162 MHz, CD_2Cl_2): δ = 9.7 (d, $^1J_{P,H\text{-}P}$ = 529 Hz) ppm.

^{31}P{^{1}H} NMR (162 MHz, CD_2Cl_2): δ = 9.7 (s) ppm.

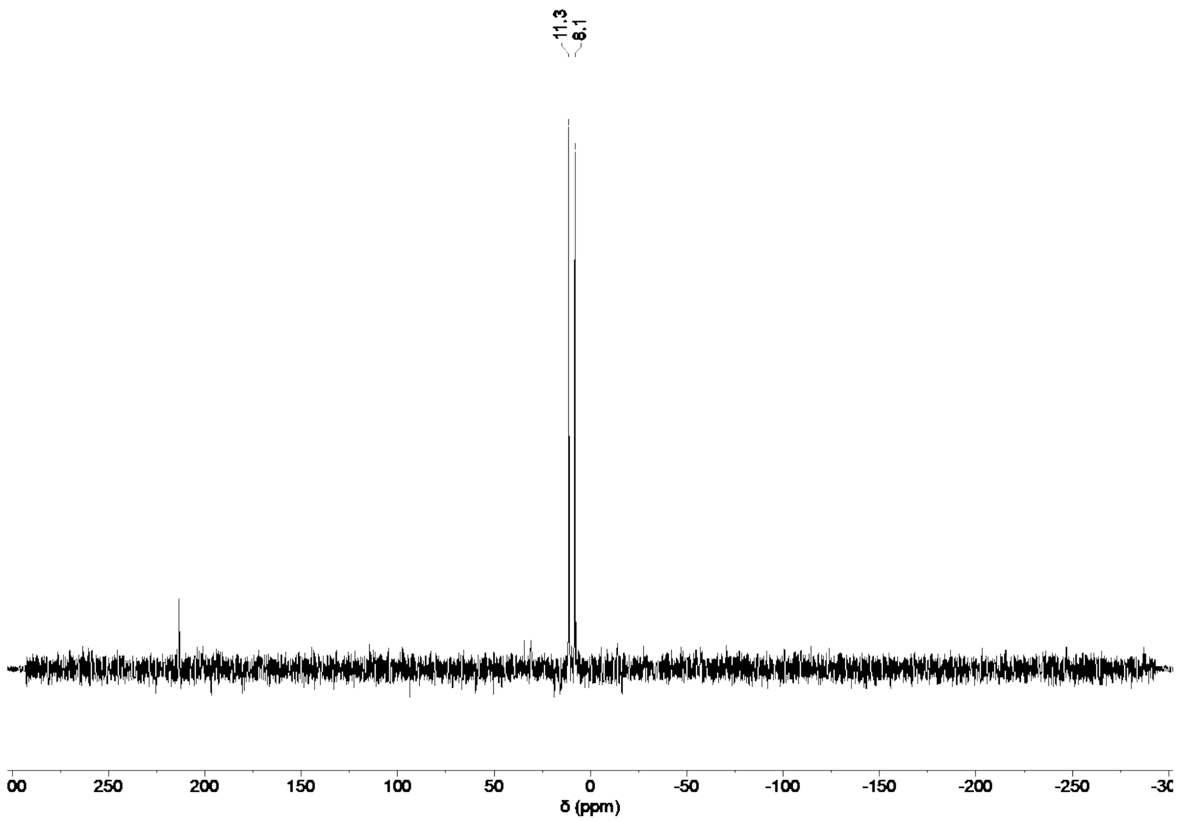

Figure S2.20: ^{31}P NMR spectrum (CD_2Cl_2) of **6a(Li)**.

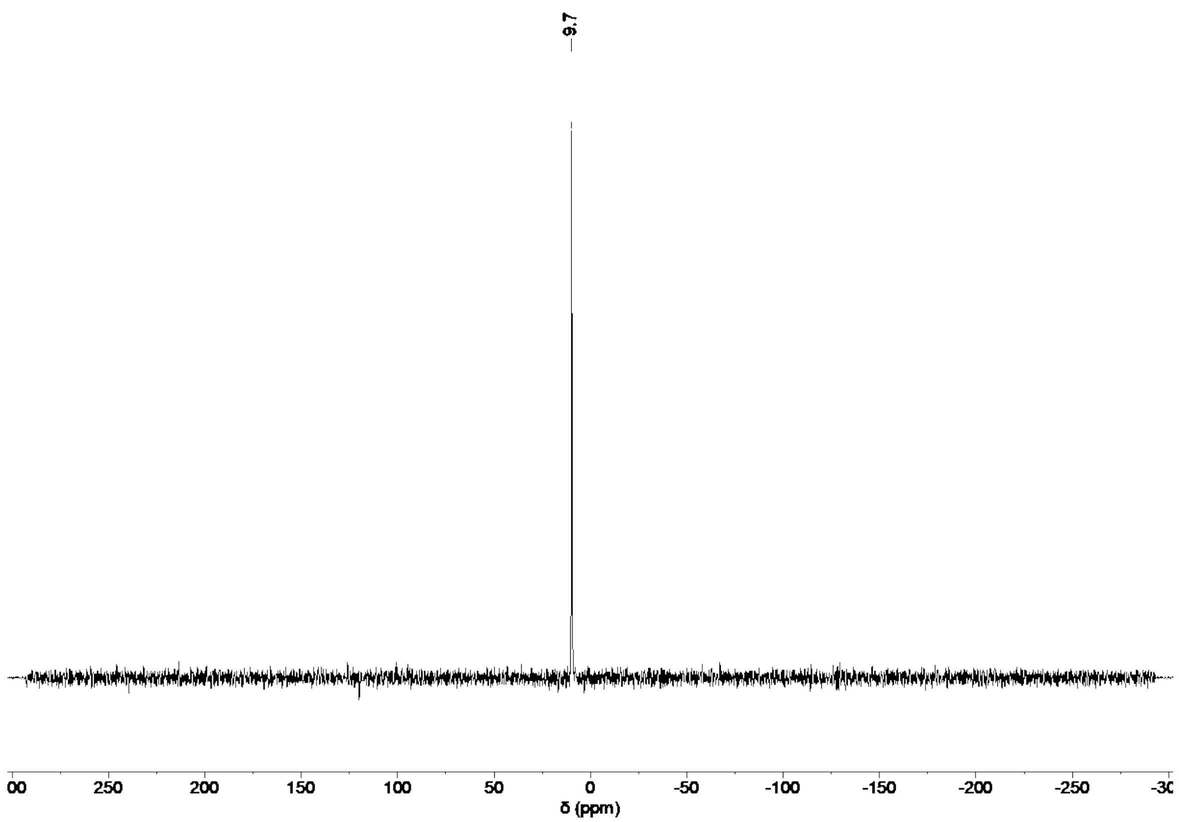

Figure S2.21: ${}^{31}P\{{}^{1}H\}$ NMR spectrum (CD_2Cl_2) of **6a(Li)**.

2,3,5,6-Tetrakis(5-methylpyridin-2-yl)-λ⁵-phosphinin-1-olate (6b(Li))

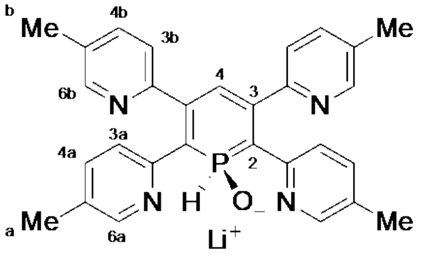

6b(Li)

$C_{29}H_{26}LiN_4OP$
M = 484.47 g/mol

The lithium salt of λ^5-phosphinin-1-olate **6b** was prepared after the general procedure above, using *n*BuLi (1.6 M in hexanes, 30 µL, 47 µmol, 1.1 equiv.) at −78 °C. After the sample was allowed to warm up to room temperature, its colour had changed from pale yellow to a fluorescent orange. The solvents were removed *in vacuo* and the resulting solid was thoroughly dried. The product was obtained as a bright orange powder (19 mg, 39 µmol, 90%). Unfortunately, a suitable solvent to redissolve the lithium salt after drying was not found. The product was therefore characterised directly from the reaction mixture, explaining the impurities visible in the ${}^{1}H$ and ${}^{13}C\{{}^{1}H\}$ NMR spectra. With the aid of ${}^{1}H/{}^{1}H$ COSY and

$^{1}H/^{13}C$ HMQC NMR spectra, the product resonances could however still be completely assigned.

^{1}H NMR (600 MHz, CD_2Cl_2): δ = 8.54 (appt. s, 4H, H-6a/6b), 7.83 (d, $^{1}J_{H\text{-}2,P''}$ = 552 Hz, 1H, H-2), 6.95 (d, $^{3}J_{H\text{-}4',H\text{-}3'}$ = 8.3 Hz, 2H, H-4a), 6.71 (d, $^{3}J_{H\text{-}4'',H\text{-}3''}$ = 7.8 Hz, 2H, H-4b), 6.51 (d, $^{3}J_{H\text{-}3',H\text{-}4'}$ = 8.2 Hz, 2H, H-3a), 6.26 (s, 1H, H-4), 5.79 (d, $^{3}J_{H\text{-}3'',H\text{-}4''}$ = 7.9 Hz, 2H, H-3b), 2.34 (s, 6H, Me-b), 1.79 (s, 6H, Me-a) ppm.

^{13}C{^{1}H} NMR (151 MHz, CD_2Cl_2): δ = 158.4 (s, 2C, C-2a), 157.8 (s, 2C, C-2b), 149.9 (s, 2C, C-6b), 149.2 (s, 2C, C-6a), 134.9 (s, 2C, C-3), 135.9 (s, 2C, C-4a), 135.3 (s, 2C, C-4b), 131.1 (s, 2C, C-5b), 128.1 (s, 2C, C-5a), 125.0 (s, 2C, C-3b), 124.7 (s, 2C, C-3a), 108.3 (d, $^{3}J_{C\text{-}4,P}$ = 12.8 Hz, 1C, C-4), 100.2 (d, $^{1}J_{C\text{-}2,P}$ = 113 Hz, 1C, C-2), 18.0 (s, 2C, Me-b), 17.8 (s, 2C, C-Me-a) ppm.

^{31}P NMR (162 MHz, CD_2Cl_2): δ = 7.7 (d, $^{1}J_{P,H\text{-}P}$ = 527 Hz) ppm.

^{31}P{^{1}H} NMR (162 MHz, CD_2Cl_2): δ = 7.7 (s) ppm.

HRMS-ESI: *m/z* calculated for $C_{29}H_{26}N_4OP^-$ [M−Li]$^-$: 477.1849. Found: 477.1770.

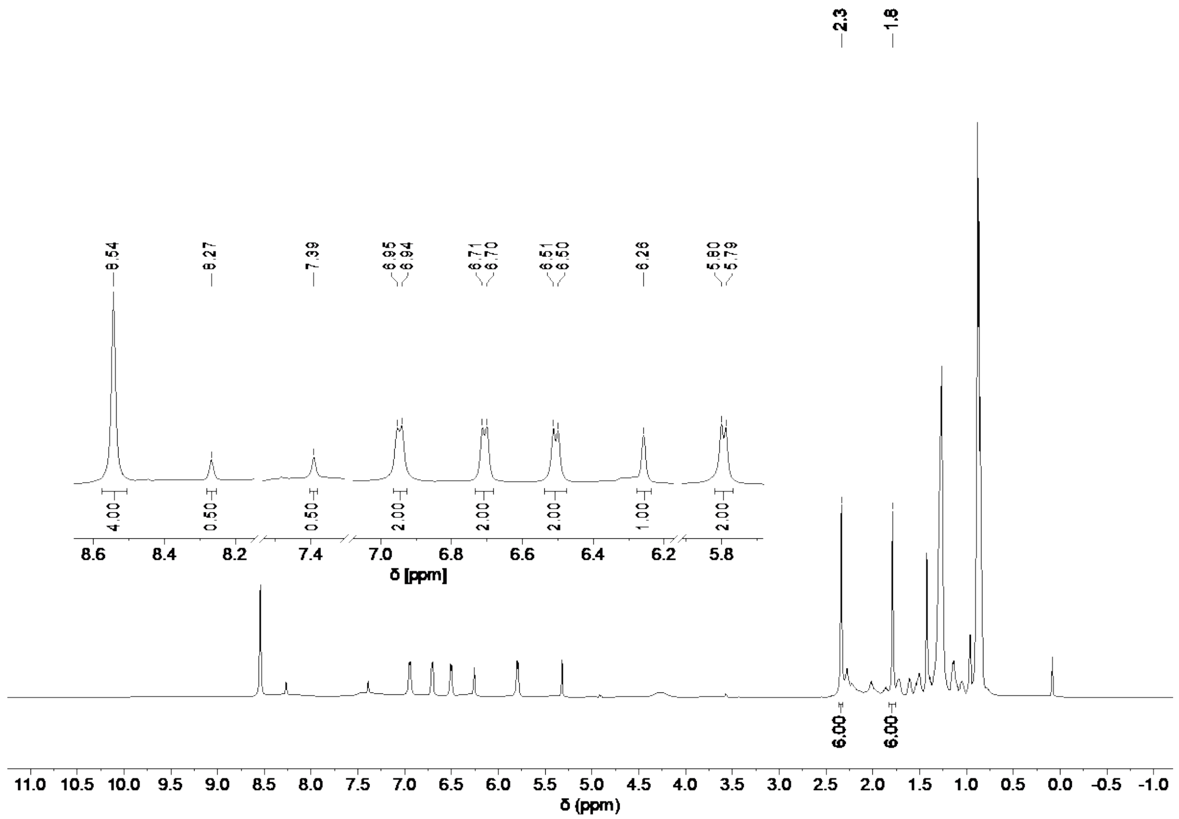

Figure S2.22: ^{1}H NMR spectrum (CD_2Cl_2) of **6b**.

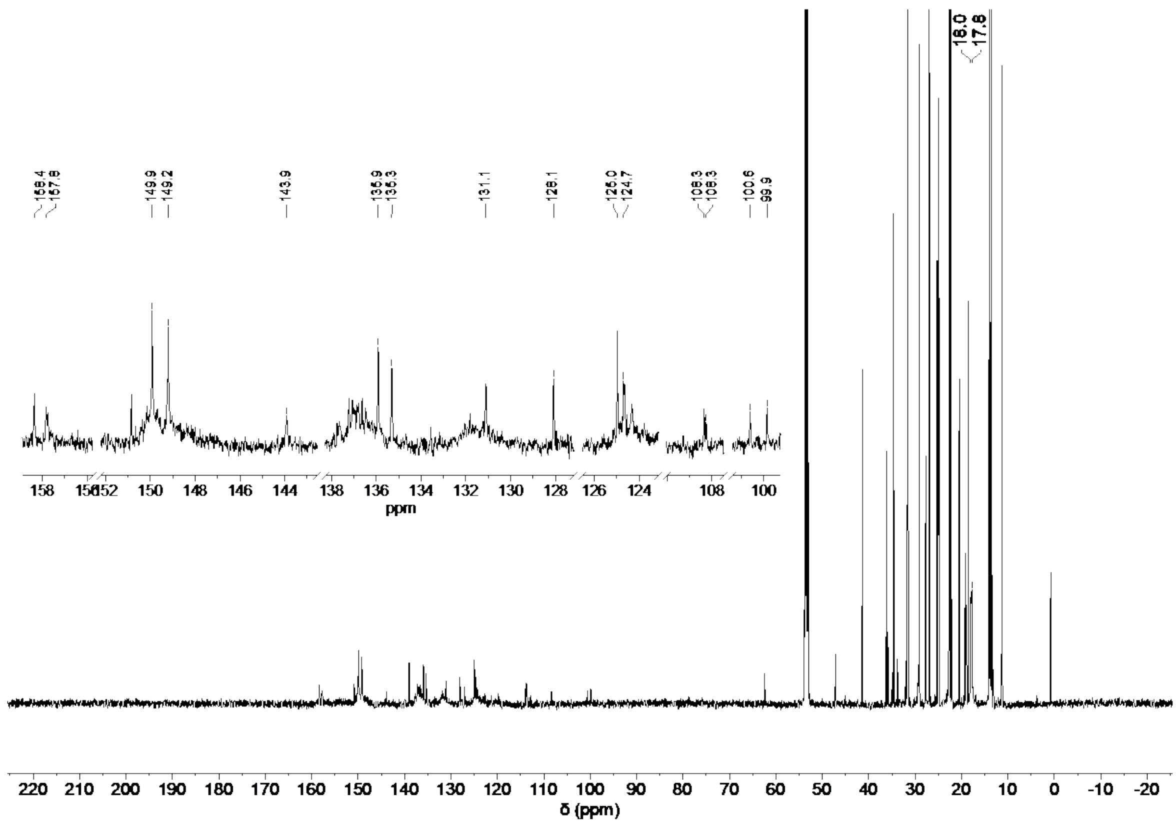

Figure S2.23: $^{13}C\{^{1}H\}$ NMR spectrum (CD_2Cl_2) of **6b**.

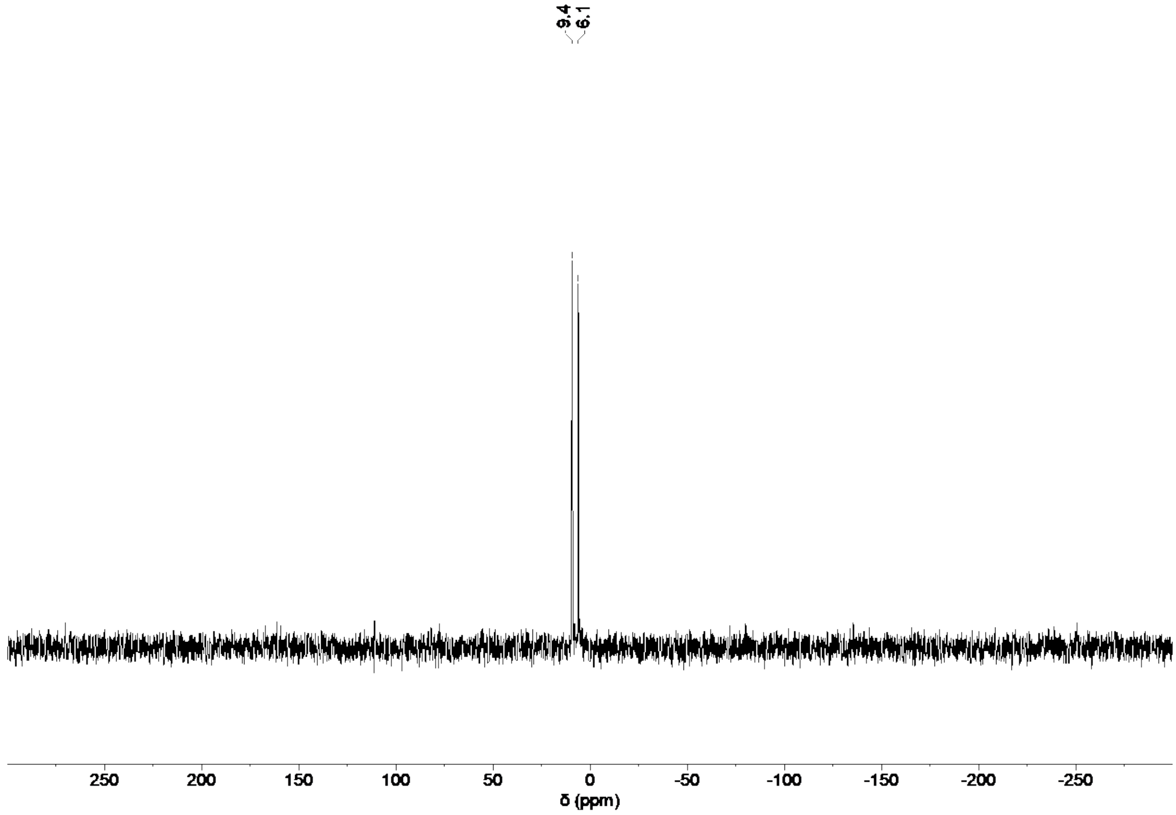

Figure S2.24: ^{31}P NMR spectrum (CD_2Cl_2) of **6b**.

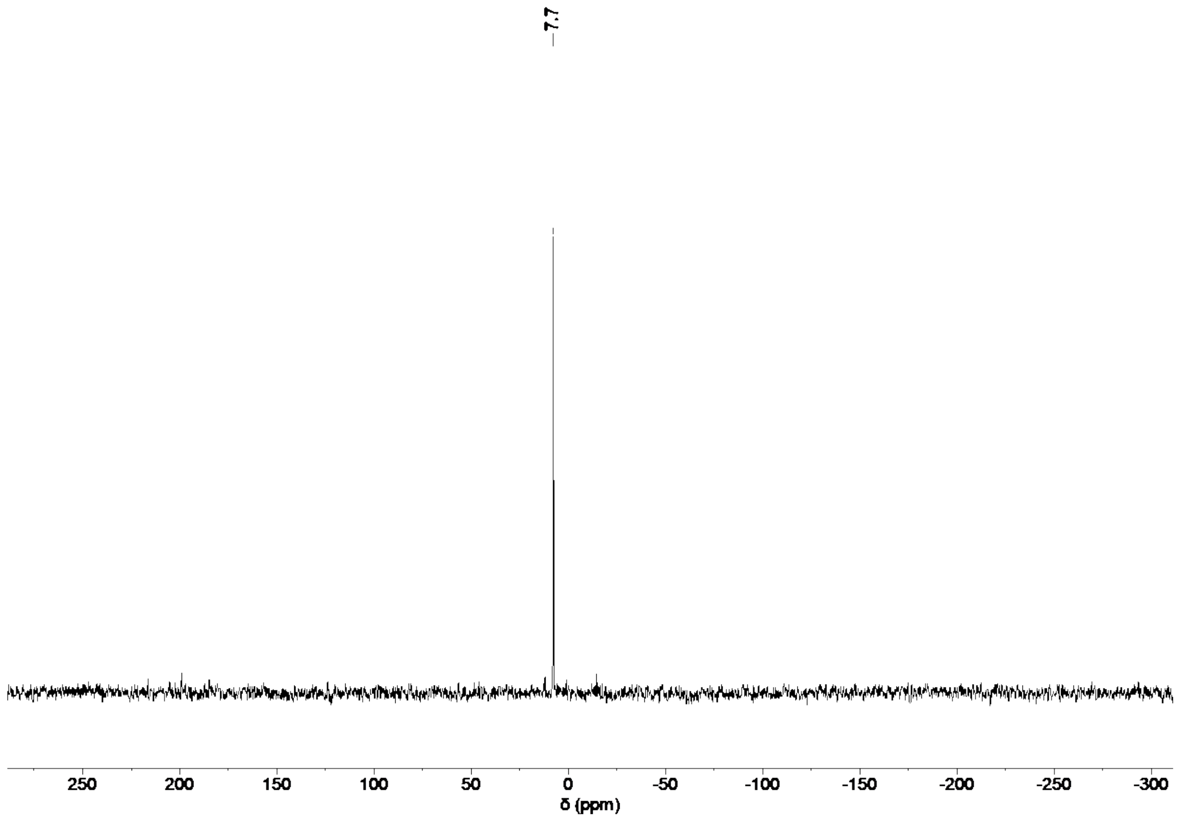

Figure S2.25: $^{31}P\{^{1}H\}$ NMR spectrum (CD_2Cl_2) of **6b**.

2.5 Coordination Chemistry of Cu(I)

Di(2,3,5,6-Tetrakis(pyridin-2-yl)phosphinine-copper-iodide) complex (7a)

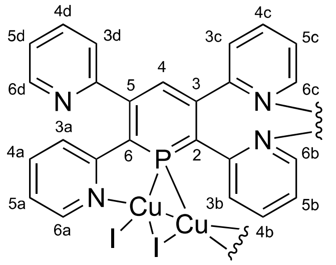

7a

$C_{50}H_{34}Cu_4I_4N_8P_2$
M = 1570.63 g/mol

In a 20 mL Schlenk flask, phosphinine **2a** (95mg, 0.24 mmol) was dissolved in 8.0 mL of dried and degassed CH_2Cl_2. To the colourless solution, $CuI\cdot SMe_2$ (0.11 g, 0.45 mmol, 1.9 equiv) was added at rt. The colour changed immediately to an intense red. After 30 min at rt, the remaining precipitate was filtered off. After removing the solvent *in vacuo* to dry the product, no suitable solvent for NMR spectroscopic analysis to re-dissolve the obtained product could be found. The product was obtained as a dark red crystalline solid (0.18 g, 0.11 mmol, 95%).

To circumvent the solubility issue, the reaction was repeated in a J. Young NMR tube with CD_2Cl_2 (500 µL) and the obtained reaction mixture was directly used for all further characterization. Suitable crystals for single crystal XRD were obtained by diffusion of *n*-pentane into a saturated solution of **7a** in CH_2Cl_2.

^{1}H NMR (400 MHz, CD_2Cl_2): δ = 8.95 (ddd, $^3J_{H\text{-}6b\text{-}H\text{-}5b}$ = 5.5 Hz, $^4J_{H\text{-}6b\text{-}H\text{-}4b}$ = 1.8 Hz, $^5J_{H\text{-}6b\text{-}H3b}$ = 0.8 Hz, 1H, H-6b), 8.77 (ddd, $^3J_{H\text{-}6d\text{-}H\text{-}5d}$ = 5.4 Hz, $^4J_{H\text{-}6d\text{-}H\text{-}4d}$ = 1.7 Hz, $^5J_{H\text{-}6d\text{-}H\text{-}3d}$ = 0.9 Hz, 1H, H-6d), 8.49 (ddd, $^3J_{H\text{-}3c\text{-}H\text{-}4c}$ = 7.9 Hz, $^4J_{H\text{-}3c\text{-}H\text{-}5c}$ = 1.1 Hz, $^5J_{H\text{-}3c\text{-}H\text{-}6c}$ = 1.1 Hz, 1H, H-3c), 8.38 (ddd, $^3J_{H\text{-}3a\text{-}H\text{-}5a}$ = 5.5 Hz, $^4J_{H\text{-}6a\text{-}H\text{-}4a}$ = 1.8 Hz, $^5J_{H\text{-}6a\text{-}H\text{-}3a}$ = 0.8 Hz, 1H, H-6a), 8.15 (ddd, $^3J_{H\text{-}3c\text{-}H\text{-}5c}$ = 5.2 Hz, $^4J_{H\text{-}6c\text{-}H\text{-}4c}$ = 1.8 Hz, $^5J_{H\text{-}6c\text{-}H\text{-}3c}$ = 0.9 Hz, 1H, H-6c), 8.08 (ddd, $^3J_{H\text{-}3d\text{-}H\text{-}4d}$ = 8.1 Hz, $^4J_{H\text{-}3d\text{-}H\text{-}5d}$ = 1.1 Hz, $^5J_{H\text{-}3d\text{-}H\text{-}6d}$ = 1.1 Hz, 1H, H-3d), 7.89 (ddd, $^3J_{H\text{-}4c\text{-}H\text{-}3c}$ = 7.8 Hz, $^3J_{H\text{-}4c\text{-}H\text{-}5c}$ = 7.8 Hz, $^4J_{H\text{-}4c\text{-}H\text{-}6c}$ = 1.8 Hz, 1H, H-4c), 7.60 (ddd, $^3J_{H\text{-}4b\text{-}H\text{-}3b}$ = 7.7 Hz, $^3J_{H\text{-}4b\text{-}H\text{-}5b}$ = 7.7 Hz, $^4J_{H\text{-}4b\text{-}H\text{-}6b}$ = 1.8 Hz, 1H, H-4b), 7.48 (ddd, $^3J_{H\text{-}4a\text{-}H\text{-}3a}$ = 7.7 Hz, $^3J_{H\text{-}4a\text{-}H\text{-}5a}$ = 7.7 Hz, $^4J_{H\text{-}4a\text{-}H\text{-}6a}$ = 1.8 Hz, 1H, H-4a), 7.30 (ddd, $^3J_{H\text{-}4d\text{-}H\text{-}3d}$ = 8.1 Hz, $^3J_{H\text{-}4d\text{-}H\text{-}5d}$ = 7.9 Hz, $^4J_{H\text{-}4d\text{-}H\text{-}6d}$ = 1.8 Hz, 1H, H-4d), 7.27(ddd, $^3J_{H\text{-}5a\text{-}H\text{-}4a}$ = 7.6 Hz, $^3J_{H\text{-}5a\text{-}H\text{-}6a}$ = 4.8 Hz, $^4J_{H\text{-}5a\text{-}H\text{-}3a}$ = 1.1 Hz, 1H, H-5a), 7.23 (ddd, $^3J_{H\text{-}3b\text{-}H\text{-}4b}$ = 7.8 Hz, $^4J_{H\text{-}3b\text{-}H\text{-}5b}$ = 1.1 Hz, $^5J_{H\text{-}3b\text{-}H\text{-}6b}$ = 1.1 Hz, 1H, H-3b), 7.16 (ddd, $^3J_{H\text{-}5b\text{-}H\text{-}4b}$ = 7.6 Hz, $^3J_{H\text{-}5b\text{-}H\text{-}6b}$ = 5.4 Hz, $^4J_{H\text{-}5b\text{-}H\text{-}3b}$ = 1.3 Hz, 1H, H-5b), 7.11(ddd, $^3J_{H\text{-}5c\text{-}H\text{-}4c}$ = 7.6 Hz, $^3J_{H\text{-}5c\text{-}H\text{-}6c}$ = 5.2 Hz, $^4J_{H\text{-}5c\text{-}H\text{-}3c}$ = 1.2 Hz, 1H, H-5c), 7.09 (d, $^4J_{H\text{-}4\text{-}P}$ = 4.0 Hz, 1H, H-4), 7.04 (ddd, $^3J_{H\text{-}5d\text{-}H\text{-}4d}$ = 7.5 Hz, $^3J_{H\text{-}5d\text{-}H\text{-}6d}$ = 5.3

Hz, $^{4}J_{H\text{-}5d\text{-}H\text{-}3d}$ = 1.2 Hz, 1H, H-5d), 6.95 (ddd, $^{3}J_{H\text{-}3a\text{-}H\text{-}4a}$ = 7.9 Hz, $^{4}J_{H\text{-}3a\text{-}H\text{-}5a}$ = 1.1 Hz, $^{5}J_{H\text{-}3a\text{-}H\text{-}6a}$ = 1.1 Hz, 1H, H-3a).

^{13}C{^{1}H} NMR (151 MHz, CD_2Cl_2): 158.1 (d, $^{3}J_{C\text{-}2b\text{-}P}$ = 4.4 Hz, 1C, C-2b), 156.5 (d, $^{3}J_{C\text{-}2a\text{-}P}$ = 5.1 Hz, 1C, C-2a), 156.1 (s, 1C, C-2d), 155.9 (d, $^{4}J_{C\text{-}2c\text{-}P}$ = 8.1 Hz, 1C, C-2c), 150.8 (d, $^{2}J_{C\text{-}5\text{-}P}$ = 4.1 Hz, 1C, C-5), 150.6 (s, 1C, C-6b), 150.1 (s, 2C, C-6a, C-6d), 150.0 (s, 1C, C-6c), 147.3 (d, $^{1}J_{C\text{-}2\text{-}P}$ = 10.3 Hz, 1C, C-2), 145.3 (d, $^{1}J_{C\text{-}6\text{-}P}$ = 9.4 Hz, 1C, C-6), 143. (d, $^{2}J_{C\text{-}3\text{-}P}$ = 6.2 Hz, 1C, C-3), 138.61 (s, 2C, C-4b, C-4c), 136.2 (s, 1C, C-4d), 136.1 (s, 1C, C-4a), 133.9 (d, $^{3}J_{C\text{-}4\text{-}P}$ = 17.1 Hz, 1C, C-4), 129.0 (d, $^{4}J_{C\text{-}3c\text{-}P}$ = 8.2 Hz, 1C, C-3c), 128.4 (d, $^{4}J_{C\text{-}3d\text{-}P}$ = 5.4 Hz, 1C, C-3d), 126.3 (s, 1C, C-3a), 125.5 (s, 1C, C-3b), 124.01 (s, 1C, C-5c), 123.7 (s, 1C, C-5b), 123.5 (s, 1C, C-5d), 123.4 (s, 1C, C-5a), 19.8 (s, 4C, S(CH_3)$_2$) ppm.

^{31}P NMR (162 MHz, CD_2Cl_2): δ = 154.3 ppm.

HRMS-ESI: *m/z* calculated for $C_{50}H_{34}Cu_3I_2N_8P_2^+$ [(PP)$_2$(Cu_3I_2)]$^+$: 1250.8359 Found: 1250.8249

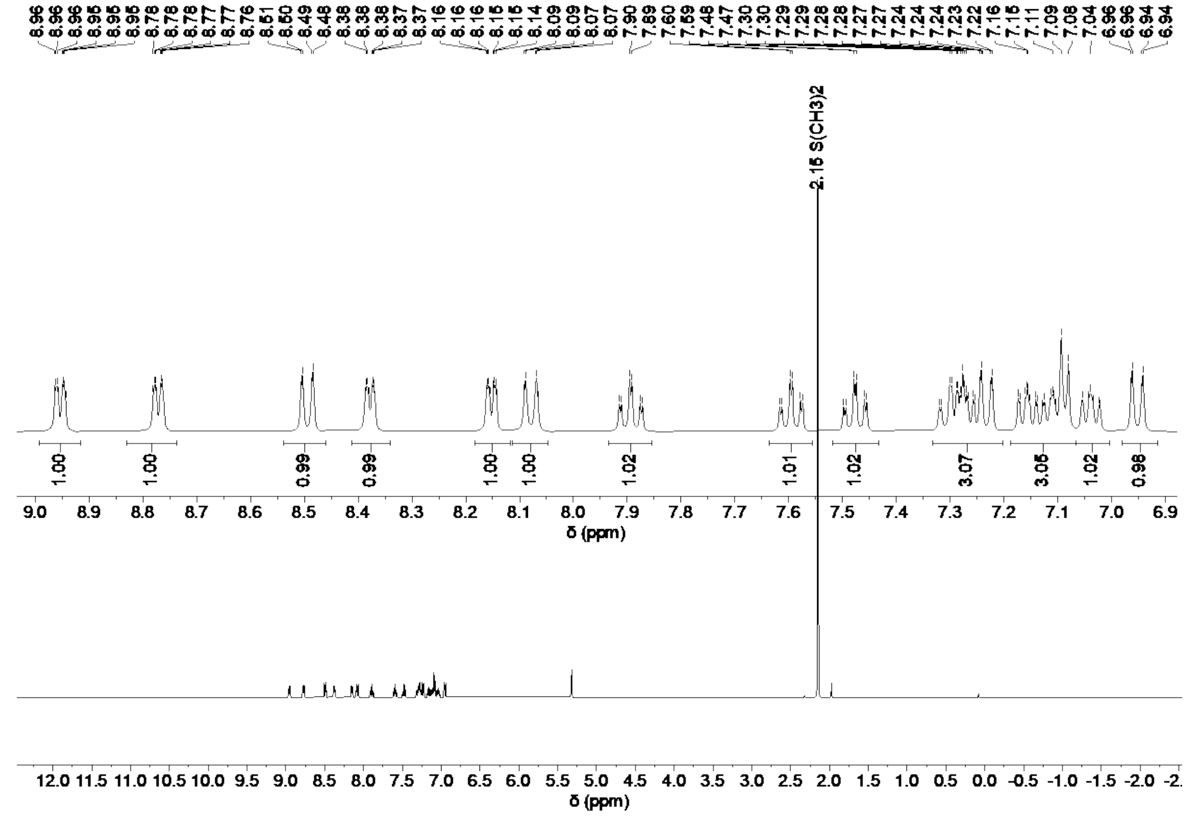

Figure S2.26: ^{1}H NMR spectrum (CD_2Cl_2) of **7a**.

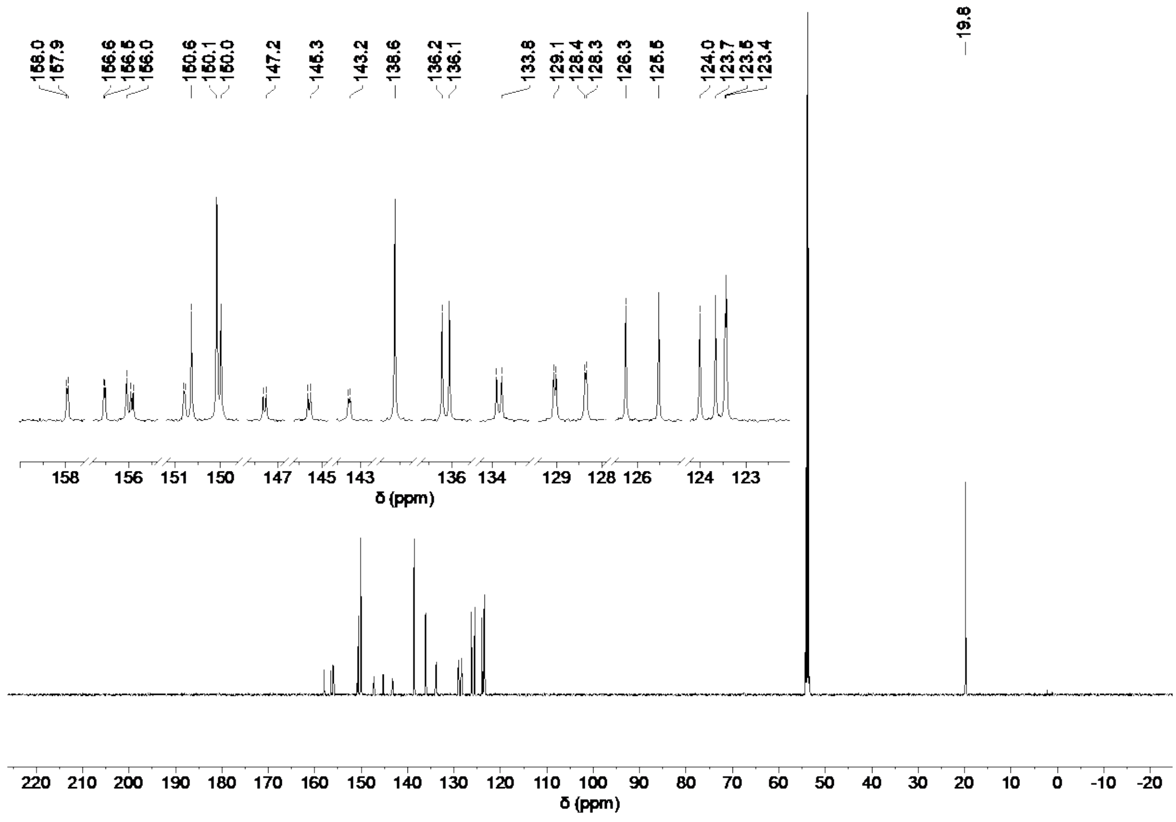

Figure S2.27: $^{13}C\{^{1}H\}$ NMR spectrum (CD_2Cl_2) of **7a**.

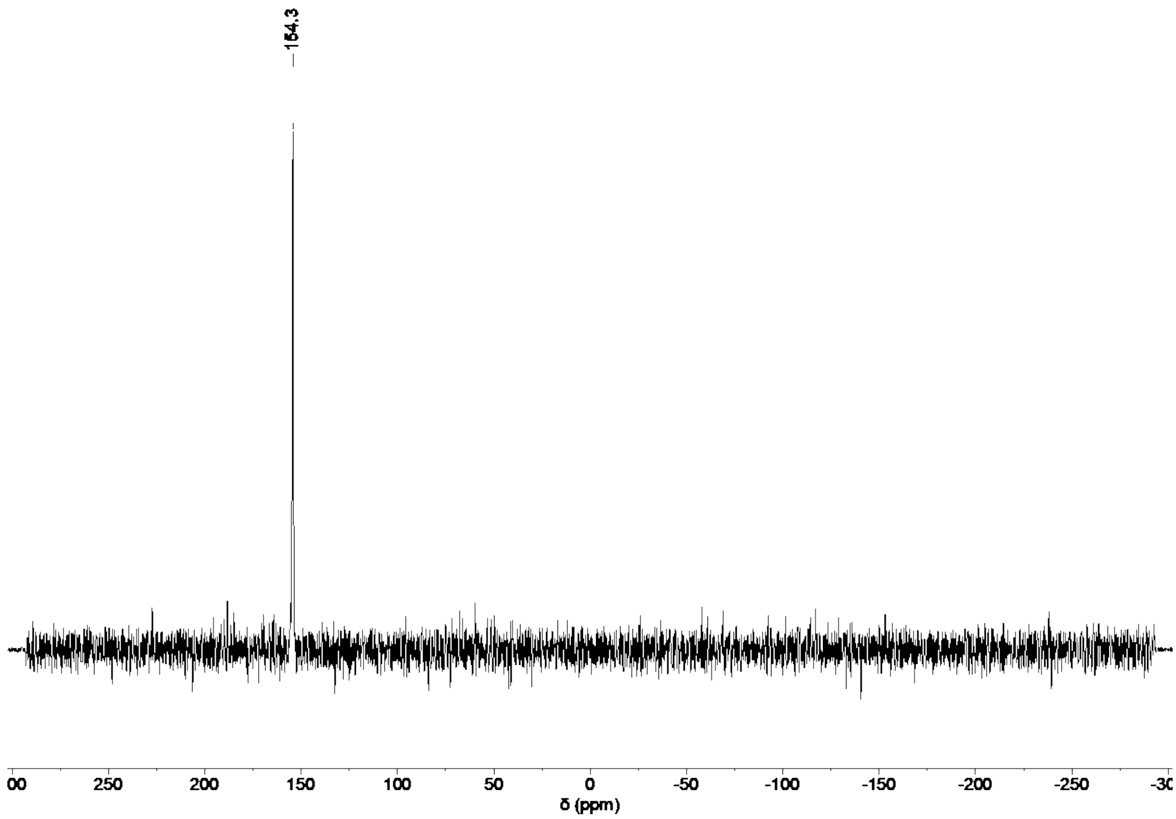

Figure S2.28: ^{31}P NMR spectrum (CD_2Cl_2) of **7a**.

Di(2,3,5,6-Tetrakis(5-methylpyridin-2-yl)phosphinine-copper-iodide) complex (7b)

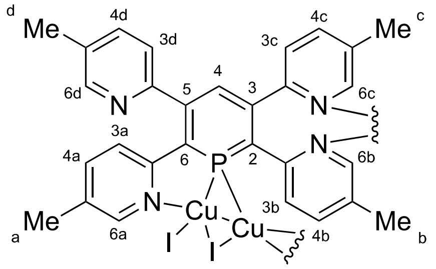

7b

$C_{58}H_{58}Cu_4I_4N_8P_2$
M = 1682.84 g/mol

In a 10 mL Schlenk flask, a solution of phosphinine **2b** (21 mg, 45 µmol) was prepared in 2.0 mL CD_2Cl_2. At room temperature, CuI·SMe_2 (23 mg, 91 µmol, 2.0 eq.) was added to the colourless solution. The colour changed immediately to an intense red. After 2 h at rt, the remaining precipitate was filtered off. After removing the solvent *in vacuo* to dry the product, no suitable solvent for NMR spectroscopic analysis to re-dissolve the obtained product could be found. The product was obtained as a dark red crystalline solid (36 mg, 21 mmol, 93 %)

The NMR characterisation was done directly from the reaction mixture in in a J. Young NMR tube with CD_2Cl_2 (500 µL). and the obtained reaction mixture was directly used for all further characterization. Suitable crystals for single crystal XRD were obtained through diffusion of *n*-pentane into a solution of **7b** in CH_2Cl_2.

^{1}H NMR (700 MHz, CD_2Cl_2): δ = 8.78 (d, $^{4}J_{H\text{-}6a\text{-}H\text{-}4a}$ = 2.8 Hz, 1H, H-6a), 8.60 (d, $^{4}J_{H\text{-}6d\text{-}H\text{-}4d}$ = 2.6 Hz, 1H, H-6d), 8.30 (dd, $^{3}J_{H\text{-}3c\text{-}H\text{-}4c}$ = 8.2 Hz, $^{5}J_{H\text{-}3c\text{-}H\text{-}5c}$ = 2.2 Hz, 1H, H-3c), 8.14 (d, $^{4}J_{H\text{-}6a\text{-}H\text{-}4a}$ = 2.3 Hz, 1H, H-6a), 7.99 (d, $^{4}J_{H\text{-}6c\text{-}H\text{-}4c}$ = 2.6 Hz, 1H, H-6c), 7.97 (dd, $^{3}J_{H\text{-}3d\text{-}H\text{-}4d}$ = 8.4 Hz, ´$^{5}J_{H\text{-}3d\text{-}H\text{-}6d}$ = 3.1 Hz, 1H, H-3d), 7.70 (dd, $^{3}J_{H\text{-}4c\text{-}H\text{-}3c}$ = 8.4 Hz, $^{4}J_{H\text{-}4c\text{-}H\text{-}6c}$ = 2.4 Hz, 1H, H-4c), 7.40 (dd, $^{3}J_{H\text{-}4a\text{-}H\text{-}3a}$ = 8.7 Hz, $^{4}J_{H\text{-}4a\text{-}H\text{-}6a}$ = 2.4 Hz, 1H, H-4a), 7.28 (dd, $^{3}J_{H\text{-}4b\text{-}H\text{-}3b}$ = 8.7 Hz, $^{4}J_{H\text{-}4b\text{-}H\text{-}6b}$ = 2.2 Hz, 1H, H-4b), 7.11 (dd, $^{3}J_{H\text{-}3b\text{-}H\text{-}4b}$ = 7.8 Hz, $^{4}J_{H\text{-}3b\text{-}H\text{-}5b}$ = 1.1 Hz, $^{5}J_{H\text{-}3b\text{-}H\text{-}6b}$ = 1.1 Hz, 1H, H-3b), 7.10 (s, 1H, H-4), 7.09 (dd, $^{3}J_{H\text{-}4d\text{-}H\text{-}3d}$ = 8.3 Hz, $^{4}J_{H\text{-}4d\text{-}H\text{-}6d}$ = 2.4 Hz, 1H, H-4d), 6.86 (dd, $^{3}J_{H\text{-}3a\text{-}H\text{-}4a}$ = 8.1 Hz, $^{5}J_{H\text{-}3a\text{-}H\text{-}6a}$ = 3.2 Hz, 1H, H-3a), 2.37 (s, 3H, C*H*$_3$-a), 2.27 (s, 3H, C*H*$_3$-b), 2.23 (s, 3H, C*H*$_3$-b), 2.12 (s, 3H, C*H*$_3$-d) ppm.

^{13}C{^{1}H} NMR (176 MHz, CD_2Cl_2): δ = 156.7 (d, $^{3}J_{C\text{-}2b\text{-}P}$ = 4.6 Hz, 1C, C-2b), 153.8 (d, $^{3}J_{C\text{-}2a\text{-}P}$ = 5.0 Hz, 1C, C-2a), 153.6 (s, 1C, C-2d), 153.6 (s, 1C, C-2c), 151.1 (s, 1C, C-5), 150.5 (s, 1C, C-6b), 150.3 (s, 1C, C-6a) 150.0 (s, 1C, C-6d), 149.9 (s, 1C, C-6c), 146.9 (d, $^{1}J_{C\text{-}2\text{-}P}$ = 9.8 Hz, 1C, C-2), 144.6 (d, $^{1}J_{C\text{-}6\text{-}P}$ = 8.9 Hz, 1C, C-6), 143.7 (d, $^{2}J_{C\text{-}3\text{-}P}$ = 6.3 Hz, 1C, C-3), 138.9 (s, 2C, C-4b, C-4c), 136.6 (s, 1C, C-4d), 136.4 (s, 1C, C-4a), 134.2 (d, $^{3}J_{C\text{-}4\text{-}P}$ = 16.9 Hz, 1C, C-4), 133.9 (s, 1C, C-5c), 133.6 (s, 1C, C-5d), 133. 4 (s, 1C, 5b) 133.1 (s, 1C, 5a) 128.5 (d, $^{4}J_{C\text{-}3c\text{-}P}$

= 7.9 Hz, 1C, C-3c), 127.8 (d, $^{4}J_{C\text{-}3d\text{-}P}$ = 5.3 Hz, 1C, C-3d), 125.6 (s, 1C, C-3a), 124.9 (s, 1C, C-3b), 18.4 (s, 3C, *C*H_3-b), 18.3 (s, 3C, *C*H_3-a), 18.3 (s, 3C, *C*H_3-c), 18.0 (s, 3C, *C*H_3-d) ppm.

^{31}P NMR (162 MHz, CD_2Cl_2): δ = 152.2 ppm.

HRMS-ESI: *m/z* calculated for $C_{58}H_{50}Cu_3I_2N_8P^+$ [(PP)$_2$(Cu_3I_2)]$^+$: 1362.9611 Found: 1362.9523

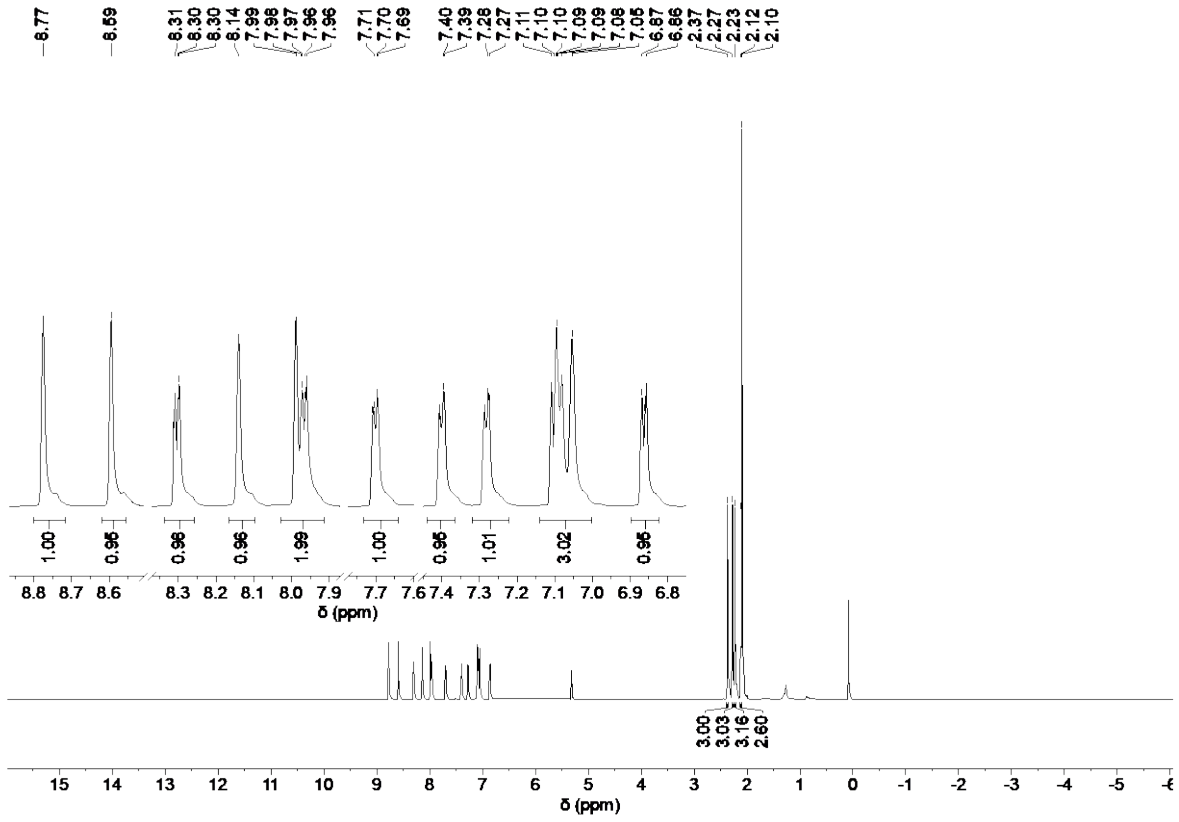

Figure S2.29: ^{1}H NMR spectrum (CD_2Cl_2) of **7b**.

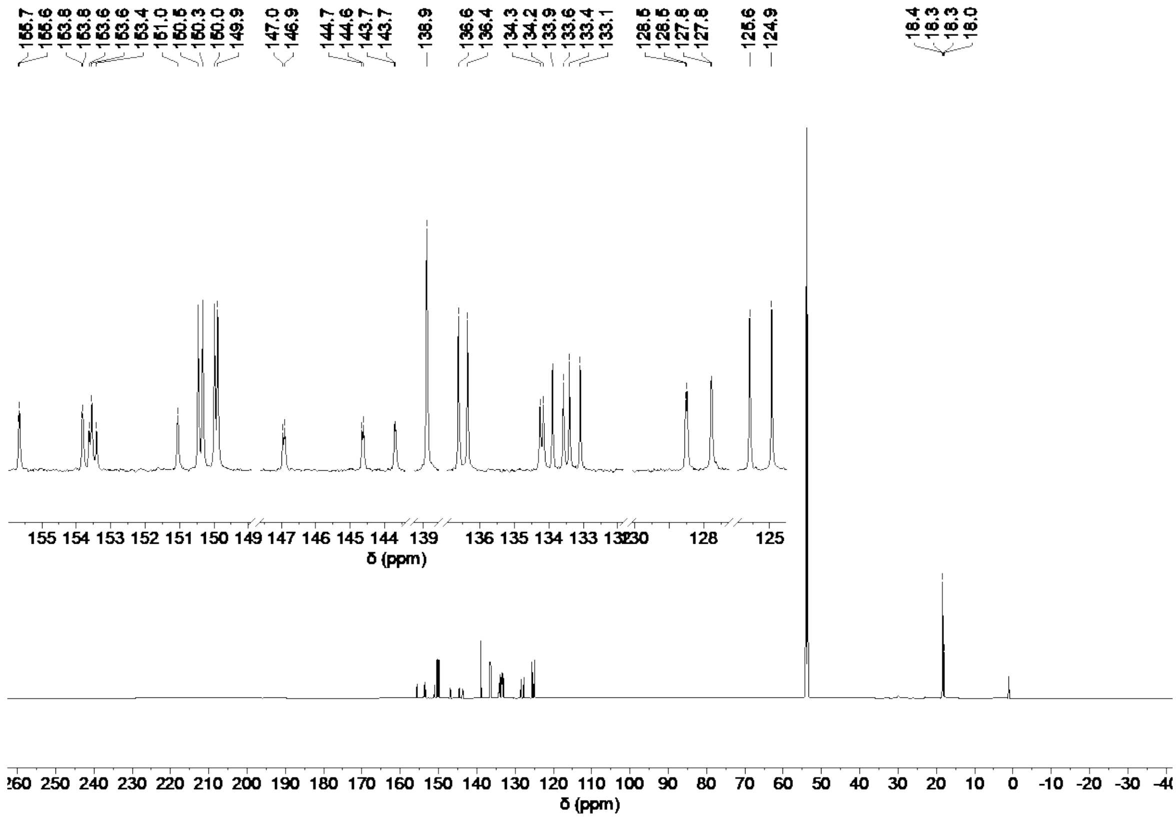

Figure S2.30: $^{13}C\{^{1}H\}$ NMR spectrum (CD_2Cl_2) of **7b**.

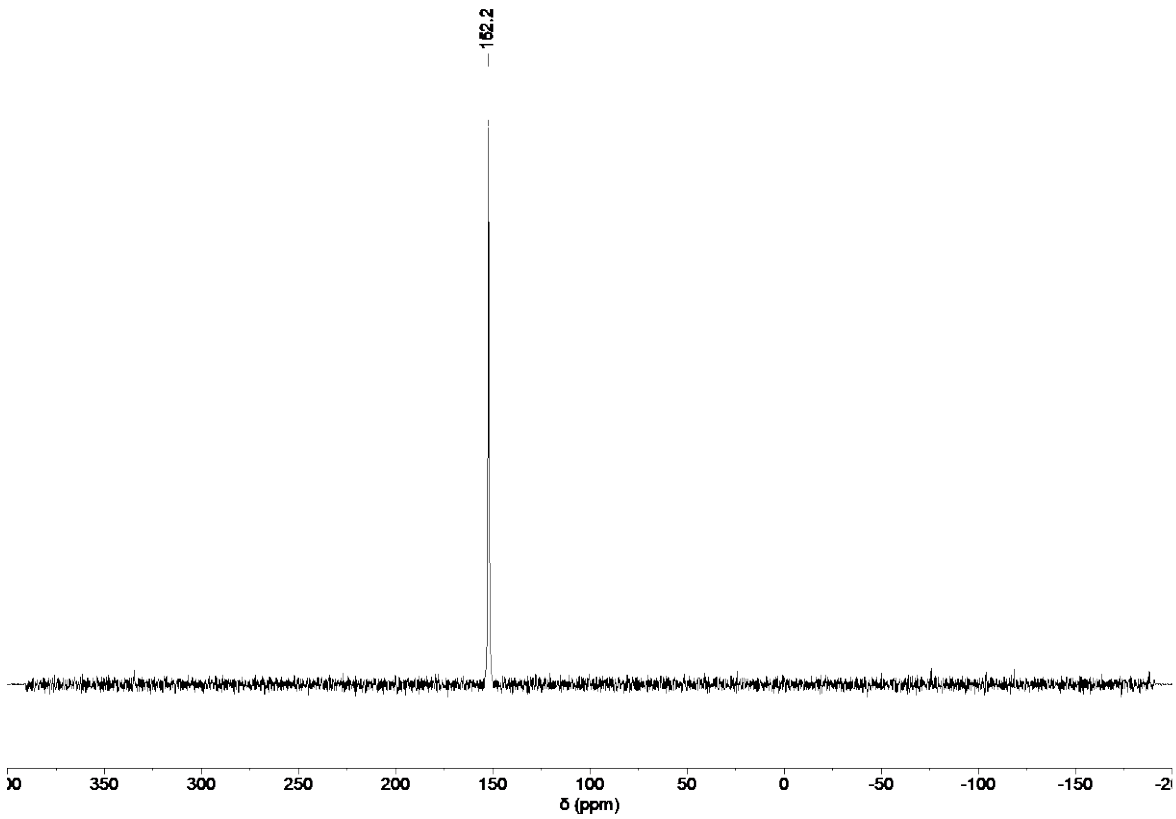

Figure S2.31: ^{31}P NMR spectrum (CD_2Cl_2) of **7b**.

3. UV/Vis Analysis

3.1 UV/Vis Details

For the UV/Vis measurements, the UV/Vis spectrometer '*Lambda 465'* of *PerkinElmer* was used, equipped with 'UV Lab' to process the raw data. The plotted spectra were prepared using Origin 2023.[i]

For the measurements, a solution of **2a** in CH_2Cl_2 with a concentration of c[**2a**] = $2.0 \cdot 10^{-5}$ mol/L (Solution 1) was prepared. To add different equivalents of H_2O to the phosphinine **2a**, a stock solution of H_2O in CH_2Cl_2 with the same concentration of c[H_2O] = $2.0 \cdot 10^{-5}$ mol/L (Solution 2) was used. 7 different samples with increasing equivalents of H_2O were prepared by adding the respective volume of solution 2 to 20 µL of solution 1, and topping-up the samples with CH_2Cl_2 to a total volume of 2 mL. (**Table S1**) After a reaction time of 16 hours, the solutions' UV/Vis spectra were then recorded to investigate the sensitivity of the water activation and the possible use of **2a** as a water sensor. (**Figure S3.2**)

For means of comparison, a separate solution of freshly synthesized **4a**/**5a** in CH_2Cl_2 was diluted to c[**4a**/**5a**] = 1.4 10^{-5} mol/L and using a volume of 20 µL on a total volume of 2 mL its UV/Vis spectrum recorded as well. (**Table S2**)

Table S3.1: Sample preparation of **2a.**

Sample	H_2O Equiv.	V (Solution 1) [µL]	V (Solution 2) [µL]	V (CH_2Cl_2) [µL]
1	0.0	20	–	1980
2	0.5	20	10	1970
3	1.0	20	20	1960
4	2.0	20	40	1940
5	3.0	20	60	1920
6	5.0	20	100	1880
7	10	20	200	1780

Table S3.2: Sample preparation of **4a/5a.**

Sample	V (Solution of 4a/5a) [µL]	V (CH_2Cl_2) [µL]
8	20	1980

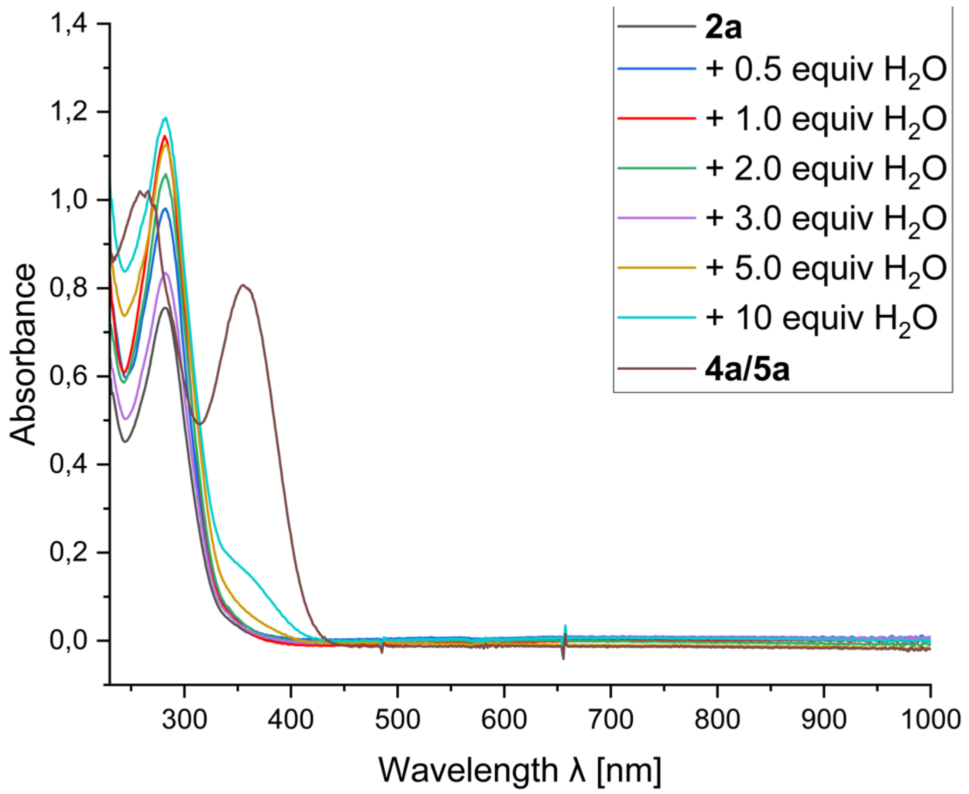

Figure S3.1: Full UV/Vis spectra of all samples.

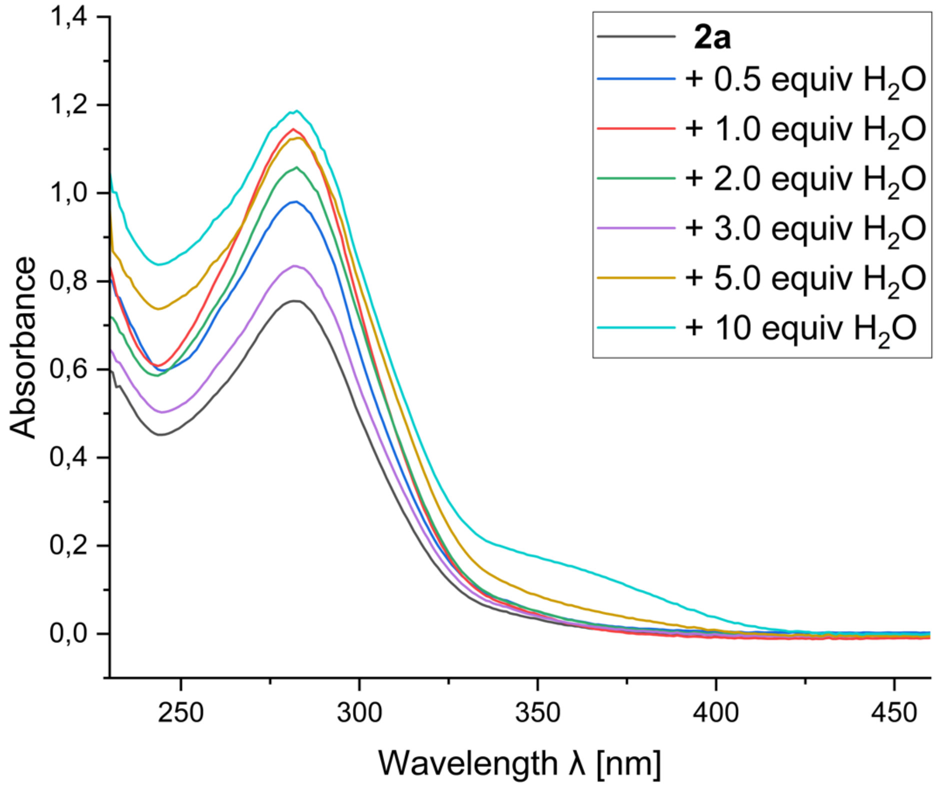

Figure S3.2: Close-up of absorption range for the samples 1-7

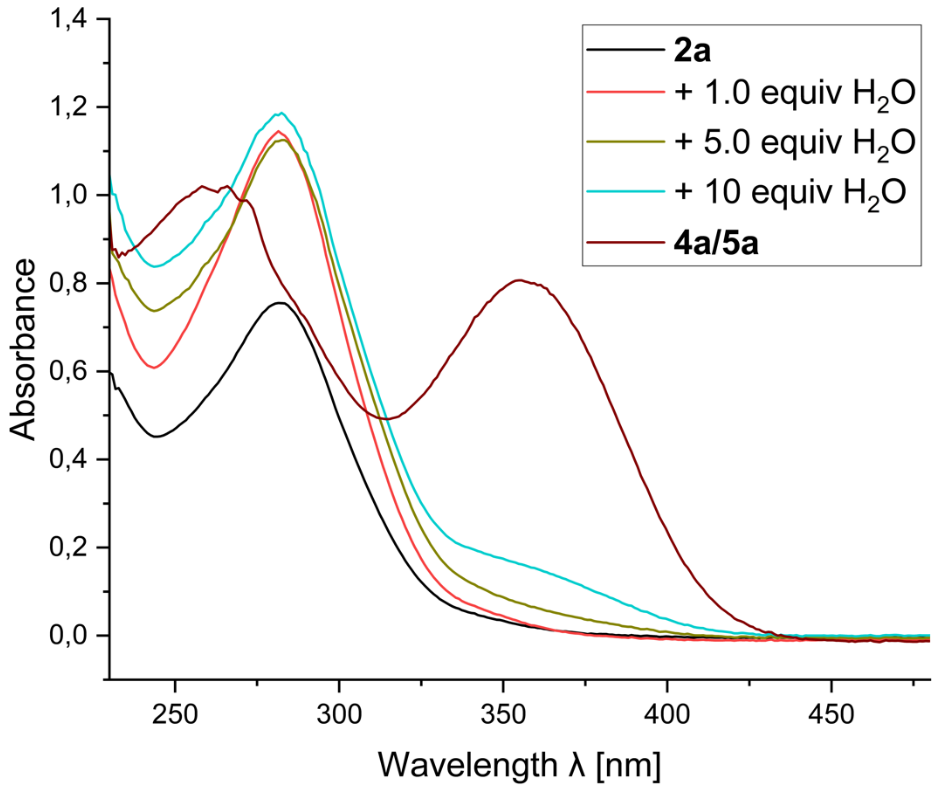

Figure S3.3: Absorption of the samples 3, 6 and 7 compared to **4a**/**5a**.

The phosphine **2a** shows one characteristic absorption in the near UV-range at 281 nm. This is a typical absorption for λ^3-phosphinines.[14] In contrast, **5a** shows two absorptions, one shifted to lower frequencies at 355 nm and one shifted to higher frequencies at 264 nm. (**Figure S3.3**)

While the obtained spectra of **2a** with 0.5 equivalents of H_2O, as well as 1.0 equivalents, 2.0 equivalents and 3.0 equivalents, do not show significant changes in their absorption features, the measurements with 5.0 equivalents and 10 equivalents of H_2O start showing the expected shoulders at 355 nm, indicating their conversion to the water adduct **4a**/**5a**. The low conversion is most likely explicable by the notably lower concentration of **2a** in CH_2Cl_2 compared to the NMR spectroscopic measurements, resulting in a significantly longer reaction time needed than was employed herein. While these results do indicate that **2a** reacts with water even at very low concentrations, they also show that it is rather unsuitable in an application as a real-time water sensor. Apart from the prolonged reaction time itself, the error in concentration that would ensue from evaporation of the CH_2Cl_2 over time and during working steps (as can already be partially observed in the absorption maxima of samples 1-7) would inhibit its use in the quantification of unknown water concentrations.

4. Crystallographic Data

4.1 Crystallographic Details

Single crystal x-ray diffraction data was collected on a Bruker D8 Venture fitted with a Photon II CMOS Detector with Mo *K*α radiation (λ = 0.71073 Å) from an *IμS* micro-source, performing φ-and ω-scans. Data collection and processing was handled using the Bruker APEX3 software package. Structures were solved within Olex2[15] by dual space iterative methods (SHELXT)[16] and all non-hydrogen atoms refined by full-matrix least-squares on all unique F^2 values with anisotropic displacement parameters (SHELXL).[17] All non-hydrogen atoms were refined anisotropically, all hydrogen atoms were included into the model at geometrically calculated positions and refined using a riding model. The isotropic displacement parameters of all hydrogen atoms were fixed to 1.2 times the *U* value of the atoms they are linked to (1.5 times for methyl groups). Structures were checked with checkCIF.[18] Selected crystallographic can be found in **Table S1** below. The CCDC entries 2302172 (**5b**), 2304990 (**2a**), 2304991 (**2b**), 2304992 (**E**) 2305048 (**4b**) and 2305850 (**7a(Li)**) contain the supplementary crystallographic data for this article. These data can be obtained free of charge from The Cambridge Crystallographic Data Centre via www.ccdc.cam.ac.uk/structures.

Tabel S4.1: Selected crystallographic data

	**2a**	**2b**	**4b**	**6a(Li)**
CCDC number	2304990	2304991	2302172	2305850
Empirical formula	$C_{25}H_{17}N_4P$	$C_{36}H_{32}N_4P$	$C_{29}H_{27}N_4OP$	$C_{103.44}H_{78.88}Cl_{6.88}Li_4N_{16}O_4P_4$
Formula weight	404.39	551.62	478.51	2005.52
Temperature [K]	100.0	150.0	102.0	150
Crystal system	Monoclinic	Triclinic	Triclinic	Tetragonal
Space group	$P2_1/c$	$P\bar{1}$	$P\bar{1}$	$I\bar{4}$
a [Å]	6.0233(2)	6.04355(9)	10.539(2)	22.4512(8)
b [Å]	7.2655(2)	10.8813(2)	10.9798(18)	22.4512(8)
c [Å]	21.2045(6)	11.4035(2)	11.7826(15)	10.3077(5)
α [°]	90	102.9934(7)	92.457(9)	90
β [°]	93.2936(9)	95.8207(6)	108.816(4)	90
γ [°]	90	93.2149(6)	103.145(4)	90
Volume [Å^3]	972.33(5)	724.54(2)	1246.7(4)	5195.7(4)
Z	2	1	2	2

Reflections collected	25237	32667	15240	20616
Independent reflections (R_{int})	2955 (0.0303)	3310 (0.0352)	4583 (0.0330)	4550 (0.0999)
R_1 [I ≥ 2σ (I)]	0.0591	0.0672	0.0454	0.0505
wR_2 (all data)	0.1350	0.1388	0.1045	0.1300

Tabel S4.2: Selected crystallographic data

	**7b**	**E**
CCDC number	2305048	2304992
Empirical formula	$C_{59}H_{52}Cl_2Cu_4I_4N_8P_2$	C_{14} H_{12} N_2
Formula weight	1767.68	208.26
Temperature [K]	105.0	100.00
Crystal system	Monoclinic	Monoclinic
Space group	$P2_1$/n	$C2$/c
a [Å]	19.1051(8)	8.4144(5)
b [Å]	17.5837(7)	9.2664(5)
c [Å]	19.9459(7)	14.4244(10)
α [°]	90	90
β [°]	99.8850(10)	103.942(2)
γ [°]	90	90
Volume [Å^3]	6601.1(4)	1091.55(12)
Z	4	4
Reflections collected	169316	16349
Independent reflections (R_{int})	15131 (0.0515)	1658 (0.0340)
R_1 [I ≥ 2σ (I)]	0.0386	0.0718
wR_2 (all data)	0.0929	0.2543

Additional refinement details for 2a

The structure exhibits inversion disorder about the central phosphinine ring. To obtain a satisfactory model for the phosphinine core, the C atoms bound directly to the two crystallgoraphically unique pyridyl rings were split and all atoms comprising the central phosphinine unit were given an occupancy of 0.5. To conform with other structures reported containing phosphinine moieties, the structure was then grown and the two carbons furthest from P1, (to account for the longer P-C bonds) and the two carbons closest to C3 (to account for the shorter C-C bonds) were selected and using the command 'part -1 -c' in Olex2 to generate a full two-fold disorder of the central phosphinine ring was formed. The carbons bound to the pyridyl units (C1, C2, C4, C5) had their thermal parameters constrained (EADP) due to substantial spatial overlap of the ellipsoids with its inversion component.

Additional refinement details for 2b

The structure crystallises in the triclinic space group P-1 and features half a molecule of 6,6',6'',6'''-(phosphinine-2,3,5,6-tetrayl)tetrakis(3-methylpyridine) in the asymmetric unit. The central phosphinine ring core is situated across an inversion centre and disordered by symmetry with occupancy 0.5 as per symmetry requirement. To obtain a suitable model for the phosphinine core, the carbons adjacent to the pyridyl ring were split and all atoms forming the central phosphinine unit were given an occupancy of 0.5. To fit with other phosphinine moieties, the structure was then grown and the two carbons furthest from P1, (to account for the longer P-C bonds) and the two carbons closest to C3 (to account for the shorter C-C bonds) were selected and using the command 'part -1 -c' in Olex2 the central phosphinine ring was formed. The carbons bound to the pyridyl units (C1, C2, C4, C5) had their thermal parameters constrained (EADP) due to substantial spatial overlap of the ellipsoids with the second component.

A toluene molecule of crystallisation is also present in the crystal which features disorder by inversion and was modelled as a twofold whole body disordered site by placing in part -1. The occupancy of this molecule refined to 0.498(3), which was later constrained to 0.5 in the final model giving a total solvate occupancy of unity. The six-carbon central core of the toluene molecule was constrained using AFIX 66 to ensure a chemically sensible geometry. A FLAT restraint was also used across the entire seven carbon skeleton of the toluene molecule, in addition to the displacement parameter restraint combination SIMU/RIGU about the entire molecule.

The erroneous reflection (0,1,0) was removed during refinement due to partial obscuration by the beamstop.

Additional refinement details for 4b

The asymmetric unit contains one molecule of the target complex and a highly disordered equivalent of co-crystalised DCM. Attempts to model the solvent were unsuccessful and so SQUEEZE[19] was implemented. A solvent mask was calculated and 180 electrons were found in a volume of 1158 Å^3 in 3 voids per unit cell. This is consistent with the presence of 1 CH_2Cl_2 per asymmetric unit which account for 168 electrons per unit cell.

Additional refinement details for 6a(Li)

The structure presented was isolated as a tetrameric coordination complex, crystallising in the tetragonal space group $I\bar{4}$ with one quarter of the tetramer itself comprising the asymmetric unit and a molecule of dichloromethane of crystallisation. The dichloromethane molecule features a minor whole body disorder and was split into parts '1' and '2' for the sites 'C1...Cl2' and 'C1A Cl2A' respectively. Though a residual electron density squeeze calculation prior to modelling the dichloromethane sites suggested 100% cumulative occupancy across the two sites, the thermal parameters were unacceptable and suggested either additional disorder or a net occupancy slightly less than one. The occupancies of the dichloromethane sites were then refined against net occupancies between 0.8 and 1.0, with 0.86 yielding the best convergence and lowest refinement parameters. In this model, the individual occupancies of the dichloromethane sites '1' and '2' refined to 0.52(3) and 0.34(3) respectively. Several restraints were applied to ensure both dichloromethane sites conformed to sensible molecular geometries. DFIX 1.77 Å restraints were placed between C1-Cl1 and C1-Cl2, and all 1,2- C-Cl distances were subject to a SADI same distance restraint. All 1,3- Cl-Cl distances were restrained through combined use of DANG 2.92 and a SADI same distance restraint across both sites. Combined displacement parameter restraints SIMU and RIGU were also used across both sites. Finally, EADP constraints were applied linking the complementary atoms of the two different sites to 'C1,C1A' and 'Cl1,Cl1A' as the atoms observe a significant portion of spatial overlap in the structure. There is no further disorder present in the model, and all non-H atoms were refined anisotropically with all H-atoms placed in calculated positions and allowed to refine using a riding model.

The structure is an inversion twin, and refined against the twin law (-1,0,0,0,-1,0,0,0,-1) with a final BASF of 0.07(3). An extinction correction was refined to 0.0008(2). Two bad reflections were removed from the final refinement, (0,2,0) and (1,3,0), however no systematic origin of the cause of the error could be ascertained. The data was truncated at 0.84 Å, as the crystal diffracts only very weakly at higher angle, which may be a consequence of the onset of loss of solvent leading to a drop in crystallinity. The final R1 refined to 0.0505.

Additional refinement details for E

The structure crystallises in the monoclinic space group C2/c, with the asymmetric unit comprised of half a molecule of 1,2-bis(5-methylpyridin-2-yl)ethyne disordered by symmetry across an inversion centre. The final model presented yields relatively poor refinement parameters, which is believed to be due to the presence of a secondary minor component also exhibiting whole body disorder by symmetry situated across the same geometric space of the major component in the crystal, evidence of which is present in the residual fourier difference map. It was not viable to model this disordered minor component sensibly, given that the structure is comprised only of light atoms, and this would result in an unsatisfactorily low data-to-parameter ratio. The model is instead presented as is as a twofold disordered structure. Extensive efforts were made to look for additional signs of twinning to account for the poor quality of the refinement, but there was no evidence in the raw data or most disagreeable reflections for the existence of either pseudo- or non-merohedral twinning at play.

The erroneous reflection (3,3,9) was re-improved from the model, however no systematic origin of this error could be ascertained.

2b

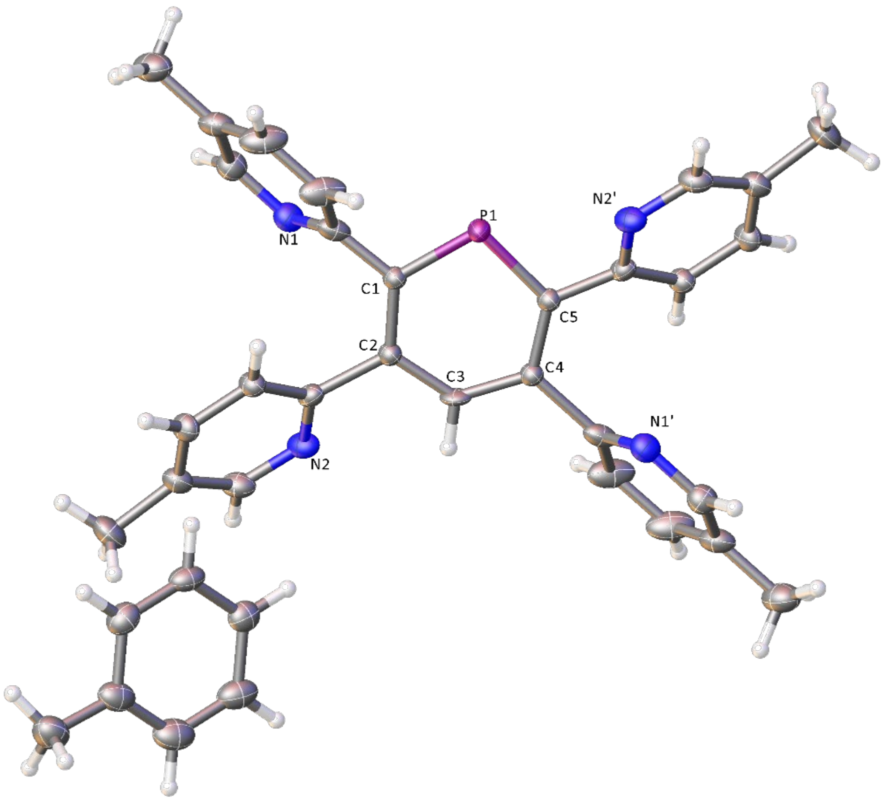

Figure S4.1: Structure of **2b** in the solid state. Thermal ellipsoids given at 50% probability. Image shows co-crystallised toluene and one orientation of the central phosphinine core. Atoms marked with ‘ are symmetry generated using the following equation: 2-X, 1-Y, -Z.

E

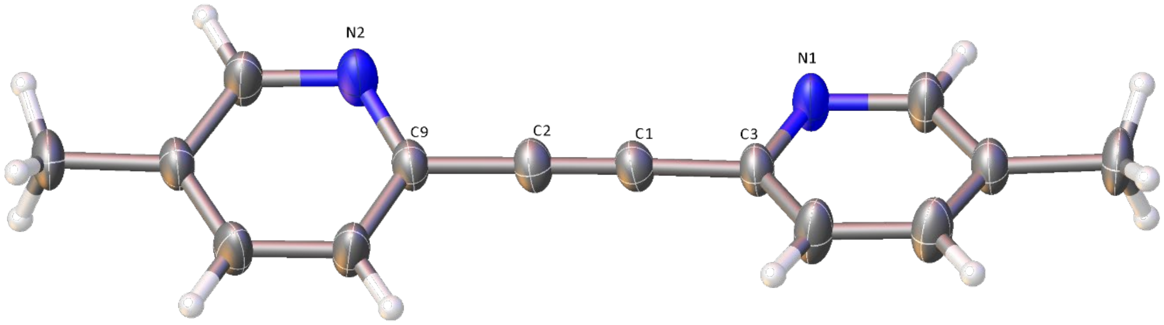

Figure S4.2: Structure of **E** in the solid state. Thermal ellipsoids given at 50% probability.

5. References

[1] J. Tao, J. P. Perdew, V. N. Staroverov, G. E. Scuseria, *Phys Rev Lett* **2003**, *91*, 146401.

[2] F. Weigend, R. Ahlrichs, *Physical Chemistry Chemical Physics,* **2005**, *7*, 3297–3305.

[3] F. Weigend, *Physical chemistry chemical physics,* **2006**, *8*, 1057–1065.

[4] Y. Zhao, N. E. Schultz, D. G. Truhlar, *J Chem Theory Comput,* **2006**, *2*, 364–382.

[5] L. Goerigk, A. Hansen, C. Bauer, S. Ehrlich, A. Najibi, S. Grimme, *Physical Chemistry Chemical Physics,* **2017**, *19*, 32184–32215.

[6] J. Tomasi, B. Mennucci, R. Cammi, *Chem Rev,* **2005**, *105*, 2999–3094.

[7] S. Grimme, J. Antony, S. Ehrlich, H. Krieg, *J Chem Phys,* **2010**, *132*.

[8] A. E. Brown, B. E. Eichler, *Tetrahedron Lett.,* **2011**, *52*, 1960–1963.

[9] M. Doux, L. Ricard, F. Mathey, P. L. Floch, N. Mézailles, *Eur J Inorg Chem,* **2003**, 687–698.

[10] K. Clauss, H. Bestian, *Justus Liebigs Ann Chem,* **1962**, *654*, 8–19.

[11] P. Heretsch, S. Rabe, A. Giannis, *Org Lett.,* **2009**, *11*, 5410–5412.

[12] K. M. Doxsee, J. B. Farahi, *J Am Chem Soc,* **1988**, *110*, 7239–7240.

[13] A. E. Brown, B. E. Eichler, *Tetrahedron Lett.*, **2011**, *52*, 1960-1963.

[14] G. Pfeifer, F. Chahdoura, M. Papke, M. Weber, R. Szűcs, B. Geffroy, D. Tondelier, L. Nyulászi, M. Hissler, C. Müller, *Chem. Eur. J.* **2020**, *26*, 10534–10543

[15] G. Körbich, *Angew. Chem. Int. Ed.,* **1972**, *11*, 473-485.

[16] O. V. Dolomanov, L. J. Bourhis, R. J. Gildea, J. a. K. Howard and H. Puschmann, *J Appl Cryst*, **2009**, *42*, 339–341.

[17] G. M. Sheldrick, *Acta Cryst A*, **2015**, *71*, 3–8.

[18] G. M. Sheldrick, *Acta Cryst C*, **2015,** *71*, 3–8.

[19] The facility "CheckCIF," can be found at http://checkcif.iucr.org.

[20] A. L. Spek, *Acta Cryst C*, **2015,** *71*, 9–18.

i
